# Establishing performance metrics for quantitative non-targeted analysis: a demonstration using per- and polyfluoroalkyl substances

**DOI:** 10.1007/s00216-023-05117-4

**Published:** 2024-01-30

**Authors:** Shirley Pu, James P. McCord, Jacqueline Bangma, Jon R. Sobus

**Affiliations:** 1 US Environmental Protection Agency, Office of Research and Development, Center for Computational Toxicology and Exposure, 109 TW Alexander Dr., Research Triangle Park, NC 27711 USA; 2 Oak Ridge Institute for Science and Education (ORISE) Participant, 109 TW Alexander Dr., Research Triangle Park, NC 27711 USA; 3 US Environmental Protection Agency, Office of Research and Development, Center for Environmental Measurement and Modeling, 109 TW Alexander Dr., Research Triangle Park, NC 27711 USA

**Keywords:** HRMS, Accuracy, Uncertainty, Reliability, Bootstrapping

## Abstract

Non-targeted analysis (NTA) is an increasingly popular technique for characterizing undefined chemical analytes. Generating quantitative NTA (qNTA) concentration estimates requires the use of training data from calibration “surrogates,” which can yield diminished predictive performance relative to targeted analysis. To evaluate performance differences between targeted and qNTA approaches, we defined new metrics that convey predictive accuracy, uncertainty (using 95% inverse confidence intervals), and reliability (the extent to which confidence intervals contain true values). We calculated and examined these newly defined metrics across five quantitative approaches applied to a mixture of 29 per- and polyfluoroalkyl substances (PFAS). The quantitative approaches spanned a traditional targeted design using chemical-specific calibration curves to a generalizable qNTA design using bootstrap-sampled calibration values from “global” chemical surrogates. As expected, the targeted approaches performed best, with major benefits realized from matched calibration curves and internal standard correction. In comparison to the benchmark targeted approach, the most generalizable qNTA approach (using “global” surrogates) showed a decrease in accuracy by a factor of ~4, an increase in uncertainty by a factor of ~1000, and a decrease in reliability by ~5%, on average. Using “expert-selected” surrogates (*n* = 3) instead of “global” surrogates (*n* = 25) for qNTA yielded improvements in predictive accuracy (by ~1.5×) and uncertainty (by ~70×) but at the cost of further-reduced reliability (by ~5%). Overall, our results illustrate the utility of qNTA approaches for a subclass of emerging contaminants and present a framework on which to develop new approaches for more complex use cases.

**Graphical Abstract:**

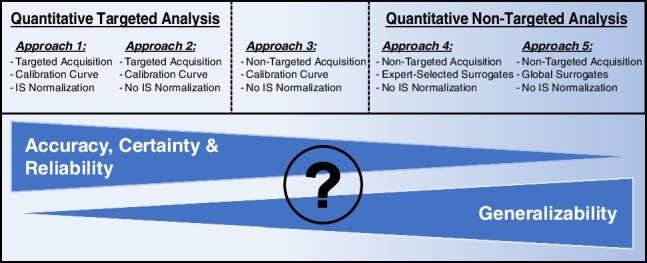

**Supplementary Information:**

The online version contains supplementary material available at 10.1007/s00216-023-05117-4.

## Introduction 

Fully assessing environmental exposures requires identifying and quantifying many unique chemical species [1, 2]. Targeted analytical methods, often based on mass spectrometry, have long been the gold standard for quantifying known analytes. These targeted methods, however, cannot characterize unexpected or unknown chemicals that are present in consumer products, environmental samples, and biological matrices [3, 4]. Non-targeted analysis (NTA) approaches, based on high-resolution mass spectrometry (HRMS), are therefore increasingly used to examine samples more holistically with respect to contaminants-of-emerging concern (CECs) [5–7].

The large number of molecular features observed in NTA-HRMS studies has driven an initial focus on identifying novel compounds; considerably less focus has been placed on quantifying these compounds in support of risk-based evaluations [8]. In a best case scenario, an initial provisional quantification could be performed on identified priority analytes, with post hoc targeted quantitation conducted after acquiring reference standards. It is impractical, however, to acquire reference standards and develop targeted methods for the hundreds or thousands of chemicals observed in any given NTA experiment. Given this daunting challenge, a need exists for quantitative NTA (qNTA) strategies that can yield defensible quantitative estimates to support provisional decisions in the absence of post hoc targeted evaluation.

In this proof-of-concept study, we first defined new performance metrics which enable comparison of the accuracy, uncertainty, and reliability of targeted and qNTA approaches. Using these metrics, we assessed the performance of several targeted and qNTA approaches using a set of known per- and polyfluoroalkyl substances (PFAS). PFAS are a varied chemical class, containing historically well-characterized “legacy” chemicals, such as perfluorooctanoic acid (PFOA) and the related perfluorinated alkyl acids, as well as numerous emerging chemicals with limited extant information. Legacy PFAS are readily examined using widely available reference standards and validated methods (e.g., EPA Method 533 [9]) and have been a focus of multiple regulatory efforts. Emerging PFAS, on the other hand, are an expanding group of thousands of chemicals [10, 11] with new species frequently detected and identified via NTA experiments [7, 12, 13]. Recent literature has indicated emerging PFAS as a major component of total PFAS exposure [14, 15]. Since analytical standards and internal standards (IS) are not readily available for many of these chemical species, a clear need exists for defensible quantitative approaches that will support provisional risk evaluations.

Herein, we present (1) background information on the utilized targeted and qNTA approaches; (2) analytical steps for the collection of a PFAS test dataset; (3) methods for applying targeted and qNTA approaches to the PFAS dataset; (4) metrics for comparing accuracy, uncertainty, and reliability of quantitation approaches; and (5) a comparison of performance metrics across the targeted and qNTA approaches. The overall objectives of this work are to describe and compare the quantitative performance of targeted and qNTA approaches on a test set of PFAS and to investigate the factors influencing performance.

## Background

### Empirical response factors

Electrospray ionization mass spectrometry (ESI–MS) is a premier technique for chemical analysis due to its sensitivity and specificity. Quantitation relies on the defined relationship between the concentration of an analyte and the measured instrument response of that analyte at the known concentration. In experimental terms, the quotient of a measured chemical signal (e.g., ion abundance, which may be quantified as the integrated peak area of a compound) and a known concentration is termed a “response factor” (RF) and is equivalent to the slope of an ideal (i.e., errorless) linear calibration curve. Response factors can vary widely between chemicals but are expected to be fairly stable for any specific chemical within the linear dynamic range of the instrumentation and method. Errors affecting RF stability may be systematic or random and stem from small changes in matrix effects, elution time, solvent composition, peak shape, and instrument sensitivity. Calibration curves using multi-point dilutions, replicate injections, and internal standard correction are often used to quantify and account for these sources of error in targeted studies, as described below.

### Calibration curves for targeted quantification

#### General structure and use

For traditional chemical quantification, a calibration curve is constructed which relates a sample’s chemical concentration (*X* axis) to an observed ion abundance associated with that chemical (*Y* axis). Mathematically, this curve is not used in its standard form (estimating ion abundance at a specific concentration) but is instead used for “inverse estimation” (estimating concentration at a specific ion abundance). The inverse estimation yields the concentration of a chemical (with inverse confidence limits) in a new sample given an observed ion abundance. When an internal standard (generally stable isotope-labeled) is available, the observed ion abundance of a target compound can be divided by that of the internal standard to obtain a *normalized ion abundance* (often referred to as a “response ratio”). Normalization using an internal standard corrects for experimental variance, as changes affecting the analyte of interest should equally affect the internal standard. Proper internal standard adjustment yields improved accuracy and precision for inverse predictions.

#### Modeling approaches

Traditional linear calibration curves are constrained within the linear dynamic range of a chemical, instrument, and method. However, depending on the ranges of concentrations and ion abundances observed, the relationship between ion abundance and concentration may not be linear. Furthermore, liquid chromatography mass spectrometry (LC–MS) calibration curves frequently exhibit heteroscedastic errors since the absolute variance of ion abundance measurements generally increases with rising concentration even as relative variance remains consistent. Mathematical models can account for non-ideal calibration behavior. For example, polynomial models can fit non-linear behavior, weighted regression can account for heteroscedasticity, and logarithmic transformation can account for either or both. Log transformation can further allow calibrant concentrations to be more equally spaced on the *X* axis, thus ensuring more uniform influence of individual data points on calibration curve parameter estimates (i.e., slope and intercept). A more complete discussion of the benefits of different mathematical treatments of calibration data can be found in Groff et al. 2022 [16]. Both weighted quadratic models and linear models of log-transformed data were considered for the current work, with log-linear models ultimately selected for use due to their mathematically convenient properties for inverse estimation.

### Quantitative non-targeted analysis approaches

Quantitative NTA approaches rely on calibration data from surrogate analytes to estimate concentrations of detected analytes. For any detected analyte, one or more surrogates may be selected based on an expectation of RF similarity between the surrogate(s) and the analyte. Suitable surrogates may be chosen based on multiple criteria, including chemical structure similarity, chemical class assignment, or chromatographic elution time [17–19]. Surrogate selection methods are influenced by the information available about the analytes of interest. If a chemical structure is known with reasonable certainty, the chemical’s ionization efficiency (directly related to its RF) may be predicted [20, 21] and used as the basis for concentration estimation [16, 22]. Still, even model-based ionization efficiency estimates must be calibrated to the experimental platform using data from representative surrogates.

Modeling techniques now exist to estimate the uncertainty associated with qNTA predictions, as described in detail below (see “Quantitative non-targeted analysis” section). While uncertainty is present in nearly all quantitative analytical estimates (including those based on targeted measurements), additional uncertainty surrounds qNTA predictions given the necessary use of surrogate calibration data. Understanding the sources and magnitude of numerical uncertainty for qNTA estimates is a focal point of this investigation.

## Methods

**Fig. 1:**
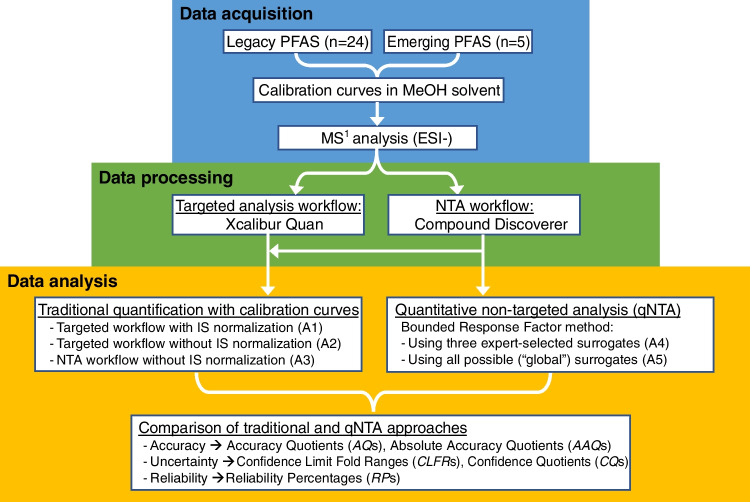
Workflow of data acquisition, processing, and analysis for comparison of performance metrics between targeted and qNTA approaches

Five different experimental approaches (A1–A5) were compared in an effort to understand and quantify sources of error in qNTA applications. These approaches differed in their data acquisition, processing, and mathematical modeling procedures (Fig. 1). The first three approaches (A1–A3) all used calibration curves for inverse prediction but differed in their data acquisition and processing procedures. Approaches 1 and 2 both used targeted data acquisition and processing, but only A1 used internal standard-normalized ion abundances. Approach 3 used non-targeted acquisition and processing without internal standard normalization. The last two approaches (A4 and A5) used bootstrap simulation to estimate population RF percentile values. Approach 4 used a subset of available surrogate chemicals, chosen based on expert chemical intuition, while A5 used all available surrogate chemicals as the population for the bootstrap simulation. Specific methods used for each procedure are described in detail below.

### Data acquisition

Samples were prepared from two mixed PFAS stock solutions. The first consisted of 29 PFAS compounds prepared in 70:30 H_2_O:MeOH (methanol). Twenty of the PFAS had available matched stable isotope-labeled internal standards, which were prepared as a second internal standard stock. In brief, the PFAS compounds included a homologous series of perfluorinated carboxylic acids (C4–C14) and sulfonic acids (C4–C10) as well as a selection of fluorotelomer sulfonates, sulfonamides, and fluoroethers. Specific details on the 29 PFAS standards and 20 labeled internal standards are available in Table S1.

Ten milliliter standard samples were prepared from the PFAS stock to facilitate the creation of a nine-point calibration curve with ~ twofold point spacing, ranging from 0.98 to 250 ng/mL of each unlabeled PFAS. Each calibration point was prepared independently rather than from serial dilution. Each sample received spikes of the labeled internal standard mix at 50 ng/mL per sample. Double (analytical) blanks consisting of only water and methanol were prepared to ascertain background noise; visual inspection revealed the blanks to be interference-free. All samples and blanks were evaporated under nitrogen stream for 15 min (until they contained < 400 µL volume) and then transferred to vials for analysis.

Sample analysis was performed on a Thermo Scientific Vanquish ultra-performance liquid chromatograph (UPLC) and a Thermo Scientific Orbitrap Fusion. The ionization source was electrospray ionization (ESI) operating in negative mode. Full scan (MS^1^) mass spectra were collected. Samples were run in a single batch with each sample having three sequential replicate injections. A 20-min linear mobile phase gradient was used for separation, beginning with solvent A, 5% acetonitrile (ACN) v/v with 2.5 mM ammonium acetate in water, and ending with solvent B, 95% v/v ACN, 2.5 mM ammonium acetate. Additional details on the instrumental method are provided in Supporting Information 1.0–2.0.

### Data processing

#### Targeted analysis workflow (A1–A2)

The exact masses of compounds of interest and their expected elution times were entered as a quantitative method in the Thermo Xcalibur Quan software (Thermo Fisher Scientific, Waltham, MA). Extracted ion chromatograms were generated from [M-H]^−^ ions plus [M-CO2-H]^−^ ions, when applicable. Mass tolerance was set to 6 ppm for peak integration. Lists of exact masses for integration and additional details are available in Supporting Information 3.0.

#### Non-targeted analysis workflow (A3–A5)

Data were processed using Compound Discoverer (Thermo Fisher Scientific) based on a standard NTA data processing workflow [23]. This workflow performed compound peak picking and alignment for all samples in the batch, data filtering for noise/background features, compound integration, and tentative chemical assignment based on formula generation and library and mass list searching. Mass tolerance was set to 10 ppm for chemical assignments. Tentative chemical identifications were provided by Compound Discoverer based on matching to user-provided mass lists and to Thermo mzCloud. These identifications were manually curated. Isomeric peaks were combined to yield a single compound signal. In contrast to the targeted method, Compound Discoverer used the abundance of the main (i.e., most abundant) ion peak for each identified species, resulting in area differences between the targeted and NTA workflows. A complete description of the NTA workflow is available in Supporting Information 4.0–5.0.

Data filtering for removal of low-abundance peaks was performed, requiring a signal–noise ratio of three and a minimum peak height of 10^6^ in at least one sample; filtering resulted in exclusion of three PFAS (PFMOAA, PFBA, PFECA-F), all of which were early-eluting compounds with peak shape broadening which contributed to low peak height.

Several outlier points were identified in the NTA workflow by visually apparent deviations from linearity in calibration curves. Upon manual inspection, most of these resulted from auto-integration errors in Compound Discoverer. The areas for these data points were therefore substituted with manually integrated peak areas obtained with 6 ppm mass tolerance, as in the targeted method. Outlier points that could not be resolved via manual peak integration were removed from the final data set. Details of the excluded data points are available in Supporting Information 6.0. The level of manual inspection and adjustment performed is expected to be the same as that typically used during NTA data processing.

### Data analysis

#### Traditional quantification with calibration curves

Ion abundances for each compound and IS were exported from Xcalibur and Compound Discoverer. Abundances and analyte concentrations were log-transformed prior to generation of calibration curves (logarithm base-2 was used to correspond to the twofold dilution series of the prepared solutions). Calibration curves were created in R version 4.1.0 with regression models and 95% prediction bands fitted to the data using the R function *lm* from the package *stats* [24]. To visualize the calibration curves and their associated prediction bands, the R package *ggplot2* [25] and the function *predict* from the package *stats* were used.

Data quality was assessed using the slope and *R*^2^ values of the fitted calibration curves. A slope of 1.0 indicates that a change in concentration results in a perfectly proportional change in observed ion abundance. Slopes significantly different from 1.0 can suggest that RF is concentration dependent, possibly indicating detector saturation, limit-of-detection based censoring, or other non-linear phenomena.

The R package *investr* [26] with the function *calibrate* was used to obtain inverse estimates based on the calibration curves. For each examined value of *Y* (i.e., an observed ion abundance), inverse estimates included a best estimate of concentration ($\widehat{Conc}$), as well as a lower and upper confidence limit (${\widehat{Conc}}_{LCL}$ and ${\widehat{Conc}}_{UCL}$, respectively), derived from the 95% prediction bands. To assess the performance of the models (with performance metrics described in the “Accuracy, uncertainty, and reliability metrics” section), we used leave-one-out cross-validation (LOO-CV). This method removes a data point from a set of calibration points and then uses the remaining calibration points to fit a calibration curve and predict the concentration of the removed data point. This procedure continues until all data points have served as the removed data point.

For Approach 1 (A1; see Fig. 1), inverse estimates were based on data from the targeted analysis workflow, with *Y* values being normalized ion abundance using matched internal standards (A1 therefore represents an internal standard method). For Approach 2 (A2), inverse estimates were again based on data from the targeted analysis workflow, but without internal standards normalization (A2 therefore represents an external standard method). For Approach 3 (A3), inverse estimates were based on the abundance from the NTA workflow without IS normalization.

#### Quantitative non-targeted analysis

Groff et al. (2022) [16] presented a bounded RF method as a naïve approach to qNTA in the absence of confident structural information. Using the bounded RF method, concentration estimates with associated inverse confidence intervals are predicted using RF percentile estimates from a population of surrogate calibrant training data. The RF percentiles are estimated using a non-parametric bootstrap procedure to account for potential non-normality of the sampled RF distribution; the non-parametric bootstrap procedure makes no assumptions of the shape of the underlying population distribution. The median RF estimate (${\widehat{RF}}_{0.50}$) is used to estimate the concentration of any analyte of interest. While the median RF estimate may not best approximate the true RF of the chemical(s) of interest, it represents the central tendency from the chemical training set and is thus equally likely to over- or under-estimate the true RF value(s). All point concentration estimates are accompanied by lower and upper statistical limits at a given confidence level (i.e., $100\times \left[1-\alpha \right]\%$) using the $\frac{\alpha }{2}$ and $1-\left(\frac{\alpha }{2}\right)$ percentile RF estimates from the training set, where *α* is the defined significance level, set here to 0.05 for a 95% confidence interval. These estimates reflect the distribution of RFs across the surrogate chemicals and thus express the uncertainty of the inverse concentration estimates.

The set of chemicals observable through a specific NTA workflow, which we term the “chemical space,” differs for each experiment depending on the selected matrix, extraction method, ionization condition, and detector (amongst other variables) [27]. These experimental factors additionally influence the RFs observed for chemicals across different experiments, which we term the “RF space.” In order to provide reliable confidence intervals for concentration estimates, the surrogate calibrant training data should be selected to represent the RF range of the analytes of interest. The use of large “global” surrogate chemical RF space is appropriate for observed analytes that lack confident structural identifications (e.g., Schymanski et al. level 3 identifications) [28] but results in predictions with high uncertainty; previous implementation of this approach with a surrogate chemical RF space of 200 + chemicals resulted in confidence intervals spanning three orders of magnitude [16]. When confident structural information is available for detected analytes (e.g., Schymanski et al. level 2a identifications), reducing the surrogate chemical RF space should reduce the uncertainty of the qNTA predictions. In this case, rather than all available surrogates, the training data would consist of a subset of surrogates that are predicted or expected to behave similarly to the analyte of interest. Ideally, this would minimize the size of the prediction confidence interval while retaining predictive accuracy. We tested this “expert-selected surrogates” approach (Approach 4 [A4]) using a subset of three expert-selected surrogates for each analyte (Fig. 1). Surrogates were selected as the three closest eluting compounds to the analyte of interest that shared the primary ionizing group (e.g., carboxylic acid, sulfonic acid). When fewer than three compounds shared the primary ionizing group of the analyte of interest, compounds with the same chain length as the analyte of interest were used as the additional surrogates. The list of chosen surrogates for each compound can be found in Table S2.

The “global surrogate” bounded RF approach (Approach 5 [A5]) was also evaluated, using all examined PFAS, excluding the chemical of interest, as the surrogate population. This approach is expected to provide estimates with the greatest uncertainty of all five quantitation approaches due to the wide range of utilized surrogate training data. However, it represents the most generalizable qNTA approach as it requires no structural information about the analyte of interest. For the current work, A4 and A5 were evaluated using a confidence level of 0.95.

In this study, the empirical RF data for each chemical were obtained by dividing the ion abundance of each calibration point by its true concentration. Bootstrap resampling (using custom R functions) was performed on the surrogate calibrant RFs to generate median estimates of the 2.5th, 50th, and 97.5th percentile RF values for the defined chemical space (which was different for each PFAS analyte and approach (i.e., A4 vs. A5)). The bootstrap sampling used a hierarchical sampling method, with random selection of one chemical from the surrogate space and then random selection of one RF for the selected chemical. The hierarchical sampling method allows each chemical to have an equal probability of selection without influence from the number of RF measurements it has. Sampling was performed with replacement until the resampled set was equal in size to the total number of chemicals in the experiment (*n* = 26 for A4 and A5). The estimated 2.5th, 50th, and 97.5th percentile RF values (abbreviated as ${\widehat{RF}}_{0.025}$, ${\widehat{RF}}_{0.50}$, and ${\widehat{RF}}_{0.975}$, respectively) were calculated for each sampled set. Bootstrap resampling was performed for 10,000 replicates, and the medians of the ${\widehat{RF}}_{0.025}$, ${\widehat{RF}}_{0.50}$, and ${\widehat{RF}}_{0.975}$ values were then calculated across all replicates.

For A4 and A5, the bootstrap median ${\widehat{RF}}_{0.975}$, ${\widehat{RF}}_{0.50}$, and ${\widehat{RF}}_{0.025}$ values were used to calculate ${\widehat{Conc}}_{LCL}$ (the lower confidence limit estimate; Eq. 1a), $\widehat{Conc}$ (the point concentration estimate; Eq. 1b), and ${\widehat{Conc}}_{UCL}$ (the upper confidence limit estimate; Eq. 1c), respectively, for each observed ion abundance (abbreviated “$Abun$”) of each PFAS analyte:

1a $${\widehat{\mathrm{Conc}}}_{\mathrm{LCL}}=\frac{\mathrm{Abun}}{{\widehat{\mathrm{RF}}}_{0.975}}$$

1b $$\widehat{\mathrm{Conc}}=\frac{\mathrm{Abun}}{{\widehat{\mathrm{RF}}}_{0.50}}$$

1c $${\widehat{\mathrm{Conc}}}_{\mathrm{UCL}}=\frac{\mathrm{Abun}}{{\widehat{\mathrm{RF}}}_{0.025}}$$

These equations are generalizable to different confidence levels (i.e., 1-*α*) with the estimates of the lower (Eq. 1a) and upper confidence limits (Eq. 1c) calculated using ${\widehat{RF}}_{1-\left(\frac{\alpha }{2}\right)}$ and ${\widehat{RF}}_{\frac{\alpha }{2}}$, respectively.

### Comparison of traditional and quantitative non-targeted analysis approaches

#### Accuracy, uncertainty, and reliability metrics

**Fig. 2:**
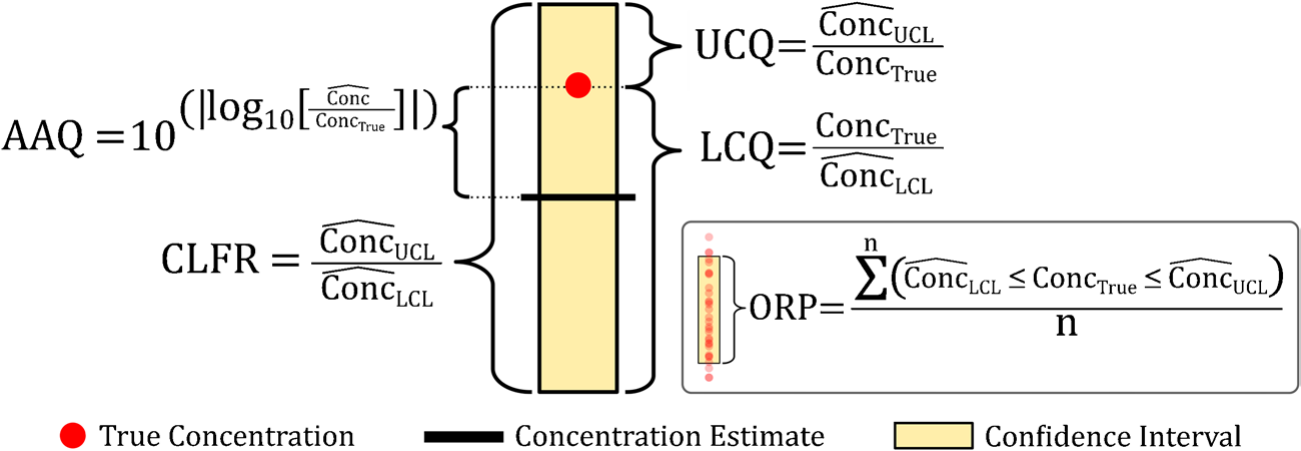
Diagram of selected accuracy, uncertainty, and reliability metrics (*AAQ, absolute accuracy quotient; CLFR, confidence limit fold range*; *UCQ, upper confidence quotient*; *LCQ, lower confidence quotient;* and *ORP, overall reliability percentage*)

All five approaches yielded a point concentration estimate (i.e., $\widehat{Conc}$) and 95% confidence limit estimates (i.e., ${\widehat{Conc}}_{LCL}$ and ${\widehat{Conc}}_{UCL}$) for each observed ion abundance of each PFAS analyte. Approach performance was evaluated using these values along with the true concentration associated with each observation (${Conc}_{True}$). Specifically, performance was assessed using newly defined metrics that communicate predictive accuracy, uncertainty, and reliability (Fig. 2).

##### *Accuracy*

The accuracy of each approach is a measure of the similarity between $\widehat{Conc}$ and ${Conc}_{True}$. The absolute relative error (*ARE*, calculated as $\left(\left|\widehat{Conc}-{Conc}_{True}\right|/{Conc}_{True}\right)\times 100$) is commonly reported for targeted applications to communicate accuracy. While this metric is appropriate for targeted work, the use of *ARE* for qNTA applications can fail to properly capture the magnitude of the differences between $\widehat{Conc}$ and ${Conc}_{True}$. Specifically, severe underestimates can yield *ARE* values no larger than 100%, while severe overestimates can yield *ARE* values that are unbounded. A detailed description of the limitations of *ARE* as a qNTA accuracy metric is available in Supporting Information 7.0.

For the current work, we convey method accuracy based on the quotient of $\widehat{Conc}$ and ${Conc}_{True}$, which we term the *Accuracy Quotient* (*AQ*, Eq. 2):

2 $$\mathrm{Accuracy}\;\mathrm{Quotient}\;(\mathrm{AQ})=\frac{\widehat{\mathrm{Conc}}}{{\mathrm{Conc}}_{\mathrm{True}}}$$

This metric conveys both the magnitude and direction of prediction error, where 0 < *AQ* ≤ 1 when $\widehat{Conc}$ ≤ ${Conc}_{True}$ and *AQ* > 1 when $\widehat{Conc}$ > ${Conc}_{True}$. Importantly, for any given qNTA study, if the general magnitude of underprediction matches the magnitude of overprediction, then the central tendency of *AQ* values will be ~ 1. Thus, the reporting of *AQ* summary statistics may lead to misinterpretation. An alternative to the *AQ* is the *Absolute Accuracy Quotient* (*AAQ*), which conveys the fold-change magnitude but not the direction of qNTA prediction error (in other words, *AAQ* is always ≥ 1). The *AAQ* is calculated using the absolute value of the logged quotient of $\widehat{Conc}$ and ${Conc}_{True}$, prior to exponentiation into rectilinear space (Eq. 3). This metric is mathematically equivalent to the “prediction error” reported by Liigand et al. for similar applications [20]. We introduce a new term here to clearly differentiate the accuracy metric from other metrics used to communicate uncertainty and reliability, which are described below.

3 $$\mathrm{Absolute}\;\mathrm{Accuracy}\;\mathrm{Quotient}\;\left(\mathrm{AAQ}\right)=10^{\left|\log_{10}\left(\frac{\widehat{\mathrm{Conc}}}{{\mathrm{Conc}}_{\mathrm{True}}}\right)\right|}$$

##### *Uncertainty*

The uncertainty of each approach was assessed using metrics derived from upper and lower confidence limit estimates. The first metric, termed the *Confidence Limit Fold Range* (*CLFR*), quantifies the uncertainty as the quotient of any paired upper and lower confidence limit estimates (Eq. 4):

4 $$\mathrm{Confidence}\;\mathrm{Limit}\;\mathrm{Fold}\;\mathrm{Range}\;\left(\mathrm{CLFR}\right)=\frac{{\widehat{\mathrm{Conc}}}_{\mathrm{UCL}}}{{\widehat{\mathrm{Conc}}}_{\mathrm{LCL}}}$$

The second metric, termed the *Confidence Quotient*, quantifies the fold difference between the estimated upper or lower confidence limit and the true concentration. Specifically, the *Upper Confidence Quotient (UCQ)* quantifies the fold difference between ${\widehat{Conc}}_{UCL}$ and ${Conc}_{True}$ (Eq. 5a), and the *Lower Confidence Quotient (LCQ)* quantifies the fold difference between ${Conc}_{True}$ and ${\widehat{Conc}}_{LCL}$ (Eq. 5b). Using these two equations, anytime *UCQ* or *LCQ* is less than unity, the true concentration lies outside the estimated confidence interval:

5a $$\mathrm{Upper}\;\mathrm{Confidence}\;\mathrm{Quotient}\;\left(\mathrm{UCQ}\right)=\frac{{\widehat{\mathrm{Conc}}}_{\mathrm{UCL}}}{{\mathrm{Conc}}_{\mathrm{True}}}$$

5b $$\mathrm{Lower}\;\mathrm{Confidence}\;\mathrm{Quotient}\;\left(\mathrm{LCQ}\right)=\frac{{\mathrm{Conc}}_{\mathrm{True}}}{{\widehat{\mathrm{Conc}}}_{\mathrm{LCL}}}$$

*UCQ* and *LCQ* may be considered in conjunction or focus may be placed on only one metric, depending on the objectives of the study. Here we focus on *UCQ* as the primary use case in order to examine the protectiveness of upper confidence limit estimates, consistent with use in risk-based evaluations [8]. Focus on *LCQ* is prudent when overprediction, not underprediction, is the main concern. Here we consider this focus as the secondary use case, with a full description provided in Supporting Information 8.0.

The use of *UCQ* in the current study directly corresponds to the use of the “error quotient” metric described in Groff et al. 2022 [16]; a new term is provided here for improved clarity across qNTA performance metrics (Eqs. 1–6c). Values of $UCQ<1$ indicate failure of the estimated upper confidence limit to meet or exceed the true concentration, with the magnitude of *UCQ* decreasing as the severity of the underprediction increases. Values of $UCQ>1$ indicate that the estimated upper confidence limit exceeds the true concentration, with the magnitude of *UCQ* increasing as the severity of overprediction increases.

##### *Reliability*

The reliability of a confidence interval estimation approach can be described based on the percentage of true concentration values that are (1) at or below the upper confidence limit estimate (defined as the *Upper Reliability Percentage* (*URP*); Eq. 6a); (2) at or above the lower confidence limit estimate (defined as the *Lower Reliability Percentage* (*LRP*); Eq. 6b); (3) or within the estimated confidence interval (defined as *Overall Reliability Percentage* (*ORP*); Eq. 6c).

For a confidence level of 0.95, we expect $URP\approx 97.5\%$, $LRP\approx 97.5\%$, and $ORP\approx 95\%$. The *ORP* is considered a general measure of reliability and should always be reported. Both the *URP* and *LRP* can be additionally reported, or focus can be placed on either *URP* (primary use case) or *LRP* (secondary use case), depending on whether upper or lower confidence limit reliability is of concern. The primary use case (using *URP*) is the focus of the current analysis, with a description of the secondary use case (using *LRP*) available in Supporting Information 8.0:

6a $$\mathrm{Upper}\;\mathrm{Reliability}\;\mathrm{Percentage}\;\left(\mathrm{URP}\right)=\frac{\sum_{\mathrm i=1}^{\mathrm n}({\mathrm{Conc}}_{{\mathrm{True}}_{\mathrm i}}\leq{\widehat{\mathrm{Conc}}}_{{\mathrm{UCL}}_{\mathrm i}})}{\mathrm n}\ast100$$

6b $$\mathrm{Lower}\;\mathrm{Reliability}\;\mathrm{Percentage}\;\left(\mathrm{LRP}\right)=\frac{\sum_{\mathrm i=1}^{\mathrm n}({\mathrm{Conc}}_{{\mathrm{True}}_{\mathrm i}}\geq{\widehat{\mathrm{Conc}}}_{{\mathrm{LCL}}_{\mathrm i}})}{\mathrm n}\ast100$$

6c $$\mathrm{Overall}\;\mathrm{Reliability}\;\mathrm{Percentage}\;\left(\mathrm{ORP}\right)=\frac{\sum_{\mathrm i=1}^{\mathrm n}({\widehat{\mathrm{Conc}}}_{{\mathrm{LCL}}_{\mathrm i}}\leq{\mathrm{Conc}}_{{\mathrm{True}}_{\mathrm i}}\leq{\widehat{\mathrm{Conc}}}_{{\mathrm{UCL}}_{\mathrm i}})}{\mathrm n}\ast100$$

#### Statistical comparison of quantitative approaches

Statistical comparison of performance across approaches was based on pairwise differences in log-transformed accuracy and uncertainty metrics (reliability metrics were not considered in statistical comparisons as only one *URP*, *LRP*, and *ORP* value is reported per approach). Specifically, one-way random effects models were used to facilitate pairwise comparisons of log-transformed *AAQ*, *CLFR*, *UCQ*, and *LCQ* values (Eq. 7). Random effects models were required due to the non-independent nature of the experimental data for each PFAS.

7 $${Y}_{ij}=\mu +{b}_{i}+{\varepsilon }_{ij}$$

In Eq. 7, ${Y}_{ij}$ represents the difference in log_10_-transformed *AAQ*, *CLFR*, *UCQ*, or *LCQ* values, between two selected approaches (e.g., A2 and A1), corresponding to the *j*^*th*^ ion abundance of the *i*^*th*^ PFAS analyte; *μ* represents the fixed global mean; *b*_*i*_ represents the random effect for the *i*^*th*^ PFAS analyte; and ε_*ij*_ represents the random error for the *j*^*th*^ value of the *i*^*th*^ PFAS analyte. It is assumed that *b*_*i*_ and *ε*_*ij*_ are independent random variables and that *b*_*i*_ ∼ N (0, ${\sigma }_{b}^{2}$) and ε_*ij*_ ∼ N (0, ${\sigma }_{w}^{2}$). A compound symmetry covariance matrix was used for Eq. 7, and model assumptions (e.g., normality and homoscedasticity) were examined by visual inspection of conditional Pearson residuals. For ease of interpretation, models for *UCQ* and *LCQ* did not consider any individual values of *UCQ* or *LCQ* that were less than unity (reflecting scenarios where ${\widehat{Conc}}_{UCL}$ underestimated ${Conc}_{True}$ or ${\widehat{Conc}}_{LCL}$ overestimated ${Conc}_{True}$).

Results of Eq. 7 indicate whether one inverse estimation approach yielded significantly larger ${log}_{10}\left(AAQ\right)$, ${log}_{10}\left(CLFR\right)$, ${log}_{10}\left(UCQ\right)$, or ${log}_{10}\left(LCQ\right)$ values than another (where ${H}_{0}: \mu =0;$ ${H}_{1}: \mu \ne 0;$ and α = 0.05). The estimate of $\mu$ (denoted $\widehat{\mu }$) from Eq. 7 is based on the difference in log-transformed metrics; for interpretation in rectilinear space, we consider ${10}^{\widehat{\mu }}$, which approximates the geometric mean ratio of any selected metric (e.g., *AAQ*) between any two selected approaches (e.g., A2 vs. A1). The function *lmer* from the R package *lme4* [29] was used to create the one-way random effects models, which were fit based on minimizing the restricted maximum likelihood (REML) criterion using the penalized least squares algorithm as implemented in the Eigen templated C++ package. The R package *lmerTest* [30] was used to obtain *p*-values for the fixed global mean effect using the Satterthwaite’s degrees of freedom method.

When comparing performance across A4 and A5, all pairwise differences of ${log}_{10}\left(CLFR\right)$, ${log}_{10}\left(UCQ\right)$, and ${log}_{10}\left(LCQ\right)$ are equivalent for all *j* abundance measurements of each PFAS analyte. In light of this condition (which results from the per-chemical approach to the bootstrap estimation procedures), the median PFAS-specific values of ${log}_{10}\left(CLFR\right)$, ${log}_{10}\left(UCQ\right)$, and ${log}_{10}\left(LCQ\right)$ were considered when comparing performance across A4 and A5. Given a lack of repeat measures, and non-normal distributions of pairwise differences of median values, the Wilcoxon signed-rank test was used in place of the one-way random effects model. Two-sided Wilcoxon signed-rank tests of pairwise differences were performed using the function *wilcox.test* from the *stats* package in R, with significance threshold of α = 0.05. For consistency, the Wilcoxon signed-rank test was implemented for all other approach comparisons (e.g., A2 vs. A1 for *AAQ*) using the median performance metric value for each PFAS analyte.

## Results

### Calibration curves for traditional quantitation approaches

The slopes of the calibration curves for A1 (*n* = 20), A2 (*n* = 29), and A3 (*n* = 26) were used to evaluate the linearity of the abundance vs. concentration relationships. The mean slope estimates from each approach (A1 = 1.00; A2 = 1.00; A3 = 0.973) were statistically indistinguishable from unity (one-sample *t*-tests, *p*_A1_ = 0.732; *p*_A2_ = 0.940, *p*_A3_ = 0.370). Slope estimates from A1 ranged from 0.899 to 1.16, slope estimates from A2 ranged from 0.771 to 1.89, and slope estimates from A3 ranged from 0.800 to 1.40 (Figure S1 and Supplementary File 1). Slopes which are further from unity indicate more intra-chemical RF variation across the tested concentration range, which may result in lower accuracy and reliability metrics and higher uncertainty. Overall, slopes from A2 and A3 spanned a wider range than those from A1. The slopes furthest from 1 using A2 were observed for PFECA-F (slope = 1.41), PFPeS (slope = 1.51), and PFMOAA (slope = 1.89), and the slopes furthest from 1 using A3 were observed for HFPODA (slope = 1.34) and PFHxA (slope = 1.40). Both PFECA-F and PFMOAA were early-eluting compounds that displayed some non-Gaussian chromatographic peak shapes, which may partially explain non-ideal behavior. Peak shape was not part of the quality criteria for this evaluation, and data for these compounds were therefore not removed from consideration. An examination of the relationship between calibration curve metrics (slope and *R*^2^) and the newly defined quantitative performance metrics is provided in the “Drivers of performance across estimation approaches” section.

The *R*^2^ parameter was used to evaluate the goodness of fit of each calibration curve. The mean *R*^2^ from each approach was > 0.970 (A1 = 0.996; A2 = 0.975; A3 = 0.971). The *R*^2^ values from A1 ranged from 0.989 to 0.998, the *R*^2^ values from A2 ranged from 0.925 to 0.989, and the *R*^2^ values from A3 ranged from 0.924 to 0.989 (Figure S1 and Supplementary File 1). Values of *R*^2^ from A2 and A3 were generally lower than those from A1. The lowest *R*^2^ values from A2 were observed for PFBA (*R*^2^ = 0.925), PFPeS (*R*^2^ = 0.947), and PFMOAA (*R*^2^ = 0.960), and the lowest *R*^2^ values from A3 were observed for PFDoDA (*R*^2^ = 0.924), PFTeDA (*R*^2^ = 0.927), and PFPeS (*R*^2^ = 0.929). Of note, A2 and A3 *R*^2^ values were lower than the calibration curve linearity criterion of ~ 0.980 for many compounds due to mild calibration curve roll-off (high concentrations outside the linear dynamic range and having lowered response factors), which is normalized by internal standards in A1. This pattern was not deemed to have enough of an impact on the general linearity of the curves to necessitate narrowing the calibration range.

### Response factor distributions for qNTA approaches

**Fig. 3:**
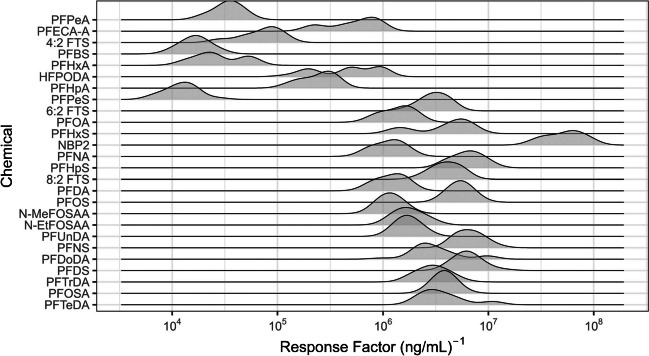
Response factor (RF) distributions, per chemical (*n* = 26), for NTA data (used for A3–A5). Chemicals are ordered from top to bottom based on increasing retention time

Response factors, while theoretically stable across the linear dynamic range, will exhibit intra-chemical variation in their empirical values due to random and systematic experimental error(s). Intra- and inter-chemical variations in RF values of the NTA data (used for A3–A5) were examined using a ridgeline plot generated using the package *ggridges* [31] (Fig. 3; see Figure S2 for representation of targeted RF data). Individual chemical RF distributions were observed to follow an approximate log-normal distribution, with limited instances of skew and multimodality. Inter-chemical variation was of a substantially higher magnitude than intra-chemical variation, with PFAS-specific RF medians spanning an approximate 5000-fold range and intra-chemical variation exhibiting an approximate fourfold range. The total range of RF values was greater than four orders of magnitude. Interestingly, compounds with retention times (RT) < 9 min in our dataset generally appeared to have lower RF values (typically < 10^6^) compared to compounds with RT > 9 min (RF typically > 10^6^), though it is unclear whether this is a solvent composition-related effect or a function of physicochemical properties. Overall, as expected, these results indicate small variation in RF values within chemicals and much larger variation in RF values between chemicals. Several compounds (i.e., Nafion byproduct 2 [NBP2] and PFPeS) were observed to have extreme RF values when compared to the majority of studied PFAS analytes. These compounds, particularly NBP2, have limited RF distributional overlap with the remaining analyte population. Response factor values for these compounds are therefore expected to be poorly represented by leave-one-out bootstrap estimates (e.g., ${\widehat{RF}}_{0.025}$ and ${\widehat{RF}}_{0.975}$) and contribute to poorer performance characteristics (regarding accuracy, uncertainty, and reliability). The relationship between RF distributions and performance metrics is examined further in the “Drivers of performance across estimation approaches” section.

### Comparison of traditional and qNTA approaches

#### Accuracy

##### *Accuracy quotients (AQs)*

Each *AQ* value (Eq. 2) represents the fold difference between a predicted and a true concentration, with an underestimation yielding an *AQ* < 1 and an overestimation yielding an *AQ* > 1. Accuracy quotient values ranged from 0.667 to 1.58 for A1 (median = 0.984), 0.356 to 2.90 for A2 (median = 1.00), 0.295 to 3.61 for A3 (median = 0.982), 0.00163 to 23.0 for A4 (median = 1.01), and 0.00346 to 58.0 for A5 (median = 1.06). For targeted analyses, the level of maximum acceptable error is defined for each method. EPA Method 533 for targeted analysis of PFAS using LC-MS indicates 30% relative error for a point estimate as an acceptable maximum [9]. This corresponds to *AQ* values in the range of 0.700 to 1.30. In the current work, the percentage of *AQ* values within this range for A1, A2, A3, A4, and A5 was 97%, 71%, 68%, 33%, and 15%, respectively. In other words (as examples), 97% of the A1 estimates had no more than 30% relative error, and 15% of the A5 estimates had no more than 30% relative error.

Median *AQ* values very near 1.00 for all approaches indicate limited bias in the direction of estimation error. In other words, there are similar numbers of under- and overestimations such that the 50th percentile *AQ* values are all near unity. Yet, some asymmetry in *AQ* for A4 and A5 (Figure S3) indicates bias in error magnitude towards underestimation. In other words, for A4 and A5, underestimations can be more severe than overestimations. Generally, asymmetrical *AQ* values are expected anytime a skewed RF distribution is the basis for qNTA predictions. Here, extreme underpredictions with A4 and A5 occurred when ${\widehat{RF}}_{0.50}$ for a given analyte was much larger than that analyte’s median RF value. Examination of analyte RF distributions (Fig. 3) reveals several compounds with extremely low RFs relative to the overall RF distribution. The implications of the severity of under- vs. overestimation should always be considered in the context of a given study’s objective(s). For applications towards chemical risk assessment, the risk of enhanced underestimation warrants considerable discussion and underscores the need for confidence interval estimation and interpretation (described in the “Uncertainty” and “Reliability” sections).

##### *Absolute accuracy quotients (AAQs)*

**Fig. 4:**
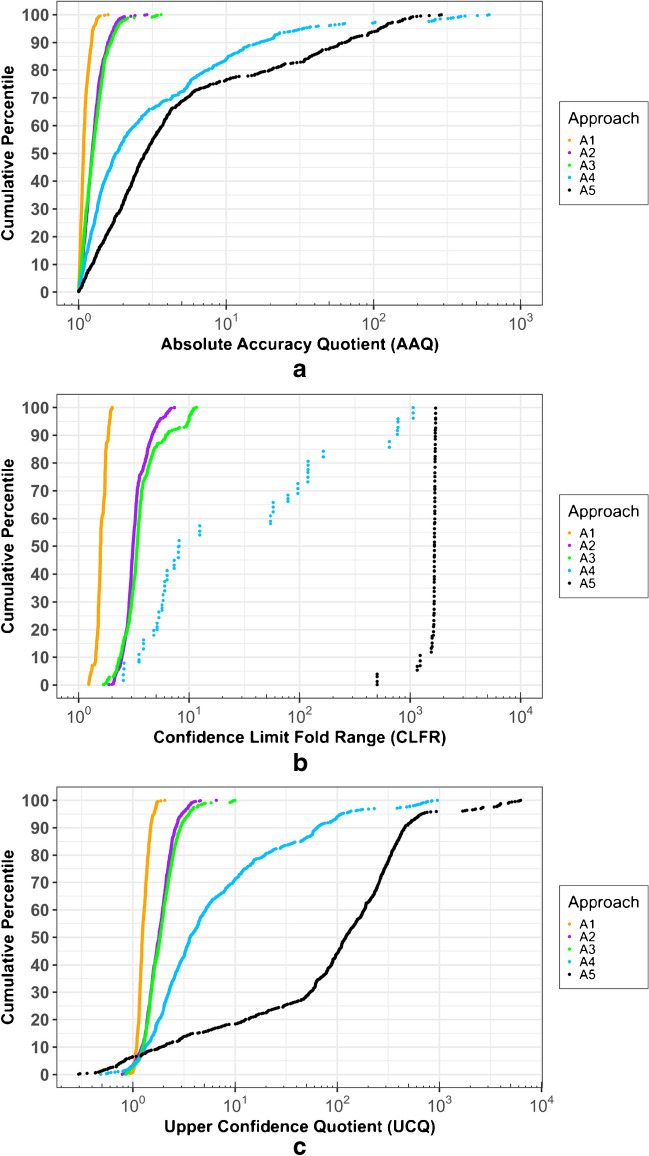
Cumulative percentile distributions of absolute accuracy quotient (*AAQ*; **a**), confidence limit fold range (*CLFR*; **b**), and upper confidence quotient (*UCQ*; **c**) for each approach

Each *AAQ* value (Eq. 3) represents the fold difference between a predicted and a true concentration, with both under- and overestimations yielding *AAQ* values > 1. Individual *AAQ* values across all approaches ranged from 1.00 to 612 (Fig. 4a and Table S3). This means that in one extreme case, a true value was (nearly) perfectly predicted, and in the other extreme case, the concentration estimate deviated from the true value by a factor of 612. Median *AAQ* values for A1, A2, A3, A4, and A5 were 1.08, 1.23, 1.24, 1.80, and 2.85, respectively (Table S4), and corresponding ninety-fifth percentile *AAQ* values were 1.26, 1.73, 1.80, 33.5, and 111.

Pairwise comparisons, via one-way random effects models and Wilcoxon signed-rank tests (results given in Table 1 and Supporting Information 9.0, respectively), were used to test for differences in log-transformed *AAQ* values between approaches. Overall, the largest *AAQ* differences were observed when comparing A1 and A5, with A5 values approximately 4 × larger than paired A1 values, on average. Significant differences in log-transformed *AAQ* values were observed for all pairwise comparisons except A3 vs. A2 (Table 1 and Supporting Information 9.0). The lack of a significant difference between A2 and A3 suggests that the use of NTA acquisition parameters (A3) instead of targeted acquisition parameters (A2) did not appreciably impact predictive accuracy. It is noteworthy that only a modest difference in *AAQ* was observed between A4 and A5 (*p* = 0.05; ${10}^{\widehat{\mu }}=1.56$). This reflects a somewhat marginal improvement in accuracy when using expert-selected surrogates (A4) over global surrogates (A5). Importantly, observed differences in *AAQ* values between A4 and A5 may vary given a new set of analytes, global surrogates, and expert-selected surrogates. Thus, this result is not necessarily extensible to future applications.

**Table 1:** Results of pairwise statistical comparisons of log-transformed *AAQ*, *CLFR*, *UCQ*, and *LCQ* values for the five quantitation approaches. Pairwise fold differences greater than one indicate decreased performance (lower accuracy or higher uncertainty) observed for the first listed approach in the pair. Non-parametric pairwise comparisons are available in SI 9.0

	Absolute accuracy quotient (*AAQ*)			Confidence limit fold range (*CLFR*)			Upper confidence quotient (*UCQ*)			Lower confidence quotient (*LCQ*)		
Approach comparison	${10}^{\widehat{\mu }}$(~ GM^a^ fold difference)	*p*-value	*n*^c^	${10}^{\widehat{\mu }}$(~ GM fold difference)	p-value	*n*	${10}^{\widehat{\mu }}$(~ GM fold difference)	*p*-value	*n*	${10}^{\widehat{\mu }}$(~ GM fold difference)	*p*-value	*n*
A2 vs. A1	1.14	***p* < 0.001**	510	1.94	***p* < 0.001**	510	1.42	***p* < 0.001**	489	1.38	***p* < 0.001**	484
A3 vs. A1	1.17	***p* < 0.001**	483	2.17	***p* < 0.001**	483	1.49	***p* < 0.001**	465	1.49	***p* < 0.001**	453
A4 vs. A1	2.47	***p* < 0.001**	483	19.8	***p* < 0.001**	483	4.93	***p* < 0.001**	466	4.30	***p* = 0.001**	422
A5 vs. A1	3.96	***p* < 0.001**	483	1030	***p* < 0.001**	483	40.5	***p* < 0.001**	456	25.9	***p* < 0.001**	460
A3 vs. A2	1.02	*p* = 0.300	663	1.12	*p* = 0.192	663	1.05	*p* = 0.290	636	1.08	*p* = 0.117	651
A4 vs. A2	2.57	***p* = 0.001**	663	7.84	***p* < 0.001**	663	3.20	***p* < 0.001**	620	2.90	***p* = 0.007**	582
A5 vs. A2	4.02	***p* < 0.001**	663	486	***p* < 0.001**	663	36.5	***p* < 0.001**	600	16.4	***p* < 0.001**	634
A4 vs. A3	2.53	***p* = 0.002**	663	7.02	***p* < 0.001**	663	3.03	***p* < 0.001**	624	2.70	***p* = 0.015**	581
A5 vs. A3	3.96	***p* < 0.001**	663	435	***p* < 0.001**	663	35.1	***p* < 0.001**	603	15.4	***p* < 0.001**	624
A5 vs. A4^b^	1.56	***p* = 0.050**	663	69.3	***p* < 0.001**	26	12.1	***p* < 0.001**	24	5.67	***p* < 0.001**	25

*P*-values was assessed using a significance threshold of α = 0.05. Significant values are indicated using bolded text

^a^*GM* geometric mean. The GM fold difference is approximately equal to ${10}^{\widehat{\mu }}$

^b^A5 vs. A4 comparisons of *CLFR*, *UCQ*, and *LCQ* were based on non-parametric tests using PFAS-specific median values. ${10}^{\widehat{\mu }}$values from non-parametric tests indicate fold ranges calculated using pseudo-median $\widehat{\mu }$ values

^c^The “*n*” columns indicate the number of values used for each approach comparison and differ depending on the number of measurements shared across approaches. A measurement could be excluded either due to unavailability in a certain approach (e.g., chemicals without mass-labeled internal standards could not be used for A1) or inability to interpretably compare the log-transformed metric (*UCQ* and *LCQ* values less than unity). The “*n*” for A5 vs. A4 comparisons of *CLFR*, *UCQ*, and *LCQ* reflect the use of per-chemical non-parametric comparisons (metrics summarized as median metric values per chemical)

Chemical-specific median *AAQ* values ranged from 1.05 (N-EtFOSAA) to 1.16 (PFTeDA) for A1, 1.15 (N-MeFOSAA) to 1.39 (PFPeS) for A2, 1.10 (PFOSA) to 1.48 (PFHxS) for A3, 1.16 (PFOS) to 328 (PFPeS) for A4, and 1.16 (PFUnDA) to 155 (PFPeS) for A5 (Figure S4). These results indicate that median *AAQ* values varied little across chemicals for approaches A1–A3 but varied by up to two orders of magnitude for A4 and A5. Per-chemical *AAQ* values for all approaches are provided in Table S5 and visualized as a heatmap in Figure S4a, with further discussion located in the “Drivers of performance across estimation approaches” section.

#### Uncertainty

##### *Confidence limit fold ranges (CLFRs)*

Each *CLFR* value (Eq. 4) represents the fold difference between paired upper and lower confidence limit estimates of chemical concentration. Individual *CLFR* values across all approaches ranged from 1.24 to 1700 (Fig. 4b and Table S3). In other words, the smallest confidence interval spanned a 1.24-fold range (for A1), and the largest spanned a 1700-fold range (for A5). Median *CLFR* values for A1, A2, A3, A4, and A5 were 1.57, 3.09, 3.37, 8.12, and 1660, respectively, and corresponding ninety-fifth percentile values were 1.89, 5.31, 9.93, 776, and 1700.

Inverse confidence limits are seldom reported in targeted studies as measures of uncertainty. Rather, coefficient of variation (*CV*) estimates, based on replicate analyses, are used to communicate reproducibility and measurement precision. While a *CV* value can be used to estimate a concentration confidence interval, this interval is not necessarily equivalent to that derived from inverse estimation using a calibration curve (described in the “Traditional quantification with calibration curves” section) or qNTA methods (described in the section “Quantitative non-targeted analysis”). Nevertheless, for the purpose of translation, we still compare our calculated *CLFR* values from A1 to A5 to a theoretical *CLFR.* Assuming five replicate measures with a maximum acceptable *CV* of 30%, the inverse prediction *CLFR* would be 2.19. The *CLFR* ≤ 2.19 threshold was met for 100% of A1 values, 4.35% of A2 values, 4.98% of A3 values, and 0% of A4 and A5 values. The striking difference between A1 and A2 results highlights the value of IS correction in improving precision in targeted quantitative studies. It should be noted that a *CV* of 30% represents the standard for targeted analysis using normalized ion abundances. Approaches that do not include normalization may have higher measurement variability, and the *CLFR* threshold of 2.19 may not be appropriate for these approaches. Consideration of an appropriate maximum *CV* for calibration curves without normalization is important for subsequent qNTA evaluation.

Results of pairwise comparisons of *CLFR* values were very similar to those of *AAQ* values. Specifically, the smallest differences in *CLFR* values were observed between A2 and A3, and the largest differences were observed between A1 and A5. On average, paired *CLFR* values were 1.12 × larger for A3 vs. A2 and 1030 × larger for A5 vs. A1. Significant differences in log-transformed *CLFR* values were observed for all comparisons except A3 vs. A2, suggesting that the method of data acquisition alone (NTA vs. targeted) has little effect on estimation certainty.

Chemical-specific median *CLFR* values ranged from 1.25 (N-MeFOSAA) to 1.93 (PFOSA) for A1, 2.11 (PFOSA) to 6.42 (PFPeS) for A2, 1.81 (PFHxA) to 10.1 (PFDoDA) for A3, 2.53 (PFDS) to 1060 (PFHxS) for A4, and 500 (NBP2) to 1700 (HFPODA) for A5 (Figure S4). Overall, per-chemical median *CLFR* values differed by less than one order of magnitude for approaches A1–A3, three orders of magnitude for A4, and one order of magnitude for A5.

##### *Confidence quotients (CQs)*

Each *UCQ* value (Eq. 5a) represents the fold difference between a paired upper confidence limit estimate and true concentration. When the upper confidence limit is considered as a protective estimate for provisional risk evaluation (as recommended by [8, 16]), the *UCQ* then quantifies the magnitude of protectiveness as a function of the underlying predictive uncertainty. In other words, greater predictive uncertainty leads to larger *UCQs* and perhaps unreasonably protective estimates. In the current work, *UCQ* values across all approaches ranged from 0.296 to 6250 (Fig. 4c and Table S3); meaning that, in one extreme case, ${\widehat{Conc}}_{UCL}$ was only ~ 30% of ${Conc}_{True}$, and in the other extreme case, ${\widehat{Conc}}_{UCL}$ was more than 6000 × higher than ${Conc}_{True}$. Median *UCQ* values for A1, A2, A3, A4, and A5 were 1.24, 1.79, 1.84, 3.65, and 121, respectively, and corresponding ninety-fifth percentile values were 1.61, 3.00, 3.53, 109, and 669.

There are no existing *UCQ* guidance thresholds that relate to targeted or qNTA studies. Given this lack of guidance, a *UCQ* of 10 may be considered a starting reference threshold for method comparison. The *UCQ* ≤ 10 threshold was met for 100% of A1–A3 values, 71% of A4 values, and 18% of A5 values. A UCQ < 1 indicates an upper confidence limit which underestimates the true concentration, with the severity of underestimation readily conveyed by the magnitude of *UCQ* (i.e., the extent to which *UCQ* is less than 1). For A1, A2, A3, A4, and A5, the most extreme underestimations yielded *UCQ* values of 0.846, 0.789, 0.856, 0.486, and 0.296, respectively. In other words, across all chemicals and approaches, ${\widehat{Conc}}_{UCL}$ never fell below ${Conc}_{True}$ by more than a factor of 3.5.

Results of pairwise comparisons of *UCQ* values were very similar to those of *AAQ* and *CLFR* values. Specifically, the smallest differences in *UCQ* values were observed between A2 and A3, and the largest differences were observed between A1 and A5. On average, paired *UCQ* values were 1.05 × larger for A3 vs. A2 and 40.5 × larger for A5 vs. A1. Significant differences in log-transformed *UCQ* values were observed for all comparisons except A3 vs. A2, again highlighting the lack of difference in performance resulting from NTA vs. targeted acquisition data processing.

Chemical-specific median *UCQ* values (Figure S4) ranged from 1.10 (N-MeFOSAA) to 1.41 (PFOSA) for A1, from 1.49 (N-MeFOSAA) to 2.61 (PFPeS) for A2, from 1.40 (PFHxA) to 3.00 (PFDoDA) for A3, from 1.00 (PFHxA) to 559 (PFHxS) for A4, and from 0.552 (PFPeS) to 3780 (NBP2) for A5. Interestingly, for A5, two chemicals (PFPeS and PFBS) had median *UCQ* values less than 1, indicating that ${\widehat{Conc}}_{UCL}$ for these chemicals generally lay below ${Conc}_{True}$. Overall, per-chemical median *UCQ* values differed by less than threefold for approaches A1–A3, two orders of magnitude for A4, and four orders of magnitude for A5. While these results highlight only *UCQ* values for the primary use case, results for *LCQ* are given in Supporting Information 8.0 to support the secondary use case.

#### Reliability

##### *Reliability percentages (ORP, URP, LRP)*

The *ORP* (Eq. 6c) reflects the percentage of true concentration values that fall at or within the estimated confidence limits. The *ORP* is the best general measure of reliability and is expected to be ~ 95% for each inverse prediction approach, using our defined confidence limits. Similar *ORP* values were observed for A1 (94.3%), A2 (96.3%), and A3 (95.5%) (Table S4). Overall reliability was slightly lower for qNTA approaches, with *ORP* < 90% for both A4 (84.6%) and A5 (89.4%).

Chemical-specific *ORP* values ranged from 88.9 to 100% for A1, 92.3 to 100% for A2, 85.2 to 100% for A3, and 0.00 to 100% for both A4 and A5 (Table S5 and Figure S4). With A1–A3, nearly all true concentration values of each chemical were observed to lie within the estimated confidence intervals. This result is expected when using chemical-specific calibration curves and serves as validation of the utilized inverse prediction methods. With A4, twelve of 26 chemicals had *ORP* values ≥ 95%, ten had *ORP* values ≥ 70% and < 95%, and four had *ORP* values < 70%. With A5, 22 of 26 chemicals had *ORP* values of 100%, and three of the remaining four chemicals had *ORP* values < 15% (meaning that true concentrations of these chemicals were seldom contained within estimated confidence intervals). These results highlight that overall reliability with A4 is strongly influenced by the representativeness of the expert-selected surrogates, whereas A5 reliability is strongly influenced by extreme RF behaviors of few chemicals (i.e., those at the tails of the global RF distribution).

The *URP* (Eq. 6a) reflects the percentage of true concentration values that fall at or below the estimated upper confidence limits. Compared to the other reliability metrics (i.e., *ORP* and *LRP*), the *URP* is best suited for guarding against underestimation by considering the upper confidence limit as a protective estimate. The *URP* is expected to be ~ 97.5% for each inverse prediction approach. The highest *URP* was observed for A1 (99.2%), the lowest *URP* was observed for A5 (93.5%), and very similar *URP* values were observed for A2 (96.6%), A3 (97.3%), and A4 (96.5%) (Table S4).

The *LRP* (Eq. 6b) reflects the percentage of true concentration values that fall at or above the estimated lower confidence limits. This metric is best suited for use cases that wish to guard against concentration overestimation by considering the lower confidence limit as a conservative estimate. The *LRP* is expected to be ~ 97.5% for each inverse prediction approach. With the exception of A4 (where *LRP* = 88.1%), all approaches had *LRP* values of 95% or higher (Table S4). Considering these results alongside those for *URP* and *ORP*, A4 yielded both the lowest overall reliability (*ORP* = 84.6%) and the least consistency between *LRP* (88.1%) and *URP (*96.5%). Lower and upper reliability percentages were much more consistent with A5 (*LRP* = 95.9% vs. *URP* = 93.5%), despite the overall reliability score (*ORP* = 89.4%) being below the expected 95%.

#### Drivers of performance across estimation approaches

**Fig. 5:**
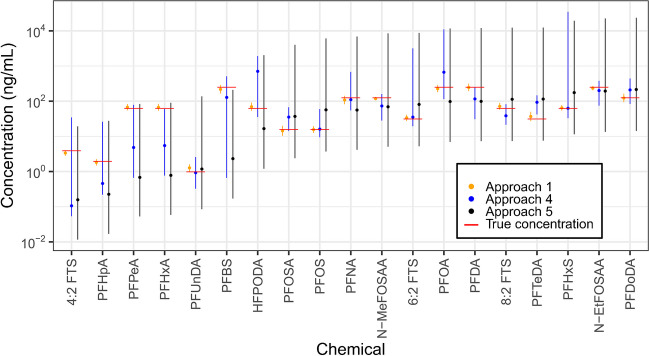
Comparison of single-point estimates with confidence intervals for A1, A4, and A5, visualized using one randomly selected data point per chemical. The true concentration is indicated using a horizontal line across the confidence intervals. Chemicals are organized from left to right in order of increasing A5 ${\widehat{Conc}}_{UCL}$

Comparisons of the five quantitation approaches showed large variations in chemical-specific performance metrics, as described in sections “Accuracy,” “Uncertainty,” and “Reliability.” To help visualize these variations, Fig. 5 shows estimated concentrations and 95% confidence intervals associated with one randomly selected true concentration for each of the 19 chemicals that were commonly assessed across A1 (the benchmark method), A4, and A5. Results for A1 show extremely tight confidence intervals that almost always contain the true values and estimated concentrations that are in extremely close proximity to the true values. Results for A4 show estimated concentrations that can be very near true values (e.g., for PFUnDA) or very distant from true values (e.g., for 4:2 FTS). They also show confidence intervals that are often asymmetric (e.g., for PFBS), highly variable in size across analytes, and not always containing true values (for PFHxA, PFDA, and PFTeDA). Finally, results for A5 show diminished accuracy when compared to those for A4, but confidence intervals that are generally large, uniform, and containing true values for all analytes except PFBS. Taken together, these results highlight (1) sizable performance differences between a gold standard method (A1) and qNTA methods (A4 and A5); (2) considerable variation in uncertainty and diminished reliability when using A4; (3) better accuracy when using A4 vs. A5; and (4) larger uncertainty but better reliability when using A5 vs. A4.

To better understand the factors influencing performance differences, chemical-specific metrics (Table S5) were visualized using heatmaps (Figure S4) and further scrutinized with respect to chemical-specific parameters, such as calibration curve slope and *R*^2^, and surrogate RF values. For A1–A3, chemicals with poorer accuracy and uncertainty metrics (but not reliability) tended to have slopes deviating from 1.0 and lower than average *R*^2^ values (Table S5). Chemicals exemplifying these behaviors include PFTeDA, PFNA, and PFBS for A1; PFPeS, 4:2 FTS, and PFNA for A2; and PFHxS, PFTeDA, and PFECA-A for A3 (see calibration curve plots provided in Supplementary File 1). These general trends highlight the inherent value of robust calibration curves for quantitative applications and reinforce that sub-optimal calibration yields diminished quality of inverse predictions. Improvement of performance metrics for A1–A3 would therefore rely on using calibration curves with high linearity and minimal intra-chemical error. For targeted analysis, this level of control is practical; methods validation can tightly control the linear range of measurements and methods can be optimized for maximum precision. Yet, qNTA requires the extrapolation of calibration data onto novel species, presenting additional quantitative challenges.

For both NTA approaches (i.e., A4 and A5), different factors drove performance for accuracy, uncertainty, and reliability. Accuracy for both approaches was driven by the level of agreement between a chemical’s central tendency RF and the ${\widehat{RF}}_{0.50}$ as calculated for the selected surrogate set (considering a subset of surrogates (A4) or all possible surrogates (A5)). For example, with A4, the minimum chemical-specific *AAQ* was observed for PFOS (*AAQ* = 1.16), and the maximum was observed for PFPeS (*AAQ* = 328). The small *AAQ* for PFOS occurred because the surrogate ${\widehat{RF}}_{0.50}$ value (representing RFs from PFHxS, PFHpS, and PFNS) was very similar to the PFOS central tendency RF. Alternatively, the large *AAQ* value for PFPeS occurred because the surrogate ${\widehat{RF}}_{0.50}$ value (representing RFs from PFBS, PFHxS, and PFHpS) was > 300 × larger than the PFPeS median RF (Table S5 and Fig. 3).

While the variance in surrogate RF values has little bearing on accuracy, it is the primary driver for uncertainty for both A4 and A5. For example, the minimum and maximum chemical-specific *CLFR*s for A4 were observed for PFDS (*CFLR* = 2.53) and PFHxS (*CLFR* = 1060), respectively. As shown in Fig. 3, the RF distributions for PFDS surrogates (PFOS, PFNS, and PFHpS) were very tightly aligned, whereas those for PFHxS surrogates (PFPeS, PFHpS, and PFOS) were very widely scattered. Generally speaking, with A4 and A5, the further the surrogate RF values are distributed in space, the larger the estimated confidence interval and resulting *CLFR*. Surrogate RF variance also affects *UCQ* and *LCQ*, but these measures of uncertainty are additionally affected by the location of ${Conc}_{True}$ relative to the upper and lower confidence limits.

Per-chemical reliability was generally high and stable across chemicals for A1–A3 due to the use of chemical-specific calibration curves (Table S5). For A4 and A5, however, highly uneven reliability was observed across the chemical set, with many chemicals having reliability percentages (*LRP*, *URP*, and *ORP*) of 100% and others having values close to 0% or between 0 and100%. For the qNTA approaches, reliability is affected by both the location and spread of the surrogates RF distribution relative to that of the examined chemical. In other words, reliability is a function of surrogate representativeness. The most extreme example in this dataset is NBP2, which had an *ORP* of 0% for both A4 and A5. Looking at Fig. 3, NBP2 yielded the highest RF values across all analytes. Considering this extreme behavior, neither the three selected surrogates from A4 (PFOS, PFNS, and PFDS) nor the global surrogates from A5 provided adequate coverage of the NBP2 RF distribution.

Another extreme example of reliability is found with PFHxA, which yielded some of the lowest RF values across all analytes. With A4, one of the three surrogates for PFHxA (i.e., PFPeA) was a low responder, contributing to a ${\widehat{RF}}_{0.025}$ value of 24,600, which was nearly identical to the central tendency RF for PFHxA (Table S5). This, in turn, yielded an PFHxA *ORP* of 50.0%. When considering all surrogates with A5, however, ${\widehat{RF}}_{0.025}$ was shifted down to 16,400 (given additional consideration for more extreme surrogates), which produced a PFHxA *ORP* of 83.3%. The opposite scenario occurred for PFPeS, which also yielded some of the lowest RF values in this study. With A4, one of the three surrogates for PFPeS (i.e., PFBS) was a very low responder, contributing to a ${\widehat{RF}}_{0.025}$ value of 11,900, which was very near the central tendency RF for PFPeS (median = 12,300). This, in turn, yielded a PFPeS *ORP* of 55.6%. When considering all surrogates with A5, however, ${\widehat{RF}}_{0.025}$ was shifted up to 22,200, which produced a PFPeS *ORP* of 5.60%.

Regardless of the qNTA approach, certain chemicals will always exhibit extreme behaviors that lead to chemical-specific reliability percentages < 100%. The expectation is that aggregating results across chemicals leads to an overall study reliability near 95%. Moving forward, the goal of any qNTA experimental design is therefore to select representative surrogates with experimental RF values spanning the range of anticipated RF values for new chemicals of interest. It stands to reason that this is more easily accomplished when drawing from a larger pool of representative surrogates. This conjecture is consistent with our results, which generally showed higher and more stable reliability with A5 vs. A4.

## Discussion

This study compared the performance of five quantification approaches using a set of 29 PFAS. The examined approaches spanned those utilizing chemical-specific calibration curves (A1–A3) to those using surrogates for qNTA predictions (A4 and A5). Calibration curve-based approaches were assessed to establish performance benchmarks, examine the importance of internal standard correction, and assess performance differences when using data from targeted vs. non-targeted acquisition methods. Two separate qNTA approaches were assessed to examine the influence of surrogate selection procedures on quantitative performance. While analytical figures of merit (e.g., *ARE* and *CV*) are well-established for traditional targeted methods, they are not necessarily well suited for describing qNTA results (see Supporting Information 7.0). As such, novel performance metrics were needed to facilitate direct comparison between the targeted and qNTA approaches.

The performance metrics developed in the current study communicate the accuracy (i.e., *AQ* and *AAQ*), uncertainty (i.e., *CLFR*, *UCQ*, and *LCQ*), and reliability (i.e., *URP*, *LRP*, and *ORP*) of quantitative analytical estimates. Each metric is relevant to quantitative estimates from both targeted and qNTA applications and designed to capture a wide range of performance across quantification approaches. As utilization of qNTA grows, so too does the need for standardized evaluation methods, reporting metrics, and performance benchmarks [32]. The performance metrics introduced here represent an initial step towards establishing common evaluation criteria for qNTA studies.

Our analysis of more traditional quantification approaches (A1–A3) demonstrated that accuracy and uncertainty (as best characterized by *AAQ* and *CLFR*, respectively) correlate with well-established measures of calibration curve performance, such as curve linearity and goodness of fit (based on *R*^2^). Comparisons across A1, A2, and A3 showed that internal standard normalization is a larger driver of quantitative performance than method of data acquisition (targeted vs. non-targeted), with significantly improved performance metrics observed for A1 compared to A2 and A3, but no significant differences observed between A2 and A3. Comparisons of traditional approaches against qNTA approaches (A4 and A5) demonstrated that, as anticipated, targeted approaches exhibit superior performance with respect to accuracy, uncertainty, and reliability. Finally, comparisons across A4 and A5 showed that using expert-selected surrogates over global surrogates can improve accuracy and uncertainty, but at the cost of reliability.

Multi-aspect assessment of qNTA in terms of accuracy, uncertainty, and reliability is rare in the qNTA literature. Most qNTA studies, to date, have reported only measures of accuracy [8] without associated confidence intervals or measures of reliability. One exception is recent work by Cao et al. [33], which explored using “average calibration curves” to predict concentrations of PFAS. Cao and colleagues reported accuracy and uncertainty metrics for 50 PFAS. Their utilized accuracy metric is analogous to *AQ*, with median values of 1.43 and 0.93 reported for log–log and weighted linear regression models, respectively (calculated using data provided in the Cao et al. Supporting Information [SI]). As described above (see the “Accuracy” section), *AQ* can be misleading if examined in isolation of other performance metrics. Converting the reported results of Cao and colleagues to *AAQ*s yields median values of 2.89 and 2.96 for the two regression approaches. These values are nearly identical to the median *AAQ* of 2.85 observed with A5 in the current study. This result is not surprising given that the “average calibration curve” approach of Cao and the “global surrogates” approach of the current analysis both use all available PFAS calibration data to inform qNTA estimates.

As another recent example of accuracy reporting, Sepman and colleagues [34] assessed their “MS2Quant” model for ionization efficiency prediction using a “prediction error” metric that is mathematically equivalent to *AAQ* (note that the Sepman study predicted ionization efficiencies rather than concentrations — reported prediction errors are still directly comparable to *AAQ*, assuming a linear relationship between ionization efficiency and concentration). Compared to the present study, the experiment of Sepman and colleagues included both a larger and more diverse training set (*n* = 954) and test set (*n* = 239). The reported median prediction error of the test set was 3.2, which is larger than our median *AAQ* values for A4 and A5 (1.80 and 2.85, respectively). Given the more focused chemical space examined in our study compared to that of Sepman, our smaller observed *AAQ*s are expected. Interestingly, the mean prediction error reported by Sepman is several-fold higher than the median prediction error (15.4 compared to 3.2). This aligns with the observed increase in mean *AAQ* over median *AAQ* in our study (mean *AAQ* = 14.7 and 18.7 for A4 and A5, respectively). This difference between mean and median *AAQ*s reflects a right-skewed *AAQ* distribution, which is visible in Fig. 4a. Overall, accuracy metrics reported by Cao et al., Sepman et al., and the current work are all highly similar (within twofold of each other).

As previously mentioned, very few qNTA studies have reported uncertainty and reliability values associated with point concentration estimates. Cao et al. reported upper- and lower-bound limits, defined as “prediction intervals,” associated with PFAS concentration estimates. The reported prediction intervals (which are, to the best of our knowledge, mathematically equivalent to the “inverse confidence intervals” reported here) were described as “quite variable” for log–log models and “more uniform” for weighted linear models. This description reflects mathematical differences, or “ranges,” between the upper- and lower-bound estimates. Across 50 PFAS, the ranges of reported prediction intervals spanned 0.193–917 for the log–log models (with 94.7% reliability, as calculated from SI values) and 107–176 for the weighted linear models (with 98.7% reliability). Since relative error, and not absolute error, is expected to be uniform across the linear dynamic range of any method, one would not expect stability in prediction interval ranges. Rather, one would expect stability in prediction interval fold ranges (or *CLFR*s, using our terminology). The prediction interval fold ranges for the Cao et al. data (calculated using SI values) spanned 154–166 for the log–log models and 16.9–1.75 × 10^6^ for the weighted linear models (the fold range could not be calculated for six values from the weighted linear models due to lower-bound limits being exactly zero). Results for the log–log models are most directly comparable to *CLFR* values reported here, which showed median estimates of 776 and 1700 for A4 and A5, respectively. Interestingly, our *CLFR* values are considerably larger (~ 5 × to ~ 10 ×) than those reported by Cao et al. Based on information provided in the publication, it is difficult to know whether similar inverse estimation techniques were used between studies. This highlights a need to harmonize terminology (e.g., “prediction intervals” vs. “inverse confidence intervals”), metrics, and estimation approaches across studies to allow for the most meaningful comparisons.

Groff et al. represents another qNTA study that reported and validated inverse confidence intervals alongside point concentration estimates (Groff et al. 2022 [16]). In their work, Groff et al. reported uncertainty as an “error quotient” metric that is mathematically equivalent to the *UCQ*. The median error quotient reported by Groff, using an approach analogous to A5, was 10.0 for the ESI- dataset. This is an order of magnitude lower than the A5 median *UCQ* of 121 for the current study. Reliability was reported by Groff and colleagues as the percentage of true concentration values within the estimated 95% confidence intervals, which is equivalent to the *ORP*. The observed containment by confidence interval estimates (94.9%) was very close to the theoretical 95%, which is higher than the *ORP* of 89.4% observed here for A5. It should be noted that the number of data points per chemical was much larger in the current study (*n*≈27) compared to the Groff study (*n*≈3) and that far fewer chemicals were considered here (*n* = 26) than in Groff et al. (*n* = 273). Noting this discrepancy, data from each chemical in the current study had larger contribution to bootstrap statistics and performance metrics.

The uncertainties associated with our qNTA approaches are significantly larger than those associated with targeted approaches (Table 1 and Supporting Information 9.0). Indeed, *CLFR* values associated with A4 and A5 were, on average, ~ 20 × and ~ 1000 × larger than those associated with A1. These performance differences are likely to persist regardless of improvements in qNTA methods due to the use of surrogate data; therefore, when available, targeted approaches will continue to remain the preferred choice for quantitation. However, when targeted approaches cannot be used, qNTA estimates with heightened uncertainty can still inform decisions, as long as the uncertainty can be reliably described. Reliability metrics reported in the current study indicate lower-than-desired reliability for A4 and A5 and high per-chemical variation for A4. Both the process of expert selection and the availability of suitable surrogates certainly influenced the reliability of predictions for individual chemicals. Moving forward, the use of few surrogates for qNTA applications is not likely to capture the inter-chemical variability that drives qNTA reliability, even in the relatively restricted chemical space defined by available PFAS standards.

The challenges of surrogate selection will likely grow as qNTA is applied to more diverse and disparate categories of emerging contaminants. Expert-performed manual surrogate selection is limited by the requirement of analyst knowledge and is inherently subjective. Therefore, although manual surrogate selection is an intuitive choice for domain experts performing qNTA, it is likely unsuitable for widescale adoption. As an alternative, automated surrogate selection based on model predictions provides a strategy to select suitable surrogates in a reproducible fashion. Model predictions guide selection of surrogates with similar predicted ionization efficiencies to the chemicals of interest. To account for unexpected or unknown chemicals detected via NTA, surrogates may be selected to provide broad coverage of the relevant chemical space, in a manner similar to global surrogate use. However, the qNTA estimates for individual chemicals of interest may draw primarily from similar surrogates to provide narrower confidence intervals. For example, in the ionization efficiency estimation approach utilized by Groff et al. [16], confidence intervals of predictions for individual chemicals depend mainly on the variability of similar surrogates but do incorporate some response factor variability from across the range of chosen surrogates. A benefit of this approach is that it can potentially replicate the improved accuracy and smaller uncertainty bounds of expert selection while maintaining the desired reliability. The optimal composition and number of surrogates to use for any given experiment, considering the desired performance and the availability of chemical standards, are an ongoing topic of research.

A limitation of the current investigation was its collection of data from compounds in solvent only. In real-world assessments, chemicals of interest are detected within various matrices, and the levels of chemicals within those matrices ultimately support decisions (e.g., regulation or mitigation). Different matrices such as water, serum, sediment, and other media exert different effects on measured chemical signals [35, 36]. Application of qNTA methods in different matrices is a logical next step of the current work, as it will allow for a quantitative understanding of matrix effects, and further development of qNTA approaches that can be proven accurate, certain, and reliable under any sample conditions.

Given the need for qNTA to address environmental challenges related to emerging contaminants (including PFAS), defensible reporting metrics are necessary. Future qNTA applications should provide metrics to validate the accuracy, uncertainty, and reliability of quantitative predictions. Current practices of using expert-selected surrogate calibrants, or a large population of surrogates, can result in high uncertainty and lower-than-expected reliability. To ensure that reported confidence intervals are sufficiently reliable, qNTA approaches should be evaluated using appropriate test sets to confirm that performance meets or exceeds expectations. Future qNTA studies should therefore incorporate both surrogate chemicals for fitting qNTA models and an evaluation set for verifying qNTA performance. The size of the evaluation set should be decided with consideration of the reported confidence level (e.g., 97.5% confidence can only be achieved with at least 40 chemicals in a test set). Appropriate surrogate and evaluation sets should have a composition resembling that of the chemicals on which qNTA predictions will be made. Given the lower-than-expected reliability of the expert selection approach demonstrated here (A4), an unbiased methodology for determining surrogate appropriateness is still needed.

### Supplementary Information

Below is the link to the electronic supplementary material.

Supplementary file1 (ZIP 7749 KB)
