# Supplementary material for: Establishing performance metrics for quantitative non-targeted analysis: a demonstration using per- and polyfluoroalkyl substances
Source: Anal Bioanal Chem. 2024 Jan 30;416(5):1249–67. doi: 10.1007/s00216-023-05117-4 (PMC10850229; doi:10.1007/s00216-023-05117-4)
Supplement: Supplementary file 1 — Supplementary file1 (ZIP 7749 KB) [file 216_2023_5117_MOESM1_ESM.zip › ABC-01752-2023-ESM_File_3-final.docx]

**Supporting Information**

**Establishing performance metrics for quantitative non-targeted analysis: a demonstration using per- and poly-fluoroalkyl substances**

Shirley Pu^1,2*^, James P. McCord^3*^, Jacqueline Bangma^3^, Jon R. Sobus^1*^

^1^U.S Environmental Protection Agency, Office of Research and Development, Center for Computational Toxicology and Exposure, 109 TW Alexander Dr., Research Triangle Park, NC 27711, USA

^2^Oak Ridge Institute for Science and Education (ORISE) Participant, 109 T.W Alexander Drive, Research Triangle Park, NC 27711, USA

^3^U.S Environmental Protection Agency, Office of Research and Development, Center for Environmental Measurement and Modeling, 109 TW Alexander Dr., Research Triangle Park, NC 27711, USA

*Authors to whom correspondence should be addressed:

Shirley Pu ([pu.shirley@epa.gov](mailto:pu.shirley@epa.gov)); ORCID: 0000-0002-0122-3797

James McCord ([mccord.james@epa.gov](mailto:mccord.james@epa.gov)); ORCID: 0000-0002-1780-4916

Jon Sobus ([sobus.jon@epa.gov](mailto:sobus.jon@epa.gov)); ORCID: 0000-0003-0740-6604

1. **Chromatographic Conditions**

Chromatographic separation was performed using an attached Vanquish UPLC system (Thermo Fisher Scientific, Waltham, MA) and heated Raptor C18 (100 x 3.0mm x 2.7um), at a flow rate of 300 µL/min, column temperature of 50 °C, injection volumes of 25 µL, and a binary mobile phase gradient composed of Solvent A (95:5 H_2_O:ACN 2.5 mM ammonium acetate) and Solvent B (5:95 H_2_O:ACN, 2.5 mM ammonium acetate). The separation gradient was set as: 3 min pre-equilibration at 10% B; 0-15 min linear gradient from 10%-100% B; 15min-20min held at 100% B. BDS-Hypersil isolator column (50mm x 30mm x 5um particles) was installed between the mixing chamber and the analytical column to suppress background PFAS contaminants from the LC system.

1. **MS Instrument Conditions**

PFAS detection was carried out on a Thermo Vanquish UPLC System coupled to an Orbitrap Fusion Mass Spectrometer operating in negative electrospray ionization mode using a H-ESI source and a 20-minute method. The method was based on previously developed quantitative methods [1].

Global Mass Spectrometry settings on the Orbitrap Fusion included negative mode ionization, default charge state -1, and EASY-IC as the lock-mass source. Electrospray ionization source conditions were static -2000V with sheath gas settings 25 Arb (arbitrary units), aux gas settings 8 Arb, and sweep gas settings 2 Arb. Ion transfer tube was held at 325 °C and a heated vaporizer temperature 300 °C.

Scan settings for the data collection were an MS1 Orbitrap only scan with mass range 200-2000 m/z and data collection in profile mode at 120,000 resolving power and quadrupole isolation. AGC was set to 1e6 with Auto as the maximum injection time. RF lens was set to 60%.

1. **Targeted Analysis Workflow- Thermo Xcalibur Quan**

Peak integration was based on extracted ion chromatograms from the major observed ions and in-source fragments for the PFAS. These include [M-H]^-^ and [M-CO2-H]^-^ ions, and, for PFMOAA, the dimer and sodiated dimer ions. Exact masses are reported in the table below. Mass tolerance was set to 6ppm. Computerized integration using Genesis was applied as a first pass, with manual integrations necessary to include isomer peaks (for PFOA, PFOS, NBP2, PFHxS) and to account for some non-ideal chromatographic peak shapes (e.g., PFBA, PFMOAA)

The following masses and retention times were used for compound identification:

| Compound | Mass(es) (m/z) | Retention time (min) |
| --- | --- | --- |
| PFMOAA | 358.9619, 380.9438 | 2.99 |
| MPFBA | 216.9949 | 4.17 |
| PFBA | 212.9793 | 4.17 |
| PFECA-F | 228.9741 | 5.20 |
| M5PFPeA | 267.9922 | 6.35 |
| PFPeA | 218.9862, 262.9757 | 6.35 |
| PFECA-A | 278.9709 | 6.78 |
| 4:2 FTS | 326.9740 | 7.16 |
| M2-4:2_FTS | 328.9807 | 7.16 |
| M3PFBS | 301.9527 | 7.50 |
| PFBS | 298.9427 | 7.50 |
| M5PFHxA | 317.9893 | 7.54 |
| PFHxA | 268.9830, 312.9725 | 7.54 |
| HFPODA | 284.9778, 328.9677 | 7.90 |
| M3HFPO-DA | 286.9841, 331.9772 | 7.90 |
| M4PFHpA | 366.9827 | 8.49 |
| PFHpA | 318.9798, 362.9693 | 8.49 |
| PFPeS | 348.9395 | 8.58 |
| 6:2 FTS | 426.9676 | 8.99 |
| M2-6:2_FTS | 428.9743 | 8.99 |
| M8PFOA | 420.9929 | 9.34 |
| PFOA | 368.9766, 412.9661 | 9.34 |
| M3PFHxS | 401.9463 | 9.49 |
| PFHxS | 398.9363 | 9.49 |
| NBP2 | 462.9326 | 10.00 |
| M3PFNA | 471.9931 | 10.15 |
| PFNA | 418.9734, 462.9629 | 10.15 |
| PFHpS | 448.9331 | 10.34 |
| 8:2_FTS | 526.9622 | 10.55 |
| M2-8:2_FTS | 528.9679 | 10.55 |
| M6PFDA | 518.9798 | 10.91 |
| PFDA | 468.9702, 512.9597 | 10.91 |
| d3-N-MeFOSAA | 572.9800 | 11.11 |
| M8PFOS | 506.9567 | 11.11 |
| N-MeFOSAA | 569.9670 | 11.11 |
| PFOS | 498.9299 | 11.11 |
| d5-N-EtFOSAA | 589.0140 | 11.49 |
| N-EtFOSAA | 583.9827 | 11.49 |
| M7PFUnDA | 569.9800 | 11.65 |
| PFUnDA | 562.9565 | 11.65 |
| PFNS | 548.9267 | 11.86 |
| M2PFDoDA | 614.9600 | 12.37 |
| PFDoDA | 612.9533 | 12.37 |
| PFDS | 598.9235 | 12.58 |
| PFTrDA | 662.9501 | 13.06 |
| M8PFOSA | 505.9727 | 13.66 |
| PFOSA | 497.9459 | 13.66 |
| M2PFTeDA | 669.9604 | 13.73 |
| PFTeDA | 668.9576, 712.9482 | 13.73 |

1. **Non-targeted Analysis Workflow - Compound Discoverer**

Compound Discoverer settings were based on the default template “Environmental Unknown ID w Local Database Searchers” with specific modifications noted below. The complete workflow, including default settings, is included in Supplementary Information File 4.

*Align Retention Times (ChromAlign)*: The Cal125-1 file was used as the reference file for aligning retention times using ChromAlign.

*Detect Compounds*: RT tolerance was set to 1.0 minute. Mass tolerance was set to 10ppm. The minimum peak intensity was 1000000. The minimum # of scans per peak was set to 5. Only the area for the most intense isotope peak in an isotope pattern was reported.

*Merge features*: Mass tolerance for peak consolidation was set to 5ppm. The RT tolerance for peak merging was set to 0.25 minutes.

*Formula Generation*: Maximum allowable atoms for formula generation were C90 H190 Br3 Cl8 F30 N10 O18 P3 S5 and intensity pattern matching tolerance was 30% with a minimum spectral fit of 30%.

1. **Non-targeted Analysis Workflow - Manual Peak Integration**

Integration errors for certain compounds were observed in Compound Discoverer. M2-6:2_FTS experiences interference from the isotopic contribution of sulfur at high native concentrations (<50ng/mL), leading to poor peak picking and merging with the native 6:2 FTS peak. PFDoDA was manually integrated at low concentrations (<2 ng/mL) due to poor peak shape. To correct these areas, the peaks for the primary ions were located in the chromatogram using Xcalibur Qual Browser and the areas were manually integrated as described in the Xcalibur processing method.

**6.0 Excluded Data Points**

The following data points were excluded from further analysis:

| Compound | Excluded Data | Reason |
| --- | --- | --- |
| PFBA | 0.98 ng/mL (replicates 1,2,3); 1.95 ng/mL (replicates 1,2,3); 3.90 ng/mL (replicates 1,2,3) | Low concentration amounts are outside the linear range of the calibration curve |
| PFMOAA | 0.98 ng/mL (replicates 1,2,3); 1.95 ng/mL (replicates 1,2,3); 3.90 ng/mL (replicates 1,2,3) | Low concentration amounts are outside the linear range of the calibration curve |
| PFOSA | 0.98 ng/mL (replicates 1,2,3) | Outliers (potential carryover) |
| M8PFOSA | 0.98 ng/mL (replicates 1,2,3) | Outliers (potential carryover) |
| N-EtFOSAA | 0.98 ng/mL (replicates 1,2,3); 1.95 ng/mL (replicates 1,2,3); 3.90 ng/mL (replicates 1,2,3) | Low concentration amounts are outside the linear range of the calibration curve |
| N-MeFOSAA | 0.98 ng/mL (replicates 1,2,3); 1.95 ng/mL (replicates 1,2,3); 3.90 ng/mL (replicates 1,2,3) | Low concentration amounts are outside the linear range of the calibration curve |
| PFECA-F | 0.98 ng/mL (replicates 1,2,3); 1.95 ng/mL (replicates 1,2,3) | Low concentration amounts are outside the linear range of the calibration curve |
| PFHxA | 0.96 ng/mL (replicates 1,2,3); 1.95 ng/mL (replicates 1,2,3);  3.90 ng/mL (replicates 1,2,3) | Low concentration amounts are outside the linear range of the calibration curve |
| PFPeS | 0.96 ng/mL (replicates 1,2,3); 1.95 ng/mL (replicates 1,2,3);  3.90 ng/mL (replicates 1,2,3) | Low concentration amounts are outside the linear range of the calibration curve |

**7.0 Limitations of Absolute Relative Error (*ARE*) as qNTA Accuracy Metric**

When $\hat{Conc}\ll{Conc}_{True}$, the magnitude of $\left| \hat{Conc}-{Conc}_{True} \right|$ approaches the magnitude of ${Conc}_{True}$, such that ($\left| \hat{Conc}-{Conc}_{True} \right|/{Conc}_{True})$ approaches unity. As predicted chemical concentrations are never lower than zero (i.e., they cannot be negative), the *ARE* is eventually bounded at a value of ~100% for predicted concentrations near zero. Targeted methods are unlikely to exhibit such high differences between $\hat{Conc}$ and ${Conc}_{True}$ due to their use of matched chemical standards and calibration curve quality control protocols. However, qNTA approaches, which use surrogate calibration data, may have point concentration estimates that are extremely small (but still greater than zero) and differ by orders-of-magnitude from the true concentrations. Given the potential for a large disparity between $\hat{Conc}$ and ${Conc}_{True}$, it is necessary to evaluate the fold-difference (i.e., $\hat{Conc}/{Conc}_{True}$) rather than the absolute difference to communicate qNTA method accuracy. For the current work, the fold-differences between $\hat{Conc}$ and ${Conc}_{True}$ for all five approaches are given as *Accuracy Quotient* (*AQ*) and *Absolute Accuracy Quotient* (*AAQ*) values.

**8.0 Secondary Use Case: Focusing on Lower Confidence Limit Uncertainty (*LCQ*) and Reliability (*LRP*)**

For quantitation tasks which focus on the consequences of overprediction, identifying whether or not the lower confidence limit lies below the true concentration is of prime importance. We refer to tasks prioritizing the lower confidence limit over the upper confidence limit as the “secondary use case”.

We evaluated uncertainty and reliability for the secondary use case by calculating the Lower Confidence Quotient (*LCQ*) and Lower Reliability Percentage (*LRP*) for all quantitative approaches. The *LCQ* values for A1-A5 were compared in a pairwise manner using one-way random effects models and Wilcoxon signed-rank tests. The majority of approaches had statistically different *LCQ* values (Table 1). Yet, *LCQ* values across A2 and A3 were found to be statistically indistinguishable based on results of both the random-effects model and the Wilcoxon signed-rank test.

The calculated *LRP* is expected to be ~97.5% for each inverse prediction approach. The *LRP* values were 95.1%, 99.7%, 98.2%, 88.1%, and 95.9% for A1, A2, A3, A4, and A5, respectively. *LRP*s differed more from the expected 97.5% than did *URP*s, with four approaches (A1, A2, A4, A5) having *LRP*s that differed by more than 1% from the theoretical value.

Chemical-specific *LRP* values were 100% for many compounds in A2-A5; more variable *LRP* values were observed for A1. Chemical-specific *LRP* values were as low as 88.9% (HFPODA) for A1, 96.2% (PFPeS) for A2, 88.9% (PFDoDA) for A3, 0% (NBP2) for A4, and 0% (NBP2) for A5. Notably, while A4 produced several chemicals with lower *LRP* values (n=9 chemicals with *LRP* < 90%), A5 produced *LRP* values of 100% for all chemicals except NBP2.

**9.0 Results for Approach Comparisons (A1-A5) Using the Wilcoxon Signed-Rank Test**

|  | **Absolute Accuracy Quotient (*AAQ*)** | | | **Confidence Limit Fold Range (*CLFR*)** | | | **Upper Confidence Quotient (*UCQ*)** | | | **Lower Confidence Quotient (*LCQ*)** | | |
| --- | --- | --- | --- | --- | --- | --- | --- | --- | --- | --- | --- | --- |
| **Approach Comparison** | $\boldsymbol{10}^{\hat{\boldsymbol{\mu}}}$  (~fold-difference^1^) | **p-value** | **n** | $\boldsymbol{10}^{\hat{\boldsymbol{\mu}}}$  (~fold-difference) | **p-value** | **n** | $\boldsymbol{10}^{\hat{\boldsymbol{\mu}}}$  (~fold-difference) | **p-value** | **n** | $\boldsymbol{10}^{\hat{\boldsymbol{\mu}}}$  (~fold-difference) | **p-value** | **n** |
| **A2 vs. A1** | 1.13 | **p < 0.001** | 20 | 1.92 | **p < 0.001** | 20 | 1.41 | **p < 0.001** | 20 | 1.39 | **p < 0.001** | 20 |
| **A3 vs. A1** | 1.14 | **p < 0.001** | 19 | 2.07 | **p < 0.001** | 19 | 1.46 | **p < 0.001** | 19 | 1.46 | **p < 0.001** | 19 |
| **A4 vs. A1** | 1.99 | **p < 0.001** | 19 | 17.7 | **p < 0.001** | 19 | 3.67 | **p < 0.001** | 19 | 2.49 | **p = 0.001** | 19 |
| **A5 vs. A1** | 2.50 | **p < 0.001** | 19 | 1030 | **p < 0.001** | 19 | 71.1 | **p < 0.001** | 18 | 16.6 | **p < 0.001** | 19 |
| **A3 vs. A2** | 1.01 | p = 0.468 | 26 | 1.04 | p = 0.208 | 26 | 1.04 | p = 0.084 | 26 | 1.00 | p = 0.861 | 26 |
| **A4 vs. A2** | 2.17 | **p < 0.001** | 26 | 7.74 | **p < 0.001** | 26 | 2.56 | **p < 0.001** | 26 | 1.56 | **p = 0.037** | 25 |
| **A5 vs. A2** | 2.74 | **p < 0.001** | 26 | 526 | **p < 0.001** | 26 | 72.0 | **p < 0.001** | 24 | 10.2 | **p < 0.001** | 25 |
| **A4 vs. A3** | 2.16 | **p < 0.001** | 26 | 7.57 | **p < 0.001** | 26 | 2.41 | **p = 0.001** | 26 | 1.46 | p = 0.101 | 25 |
| **A5 vs. A3** | 2.62 | **p < 0.001** | 26 | 476 | **p < 0.001** | 26 | 64.8 | **p < 0.001** | 24 | 9.87 | **p < 0.001** | 25 |
| **A5 vs. A4** | 1.46 | **p = 0.038** | 26 | 69.3 | **p < 0.001** | 26 | 12.1 | **p < 0.001** | 24 | 5.67 | **p < 0.001** | 25 |

^1^The Wilcoxon test estimates the pseudo-median of the paired differences, $\hat{\mu}$. The value ${10}^{\hat{\mu}}$ provides an approximate estimate of the median pairwise fold-difference.

**
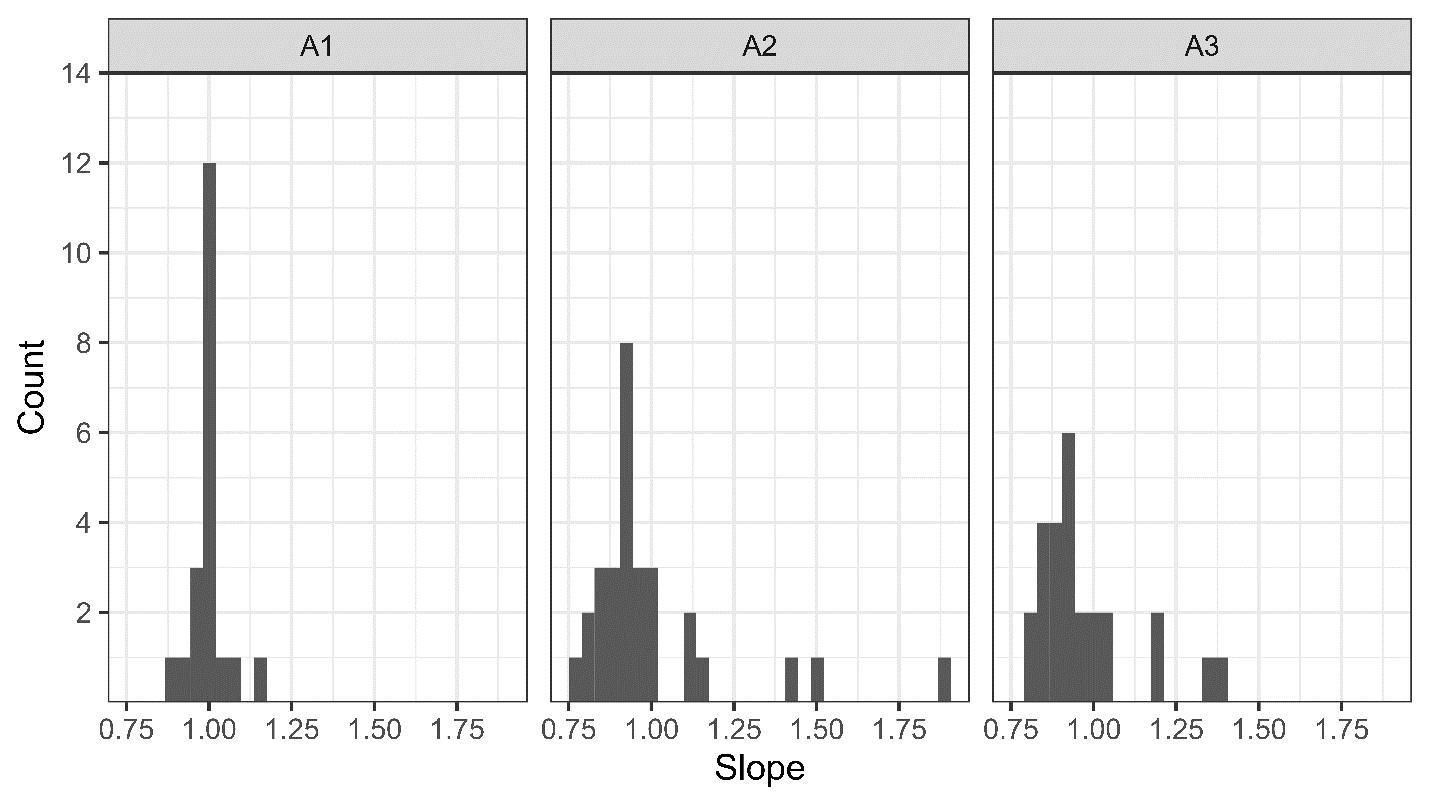
**

**
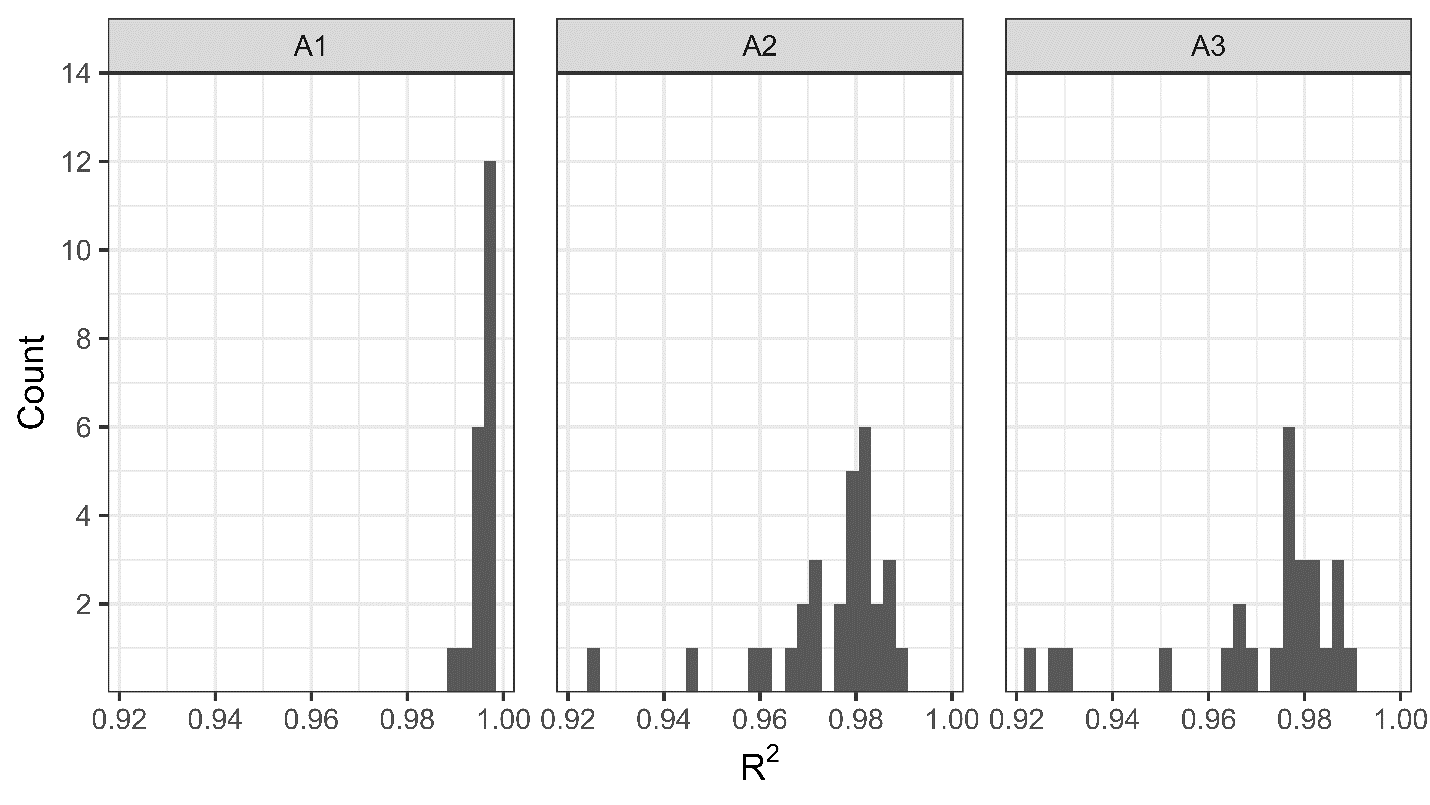
**

**Figure S1.** Distribution of slopes (top) and R^2^ (bottom) for A1-A3 calibration curves. The spread of calibration curve parameters was smallest for A1, which used internal standard normalization, and similar for A2 and A3, which only differed in choice of data processing method (targeted vs. non-targeted).

**
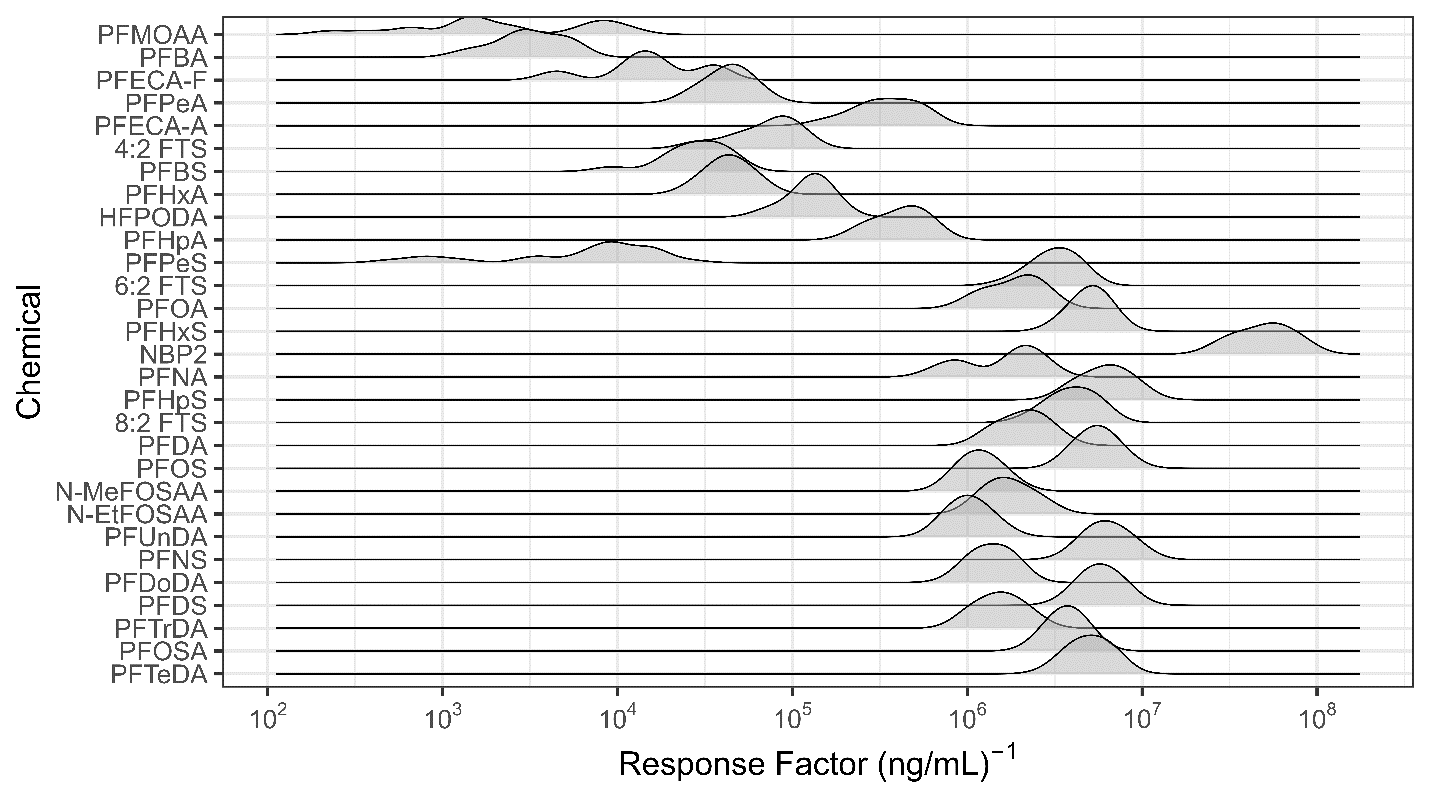
**

**Figure S2.** Response factor (RF) distributions (n=29) of chemical data processed using targeted analysis software (Xcalibur Quan). Chemicals are ordered, from top to bottom, based on increasing retention time. Per-chemical RF distributions are largely log-normal, but select chemicals (PFPeS, PFECA-F, PFMOAA) exhibit wide flat distributions. Distribution of overall chemical RFs is apparently multimodal and spans six orders of magnitude (10^2^ to 10^8^).


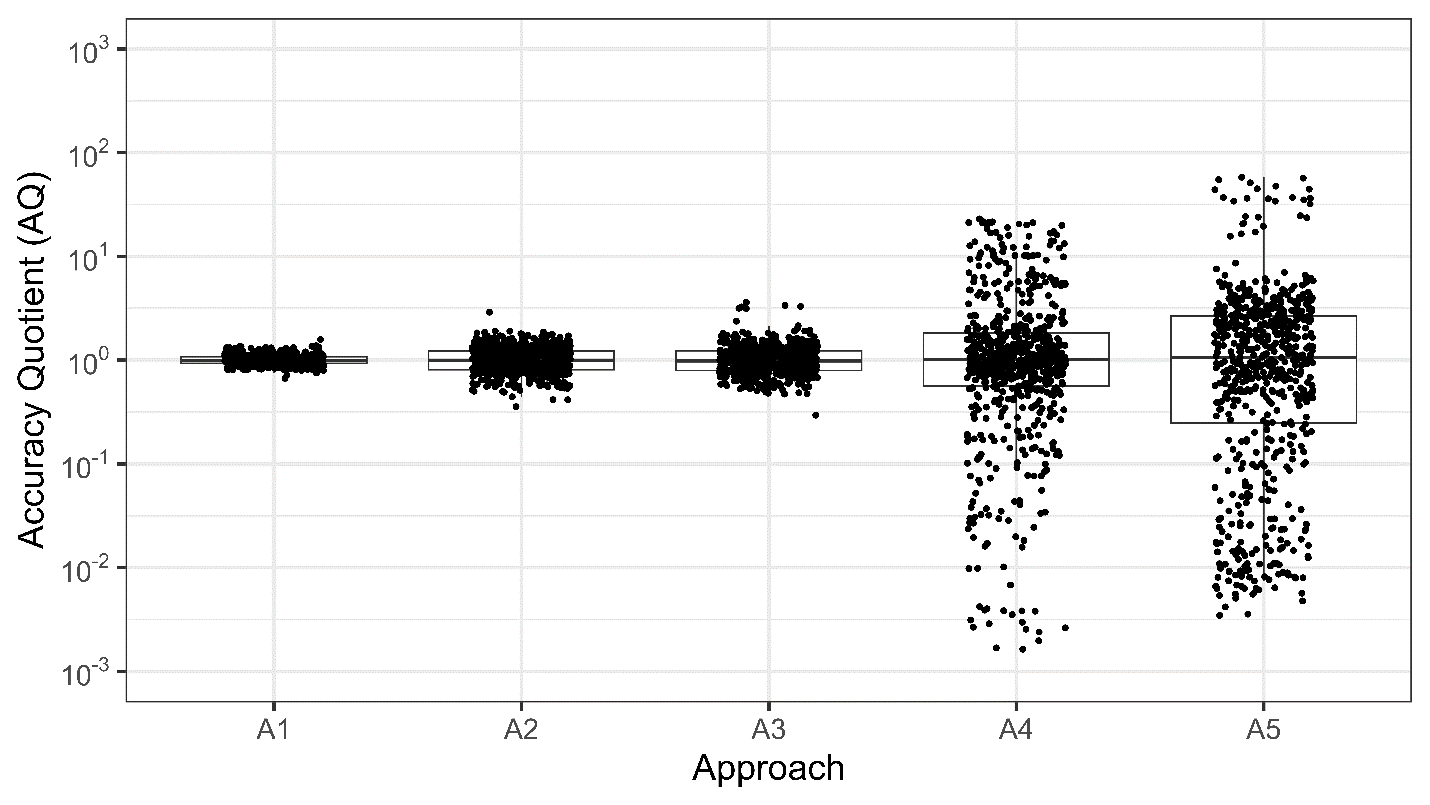


**Figure S3.** Boxplot with individual point scatter of *Accuracy Quotient* (*AQ*) values for A1-A5.


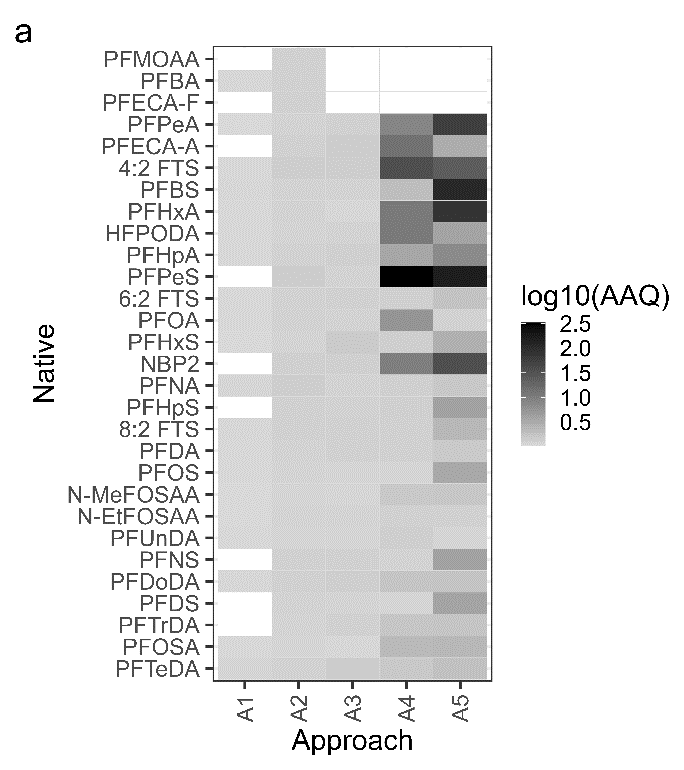

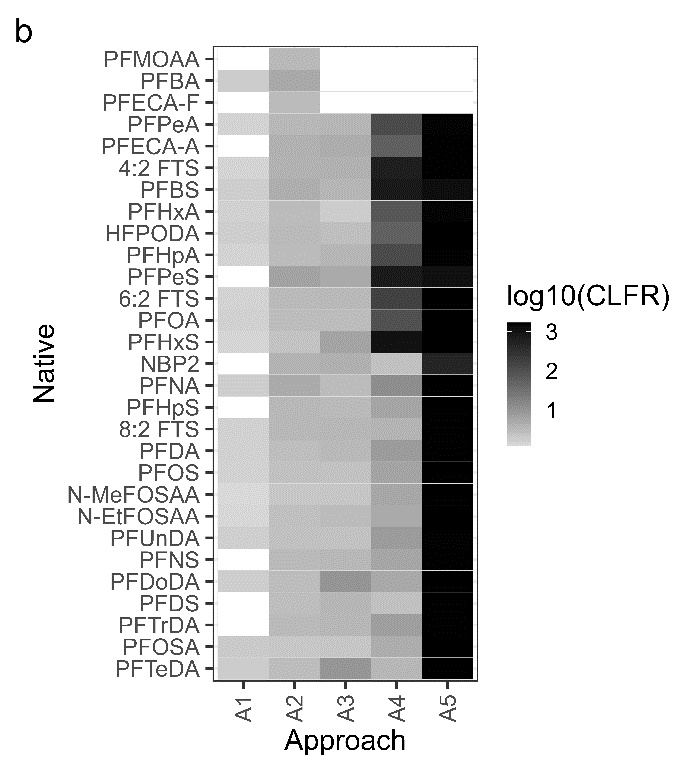


**
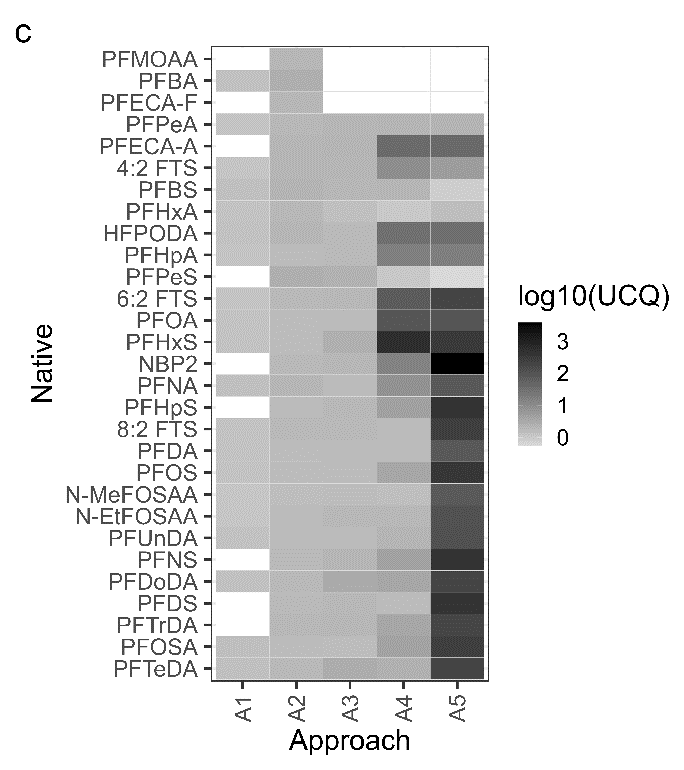

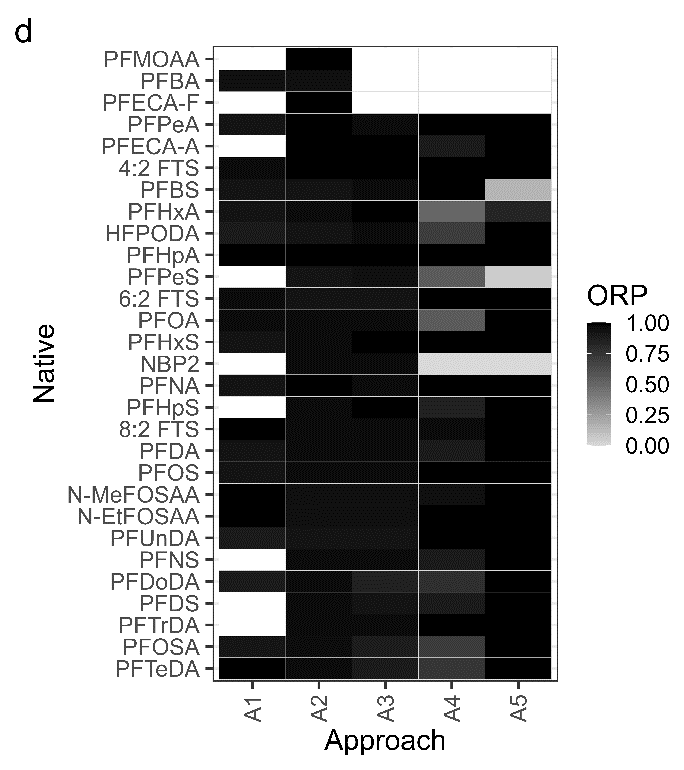
**

**Figure S4.** Heatmaps of log-transformed *Absolute Accuracy Quotient* (log_10_[*AAQ*]; panel a), log-transformed *Confidence Limit Fold Range* (log_10_[*CLFR*]; panel b), log-transformed *Upper Confidence Quotient* (log_10_[*UCQ*]; panel c), and *Overall Reliability Percentage* (*ORP*; panel d). Heatmaps were created in R using the *geom_tile* function from the package *ggplot2*.

**References**

1. McCord JP, Strynar MJ, Washington JW, Bergman EL, Goodrow SM. Emerging Chlorinated Polyfluorinated Polyether Compounds Impacting the Waters of Southwestern New Jersey Identified by Use of Nontargeted Analysis. Environ Sci Technol Lett. 2020;7(12):903-8.
