# Supplementary material for: Establishing performance metrics for quantitative non-targeted analysis: a demonstration using per- and polyfluoroalkyl substances
Source: Anal Bioanal Chem. 2024 Jan 30;416(5):1249–67. doi: 10.1007/s00216-023-05117-4 (PMC10850229; doi:10.1007/s00216-023-05117-4)
Supplement: Supplementary file 1 — Supplementary file1 (ZIP 7749 KB) [file 216_2023_5117_MOESM1_ESM.zip › Pu_et_al_2023_qNTA_metrics_Supplementary_File_1.pdf]

Supplementary File 1

Article: "Establishing performance metrics for quantitative non-targeted analysis: a demonstration using per- and polyfluoroalkyl substances"

Authors: Shirley Pu[1,2*], Jacqueline Bangma[3], James P. McCord[3*], Jon R. Sobus[1*]

Affiliations:

[1]U.S Environmental Protection Agency, Office of Research and Development, Center for Computational Toxicology and Exposure, 109 TW Alexander Dr., Research Triangle Park, NC 27711, USA

[2]Oak Ridge Institute for Science and Education (ORISE) Participant, 109 T.W Alexander Drive, Research Triangle Park, NC 27711, USA

[3]U.S Environmental Protection Agency, Office of Research and Development, Center for Environmental Measurement and Modeling, 109 TW Alexander Dr., Research Triangle Park, NC 27711, USA

*Authors to whom correspondence should be addressed:

Shirley Pu (pu.shirley@epa.gov); ORCID: 0000-0002-0122-3797

James McCord (mccord.james@epa.gov); ORCID: 0000-0002-1780-4916

Jon Sobus (sobus.jon@epa.gov); ORCID: 0000-0003-0740-6604

Approach 1 Calibration Curves
(n=20)

4:2 FTS M2-4:2 FTS Calibration Curve

Log₂(Area Ratio) = -5.4+ 1Log₂(Experimental Amount)
R^2: 0.99782

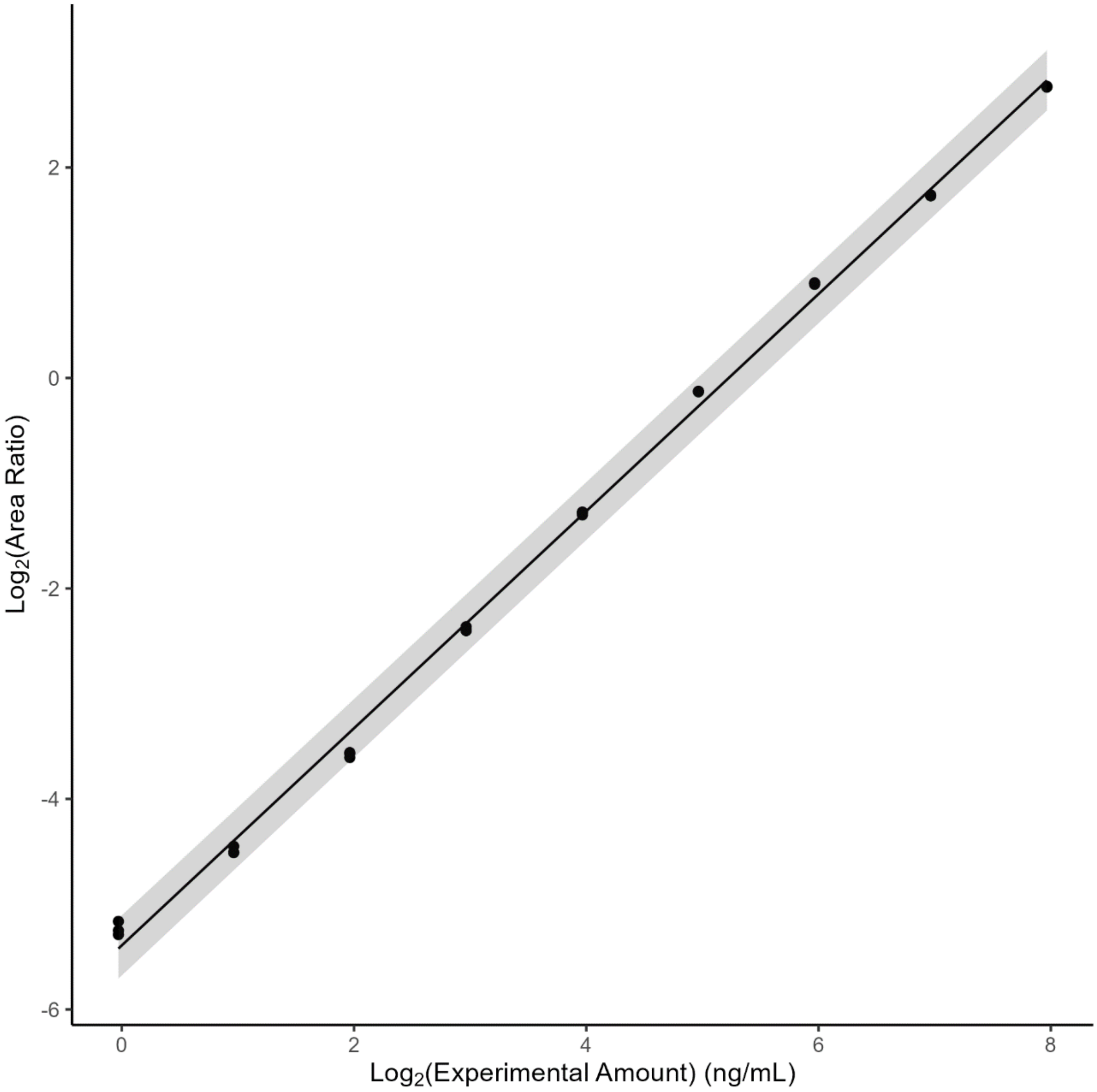

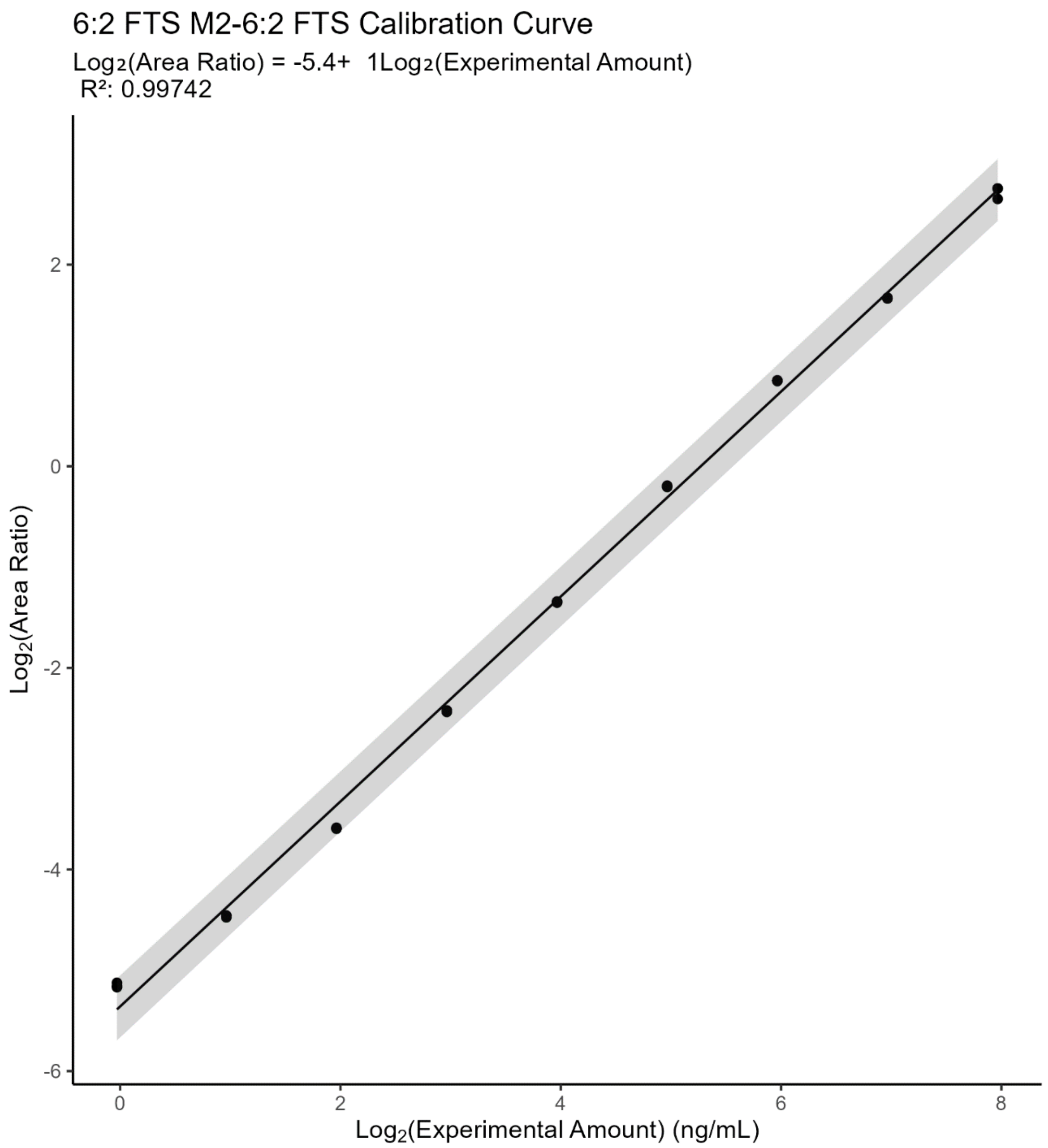

6:2 FTS M2-6:2 FTS Calibration Curve
Log₂(Area Ratio) = -5.4+ 1Log₂(Experimental Amount)
R²: 0.99742
Log2(Area Ratio)
2
0
-2
-4
-6
0
2
4
6
8
Log2(Experimental Amount) (ng/mL)

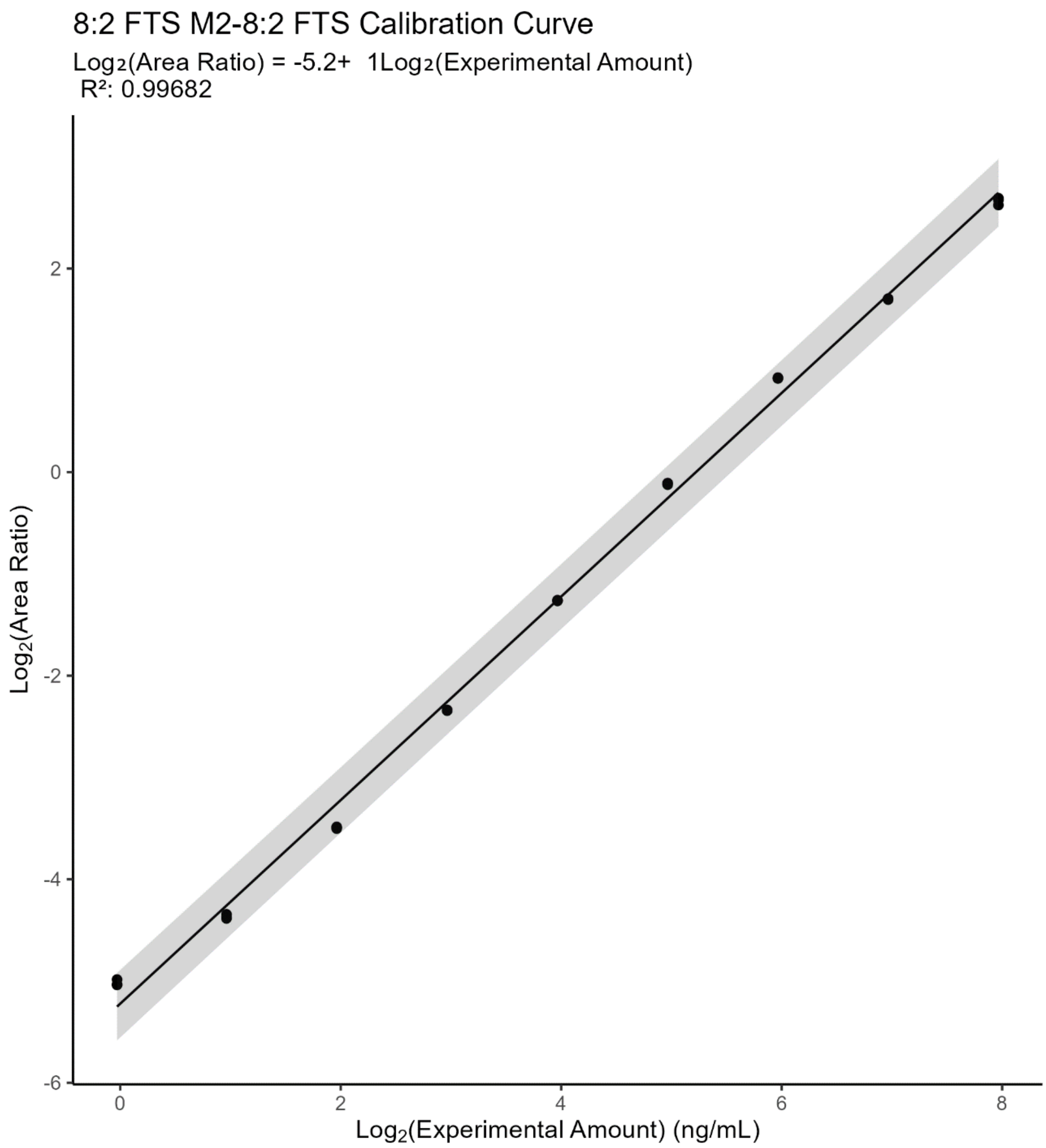

8:2 FTS M2-8:2 FTS Calibration Curve
Log₂(Area Ratio) = -5.2+ 1Log₂(Experimental Amount)
R²: 0.99682
Log2(Area Ratio)
2
0
-2
-4
-6
0
2
4
6
8
Log2(Experimental Amount) (ng/mL)

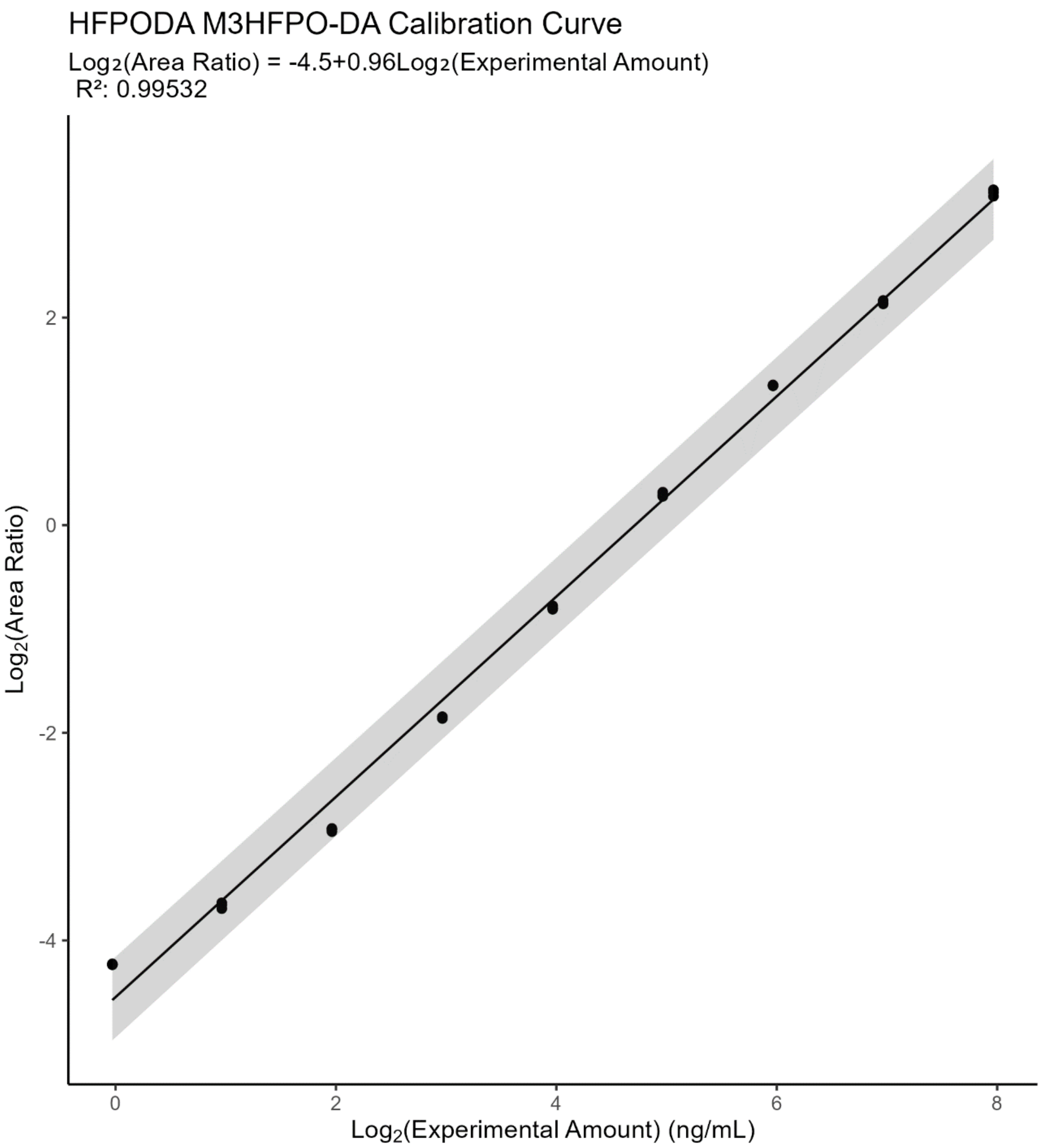
HFPODA M3HFPO-DA Calibration Curve
Log₂(Area Ratio) = -4.5+0.96Log₂(Experimental Amount)
R²: 0.99532
Log2(Area Ratio)
2
0
-2
-4
0
2
4
6
8
Log2(Experimental Amount) (ng/mL)

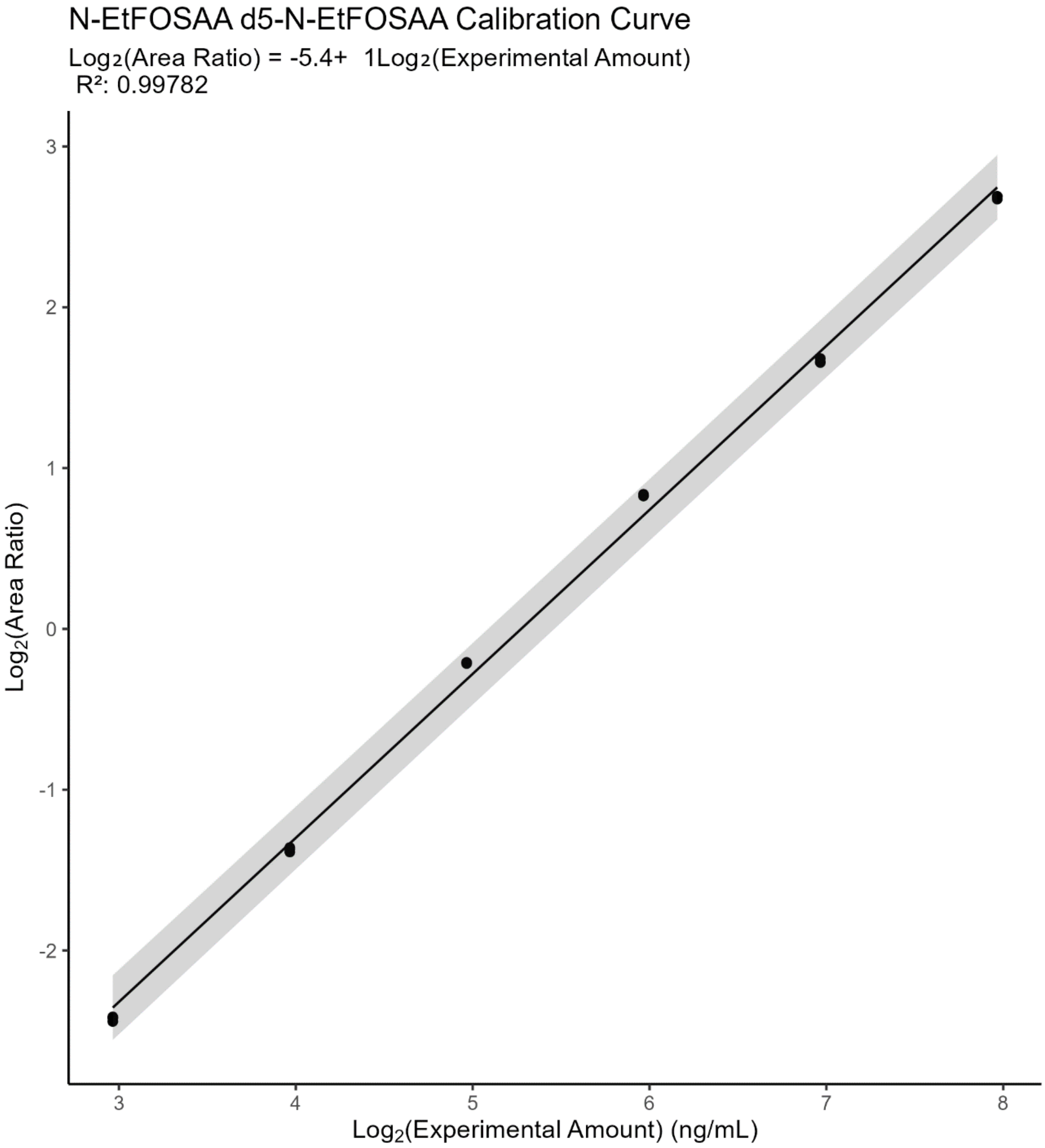

N-EtFOSAA d5-N-EtFOSAA Calibration Curve
Log₂(Area Ratio) = -5.4+ 1Log₂(Experimental Amount)
R²: 0.99782
Log2(Area Ratio)
3
2
1
0
-1
-2
3
4
5
6
7
8
Log2(Experimental Amount) (ng/mL)

N-MeFOSAA d3-N-MeFOSAA Calibration Curve

$Log_2(\text{Area Ratio}) = -5.2 + 1 Log_2(\text{Experimental Amount})$

R^2: 0.99852

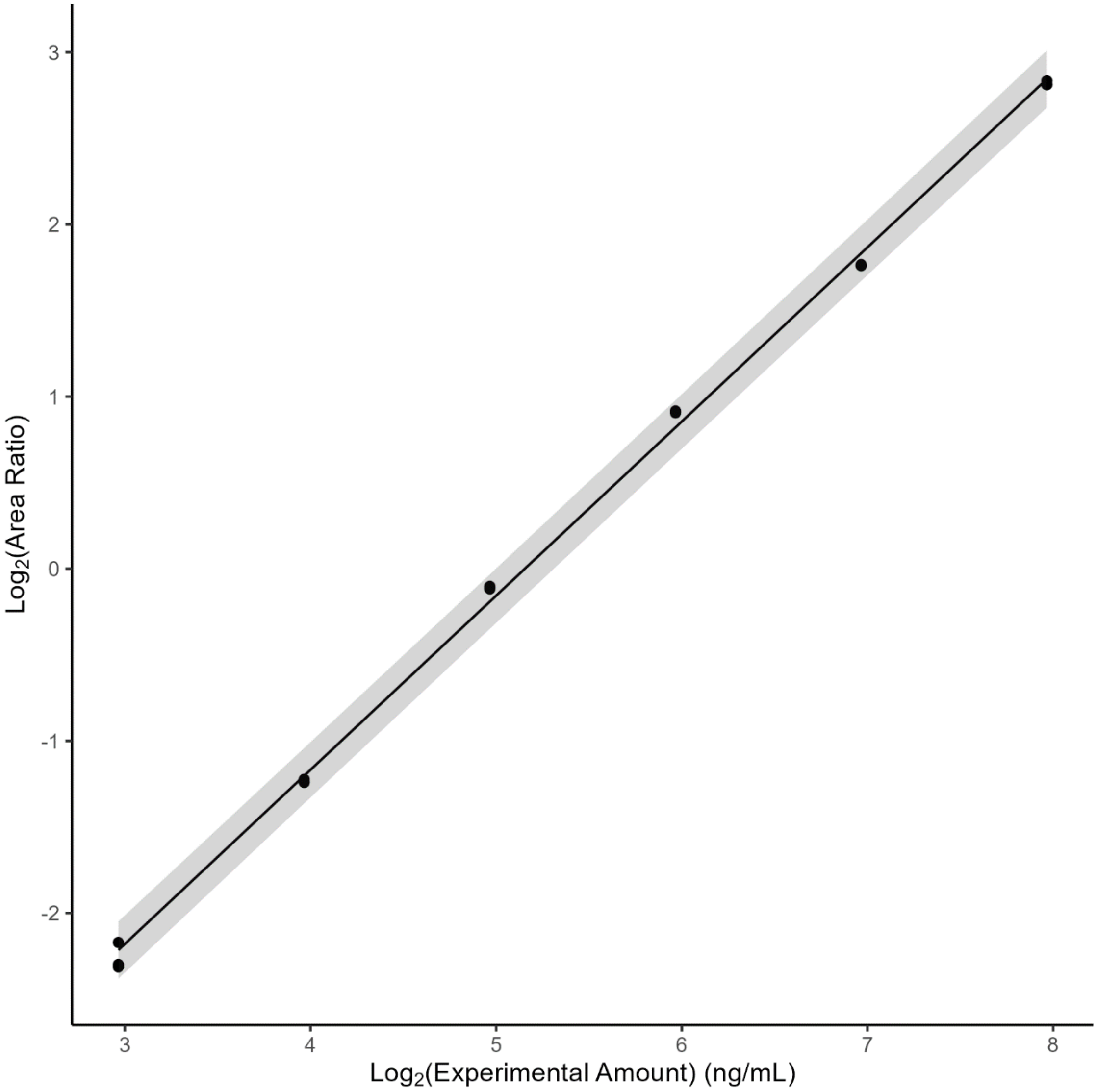

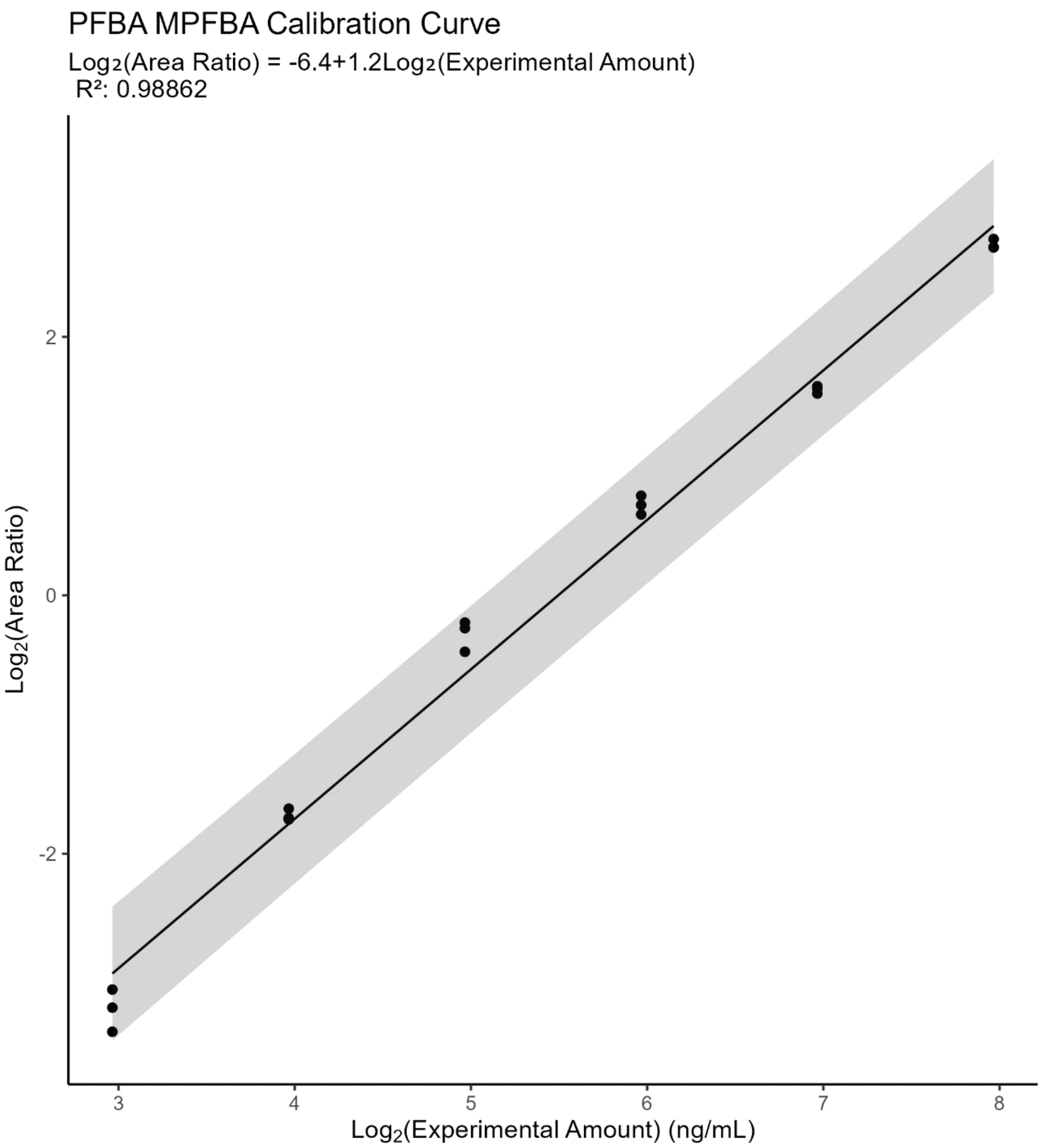

PFBA MPFBA Calibration Curve
Log₂(Area Ratio) = -6.4+1.2Log₂(Experimental Amount)
R²: 0.98862
Log2(Area Ratio)
2
0
-2
3
4
5
6
7
8
Log2(Experimental Amount) (ng/mL)

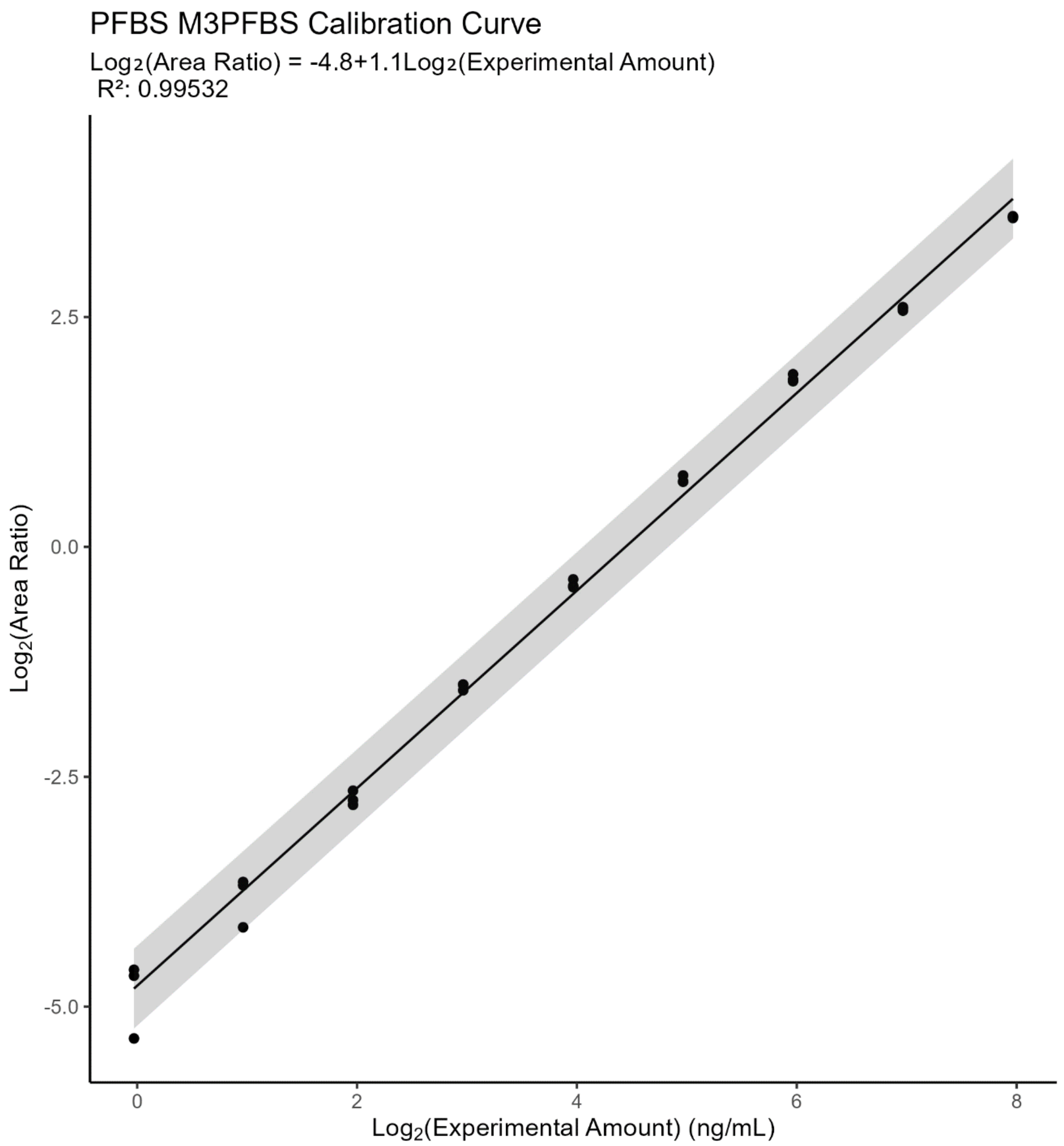
PFBS M3PFBS Calibration Curve
Log₂(Area Ratio) = -4.8+1.1Log₂(Experimental Amount)
R²: 0.99532
Log2(Area Ratio)
2.5
0.0
-2.5
-5.0
0
2
4
6
8
Log2(Experimental Amount) (ng/mL)

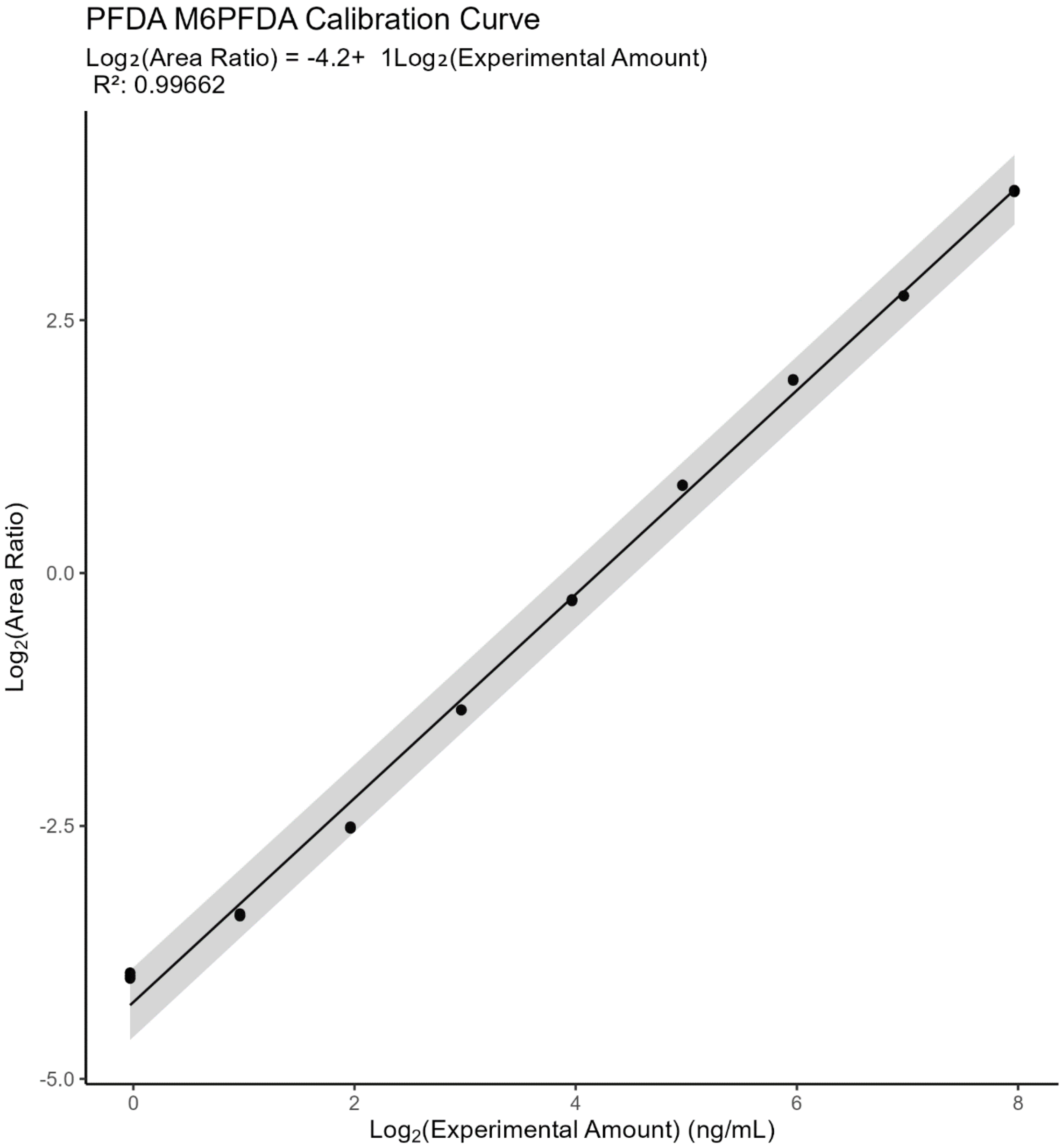
PFDA M6PFDA Calibration Curve
Log₂(Area Ratio) = -4.2+ 1Log₂(Experimental Amount)
R²: 0.99662
Log2(Area Ratio)
2.5
0.0
-2.5
-5.0
0
2
4
6
8
Log2(Experimental Amount) (ng/mL)

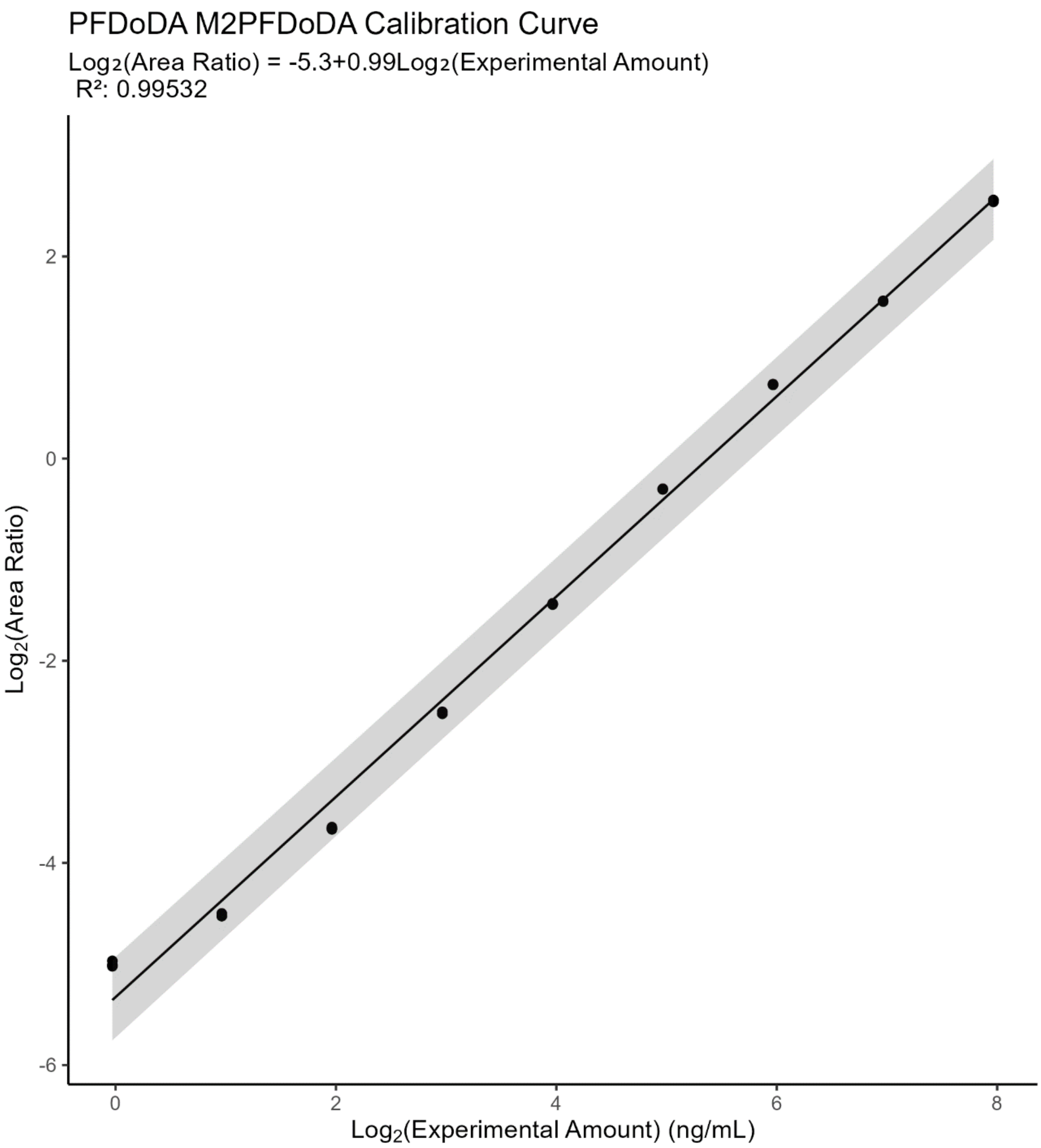
PFDoDA M2PFDoDA Calibration Curve
Log₂(Area Ratio) = -5.3+0.99Log₂(Experimental Amount)
R²: 0.99532
Log2(Area Ratio)
Log2(Experimental Amount) (ng/mL)
2
0
-2
-4
-6
0
2
4
6
8

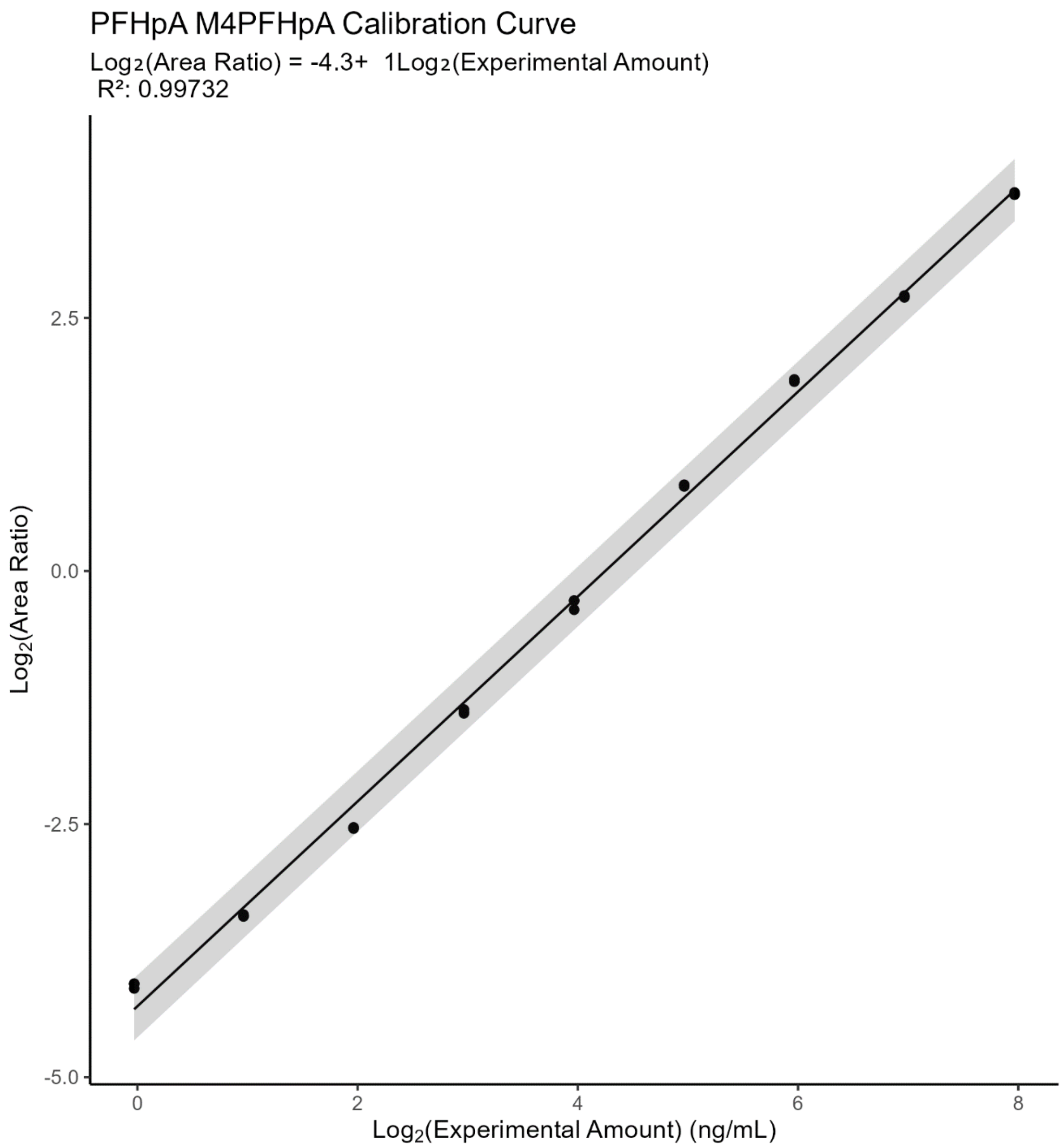

PFHpA M4PFHpA Calibration Curve
Log₂(Area Ratio) = -4.3+ 1Log₂(Experimental Amount)
R²: 0.99732
Log2(Area Ratio)
2.5
0.0
-2.5
-5.0
0
2
4
6
8
Log2(Experimental Amount) (ng/mL)

PFHxA M5PFHxA Calibration Curve

Log_2(Area Ratio) = -5+ 1Log_2(Experimental Amount)
R^2: 0.99682

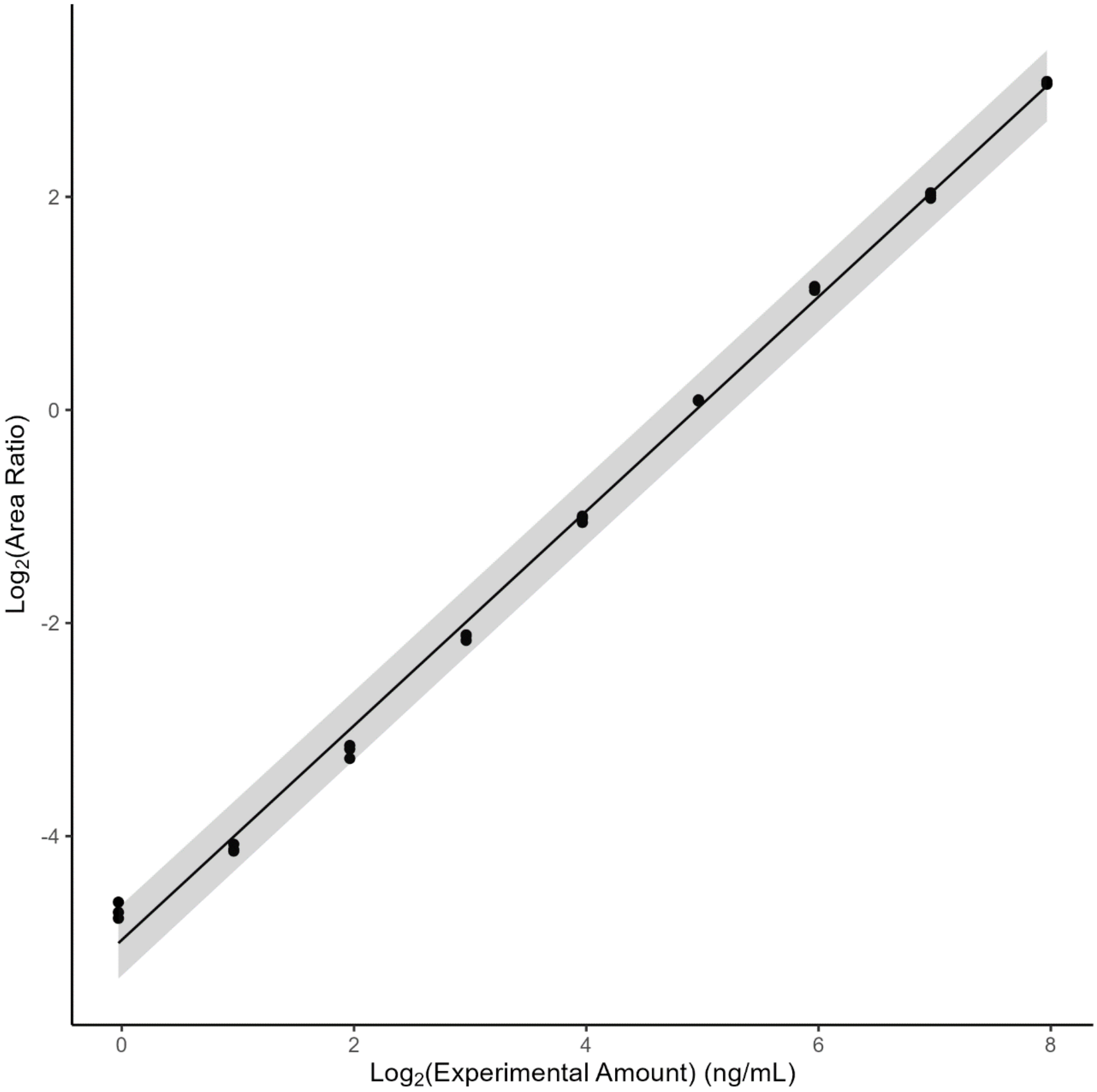

PFHxS M3PFHxS Calibration Curve

Log₂(Area Ratio) = -5.3+ 1Log₂(Experimental Amount)
R^2: 0.9982

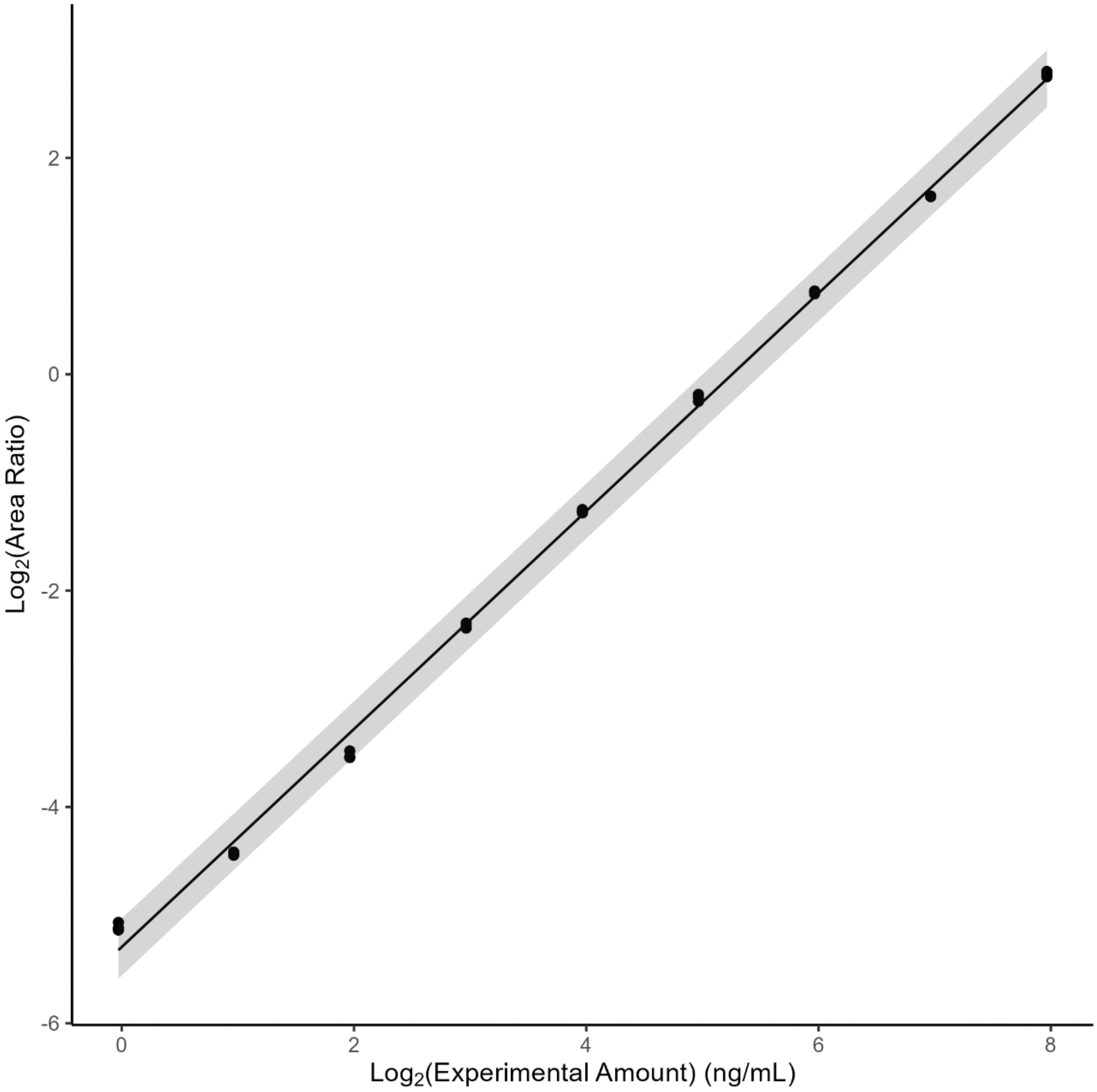

PFNA M9PFNA Calibration Curve

$Log_2(\text{Area Ratio}) = -4+0.92Log_2(\text{Experimental Amount})$

R^2: 0.99522

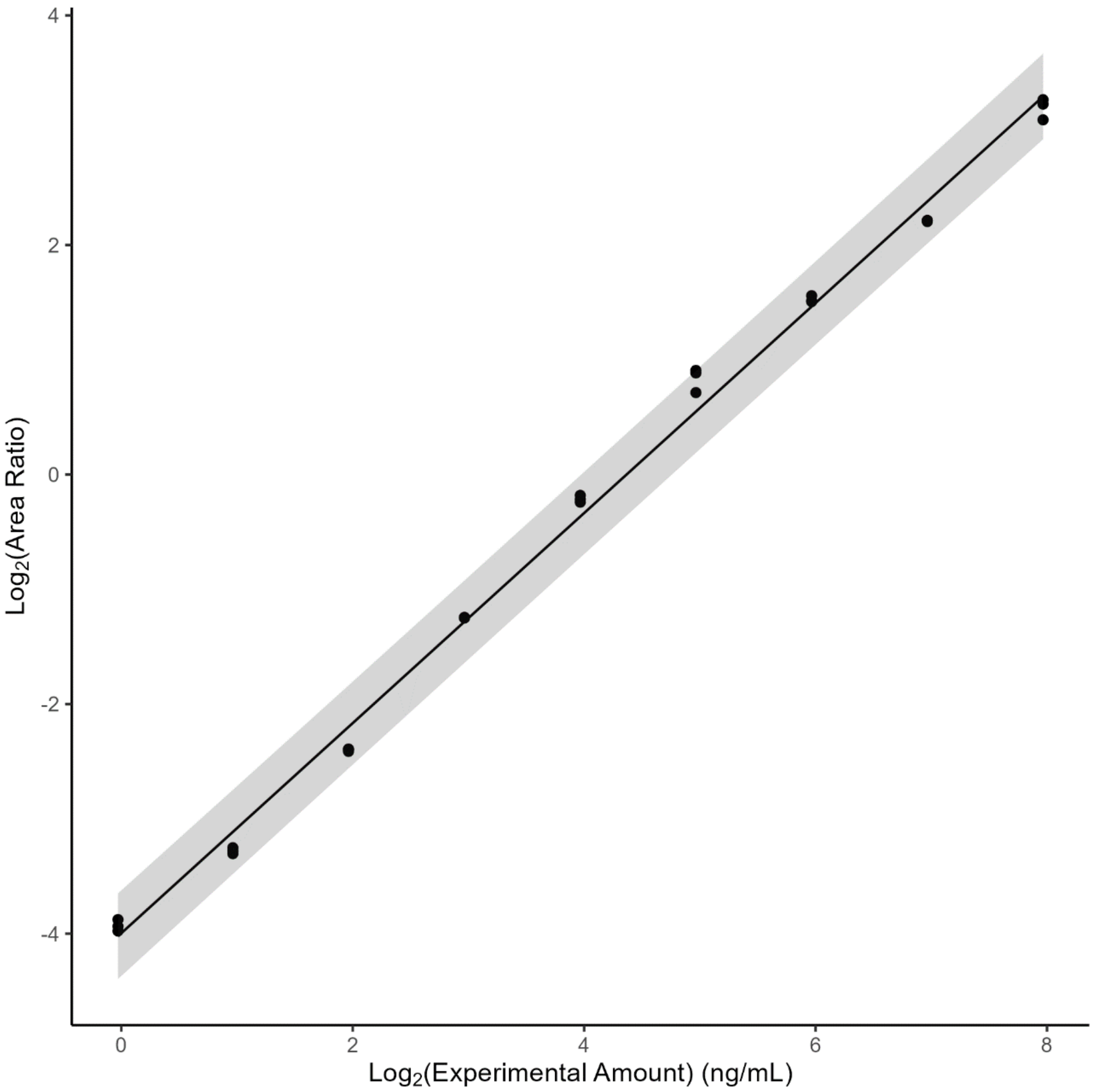

PFOA M8PFOA Calibration Curve

Log_2(Area Ratio) = -3.4+ 1Log_2(Experimental Amount)

R^2: 0.99682

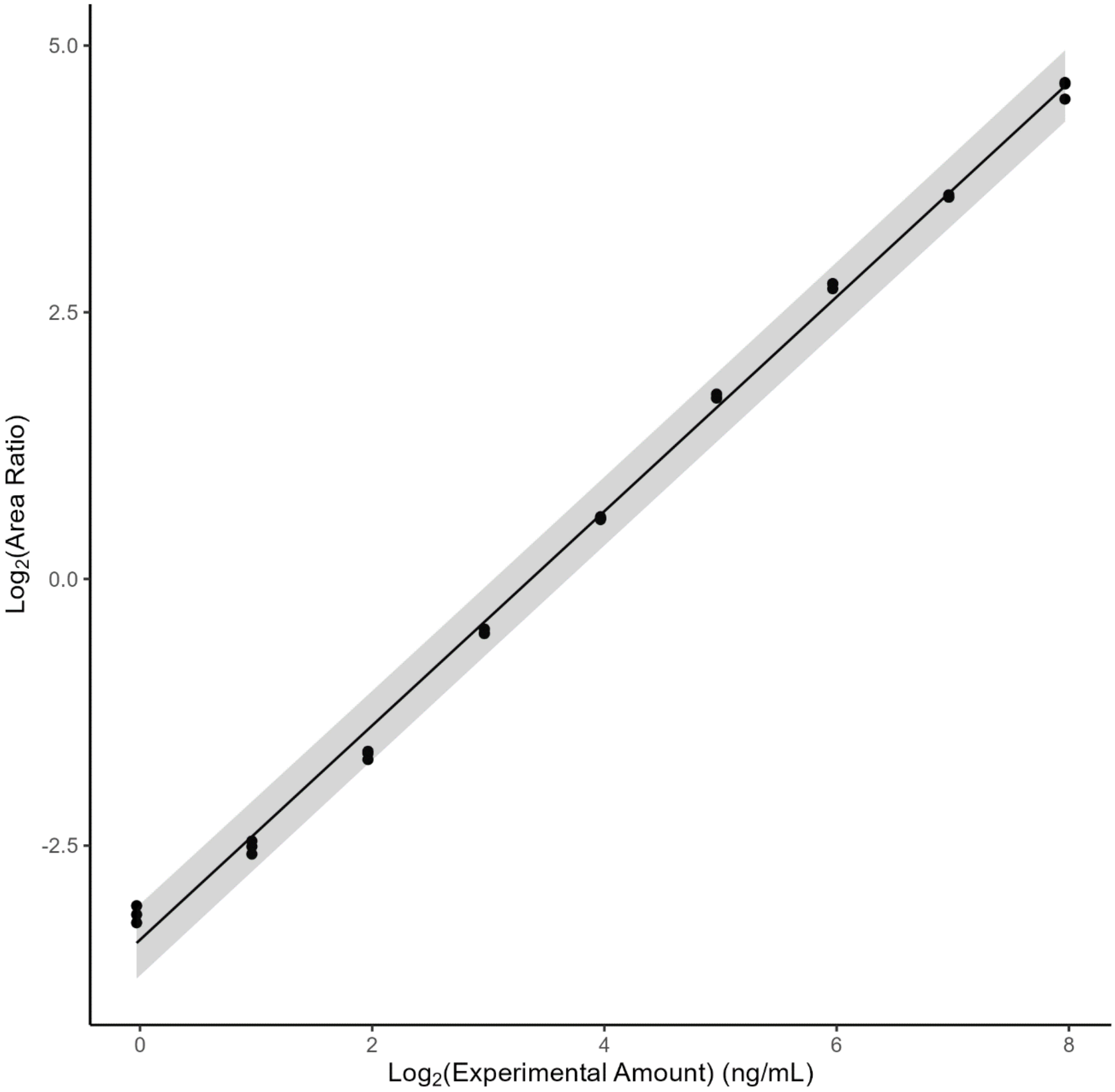

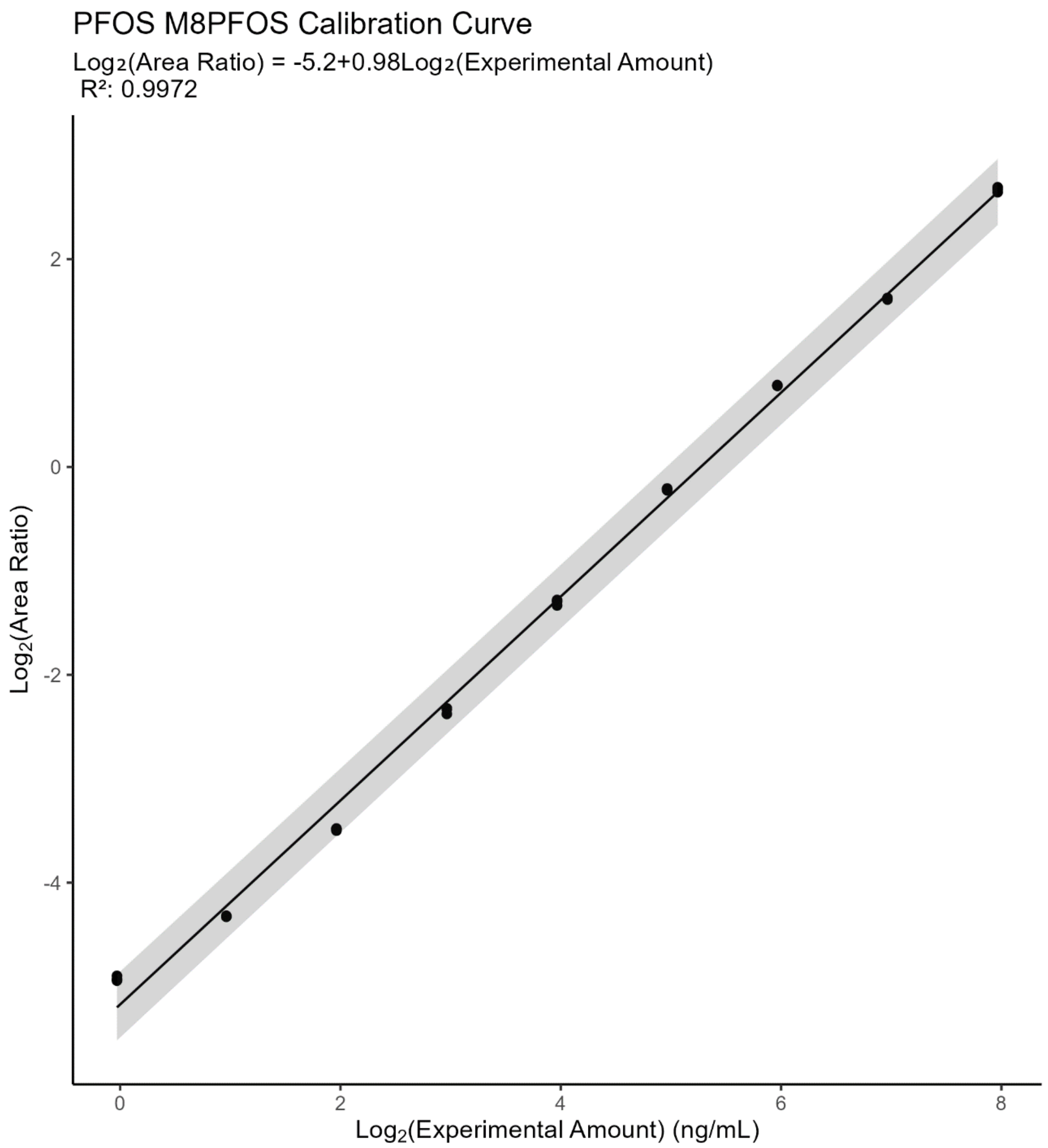

PFOS M8PFOS Calibration Curve
Log₂(Area Ratio) = -5.2+0.98Log₂(Experimental Amount)
R²: 0.9972
Log2(Area Ratio)
Log2(Experimental Amount) (ng/mL)

PFOSA M8PFOSA Calibration Curve

Log_2(Area Ratio) = -5+0.97Log_2(Experimental Amount)

R^2: 0.99172

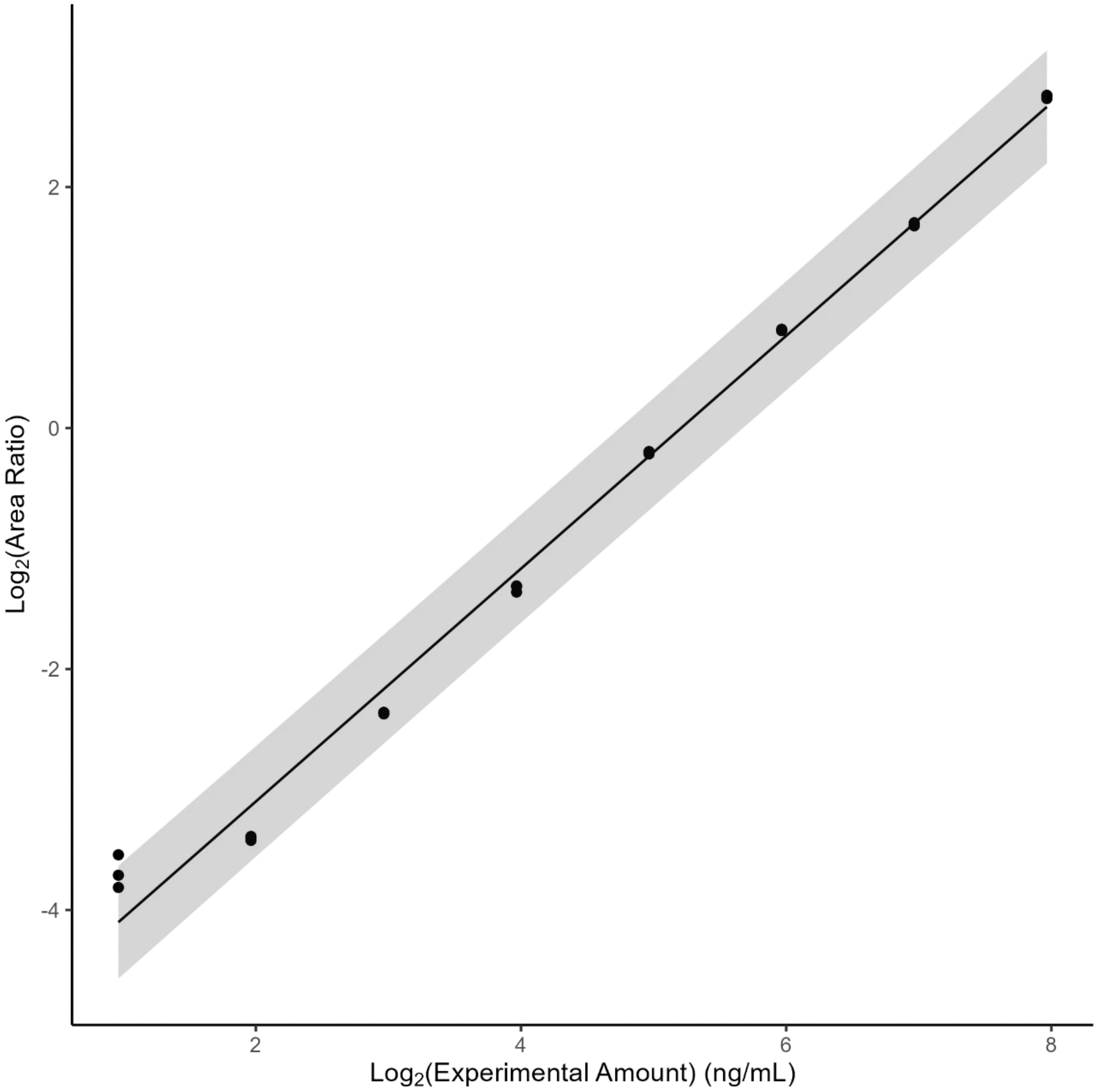

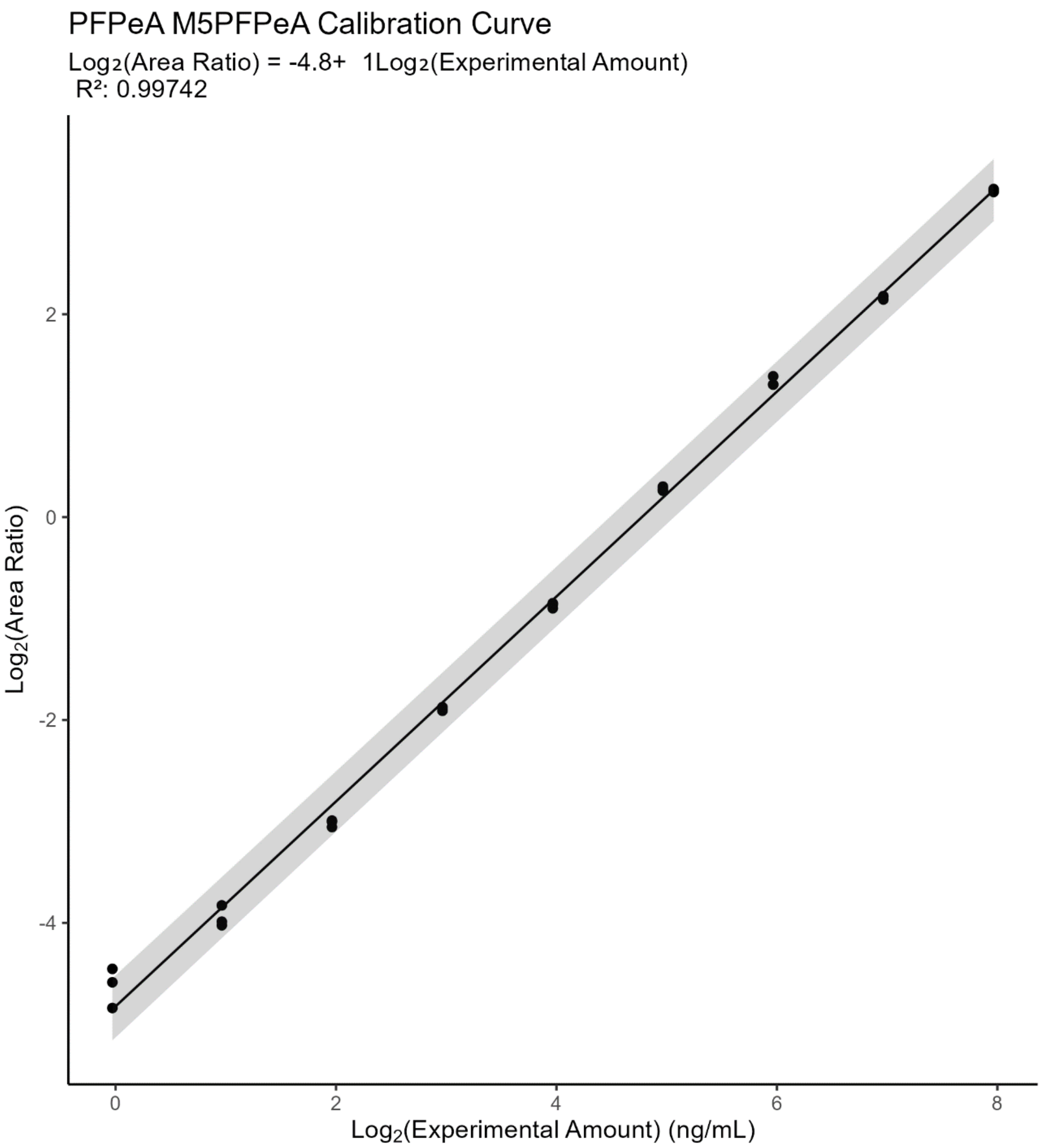
PFPeA M5PFPeA Calibration Curve
Log₂(Area Ratio) = -4.8+ 1Log₂(Experimental Amount)
R²: 0.99742
Log2(Area Ratio)
Log2(Experimental Amount) (ng/mL)
2
0
-2
-4
0
2
4
6
8

PFTeDA M2PFTeDA Calibration Curve

Log_2(Area Ratio) = -4.4+0.9Log_2(Experimental Amount)
R^2: 0.99392

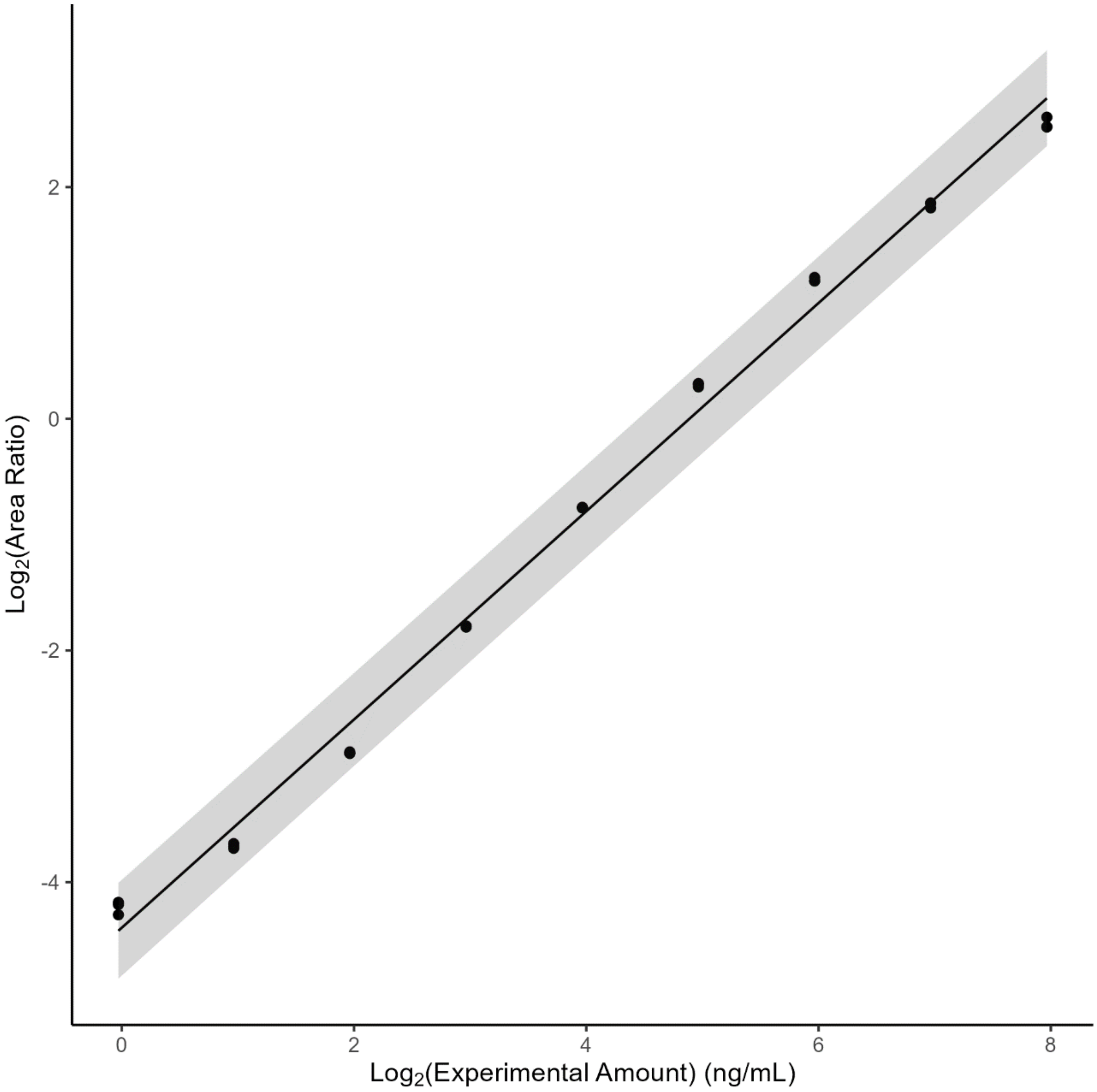

PFUnDA M7PFUnDA Calibration Curve

$\text{Log}_2(\text{Area Ratio}) = -5.4 + 1\text{Log}_2(\text{Experimental Amount})$

R^2: 0.99592

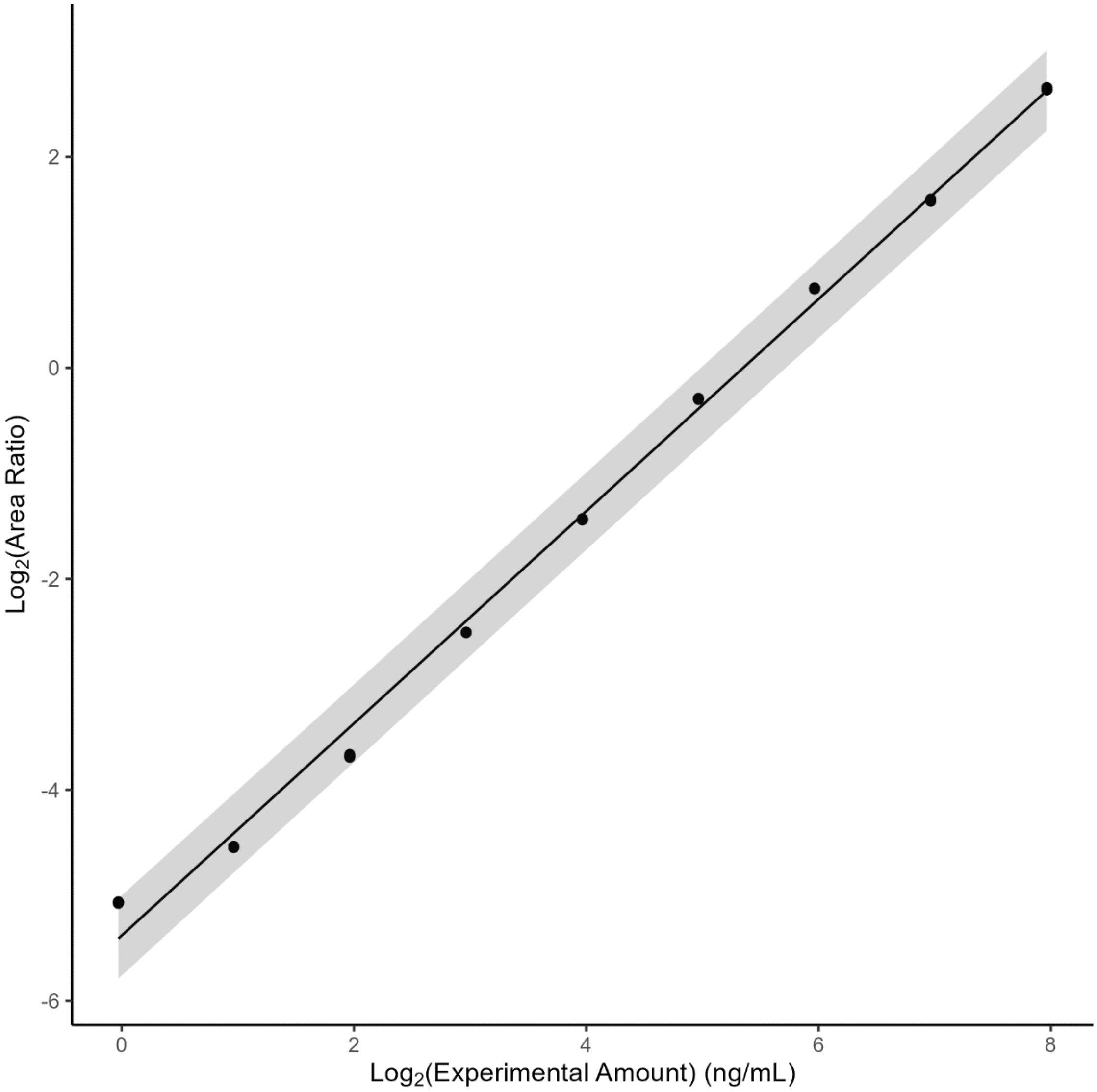

Approach 2 Calibration Curves
(n=29)

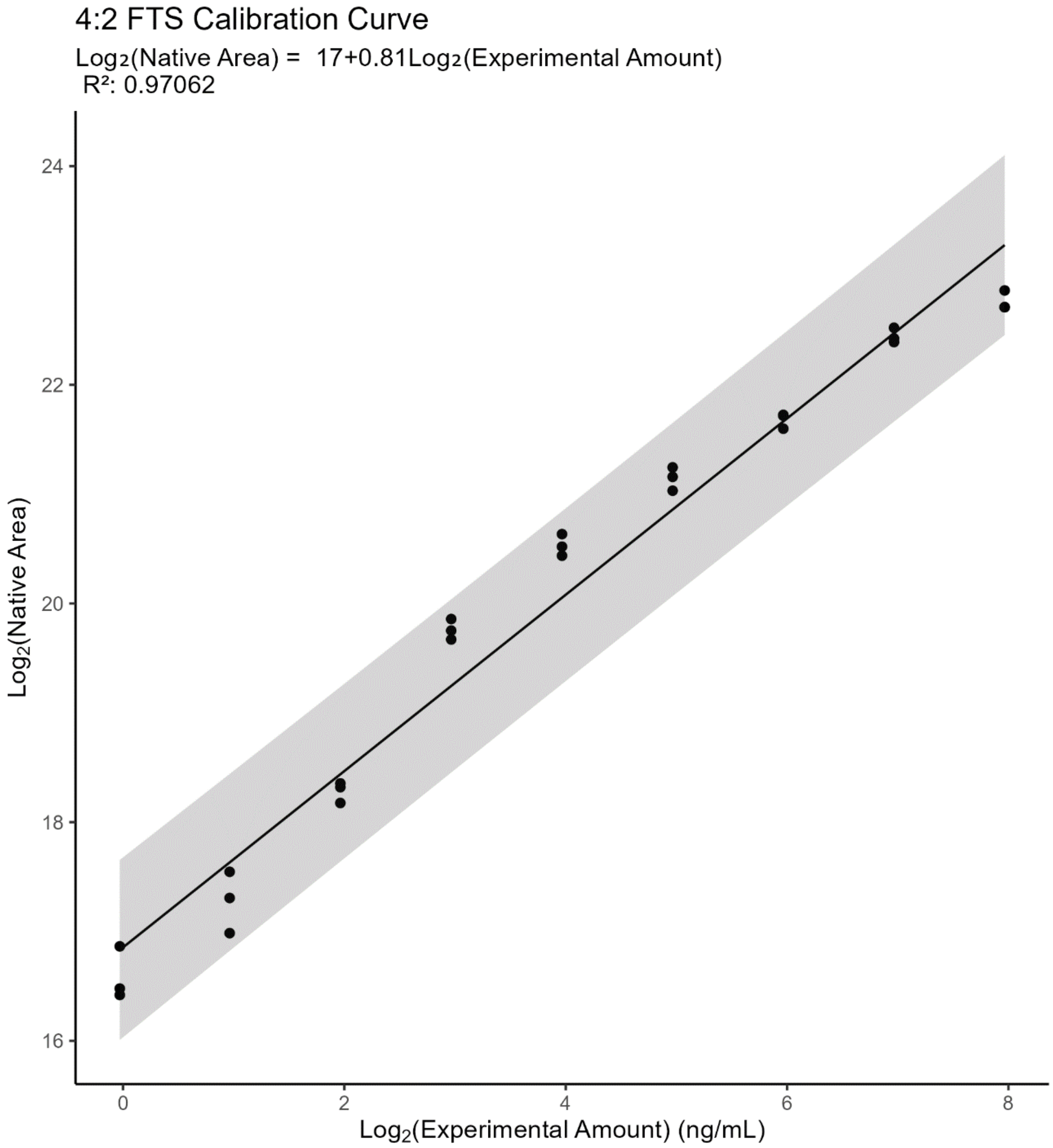
4:2 FTS Calibration Curve
Log₂(Native Area) = 17+0.81Log₂(Experimental Amount)
R²: 0.97062
Log2(Native Area)
24
22
20
18
16
0
2
4
6
8
Log2(Experimental Amount) (ng/mL)

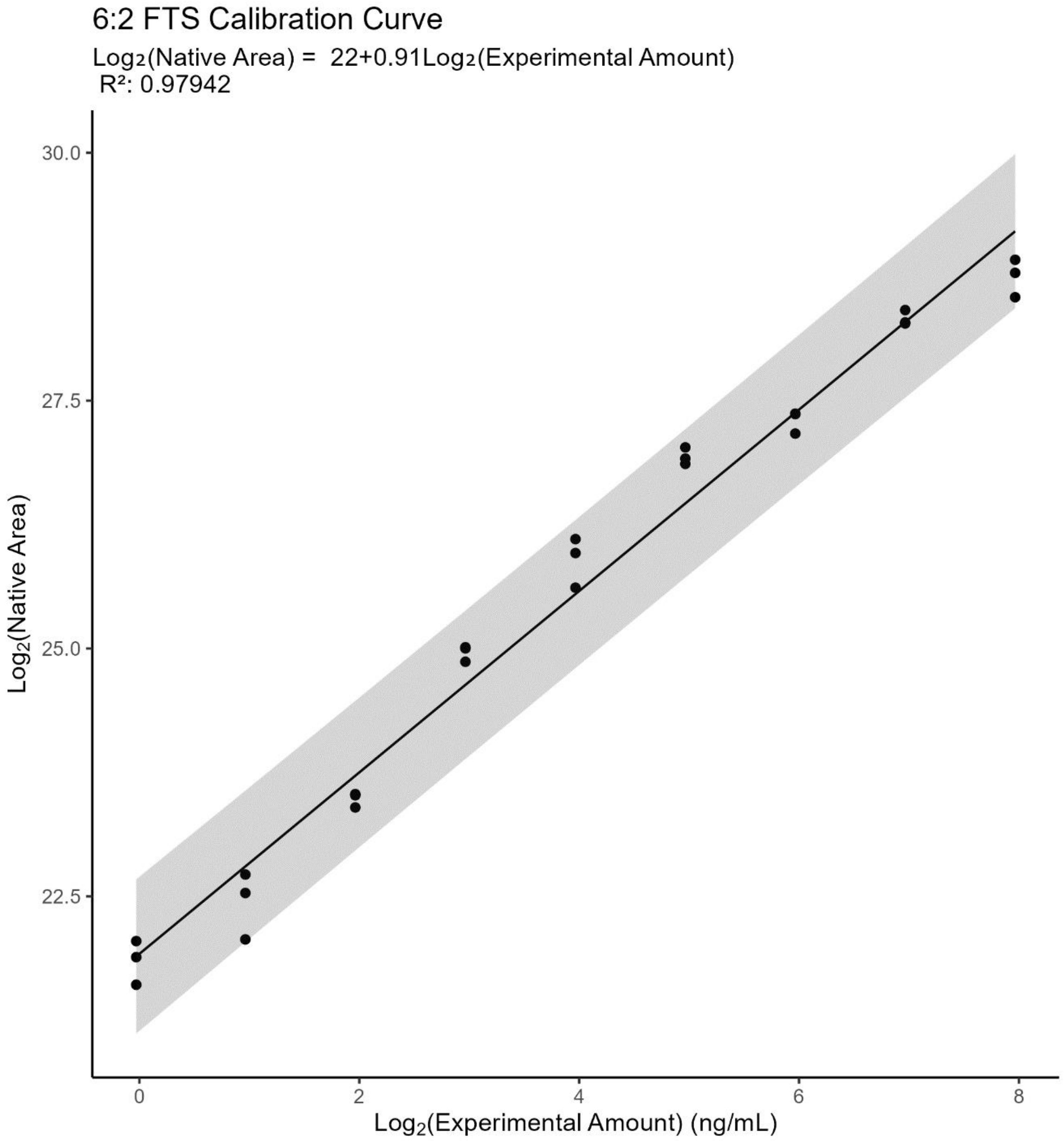
6:2 FTS Calibration Curve
Log₂(Native Area) = 22+0.91Log₂(Experimental Amount)
R²: 0.97942
30.0
27.5
25.0
22.5
Log2(Native Area)
0
2
4
6
8
Log2(Experimental Amount) (ng/mL)

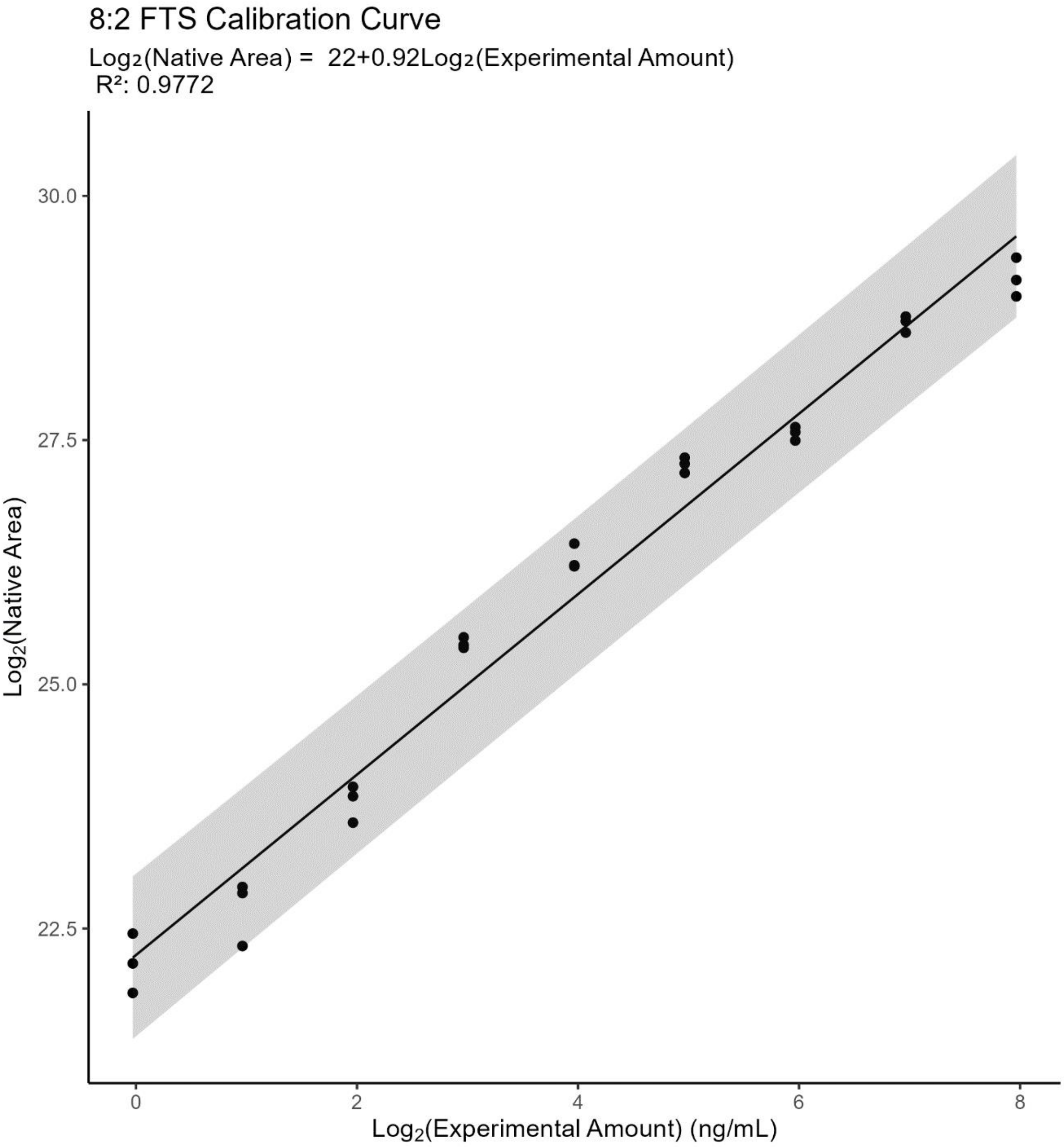
8:2 FTS Calibration Curve
Log₂(Native Area) = 22+0.92Log₂(Experimental Amount)
R²: 0.9772
30.0
27.5
25.0
22.5
0
2
4
6
8
Log₂(Native Area)
Log₂(Experimental Amount) (ng/mL)

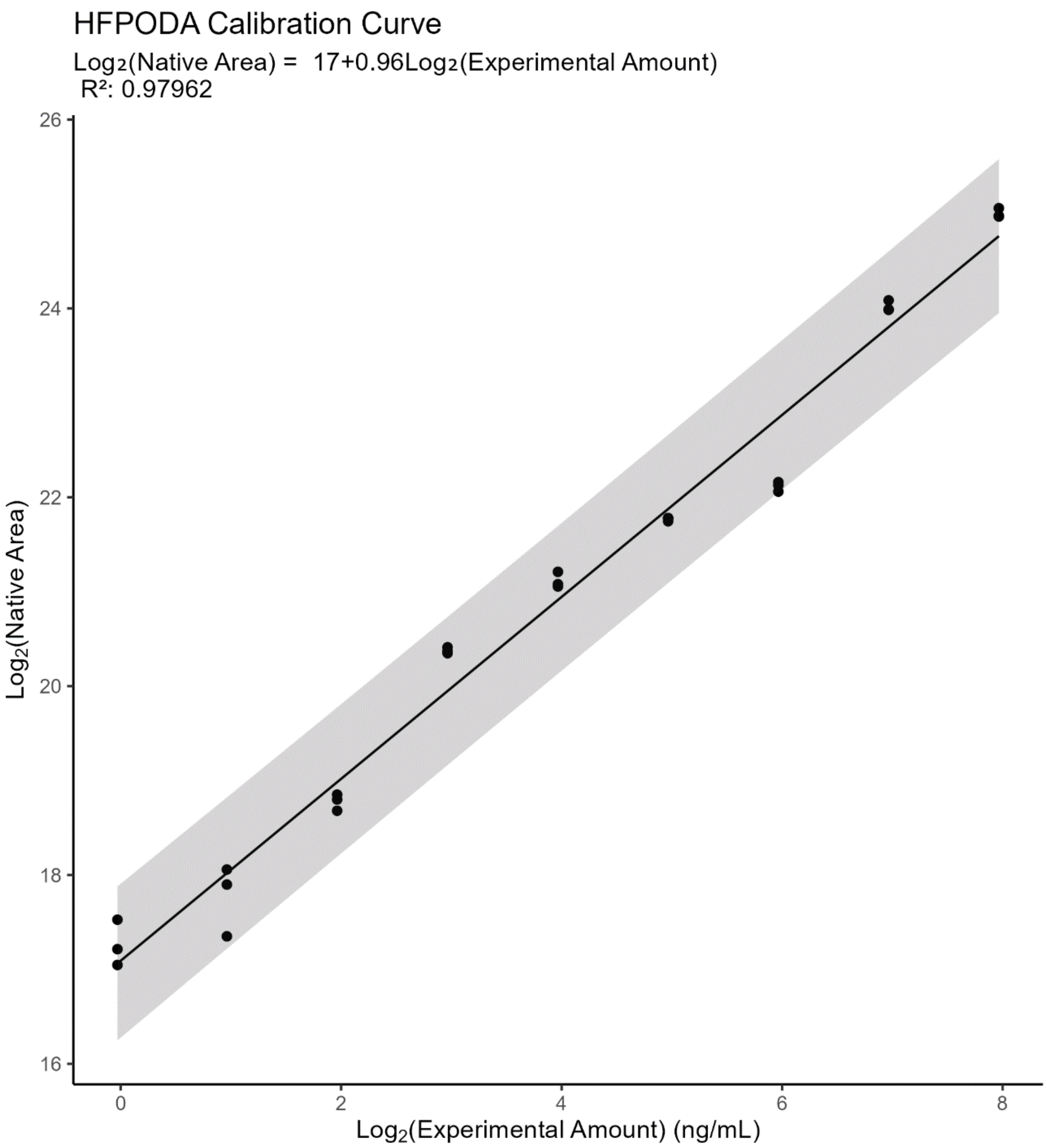

HFPODA Calibration Curve
Log₂(Native Area) = 17+0.96Log₂(Experimental Amount)
R²: 0.97962
26
24
22
20
18
16
Log₂(Native Area)
0
2
4
6
8
Log₂(Experimental Amount) (ng/mL)

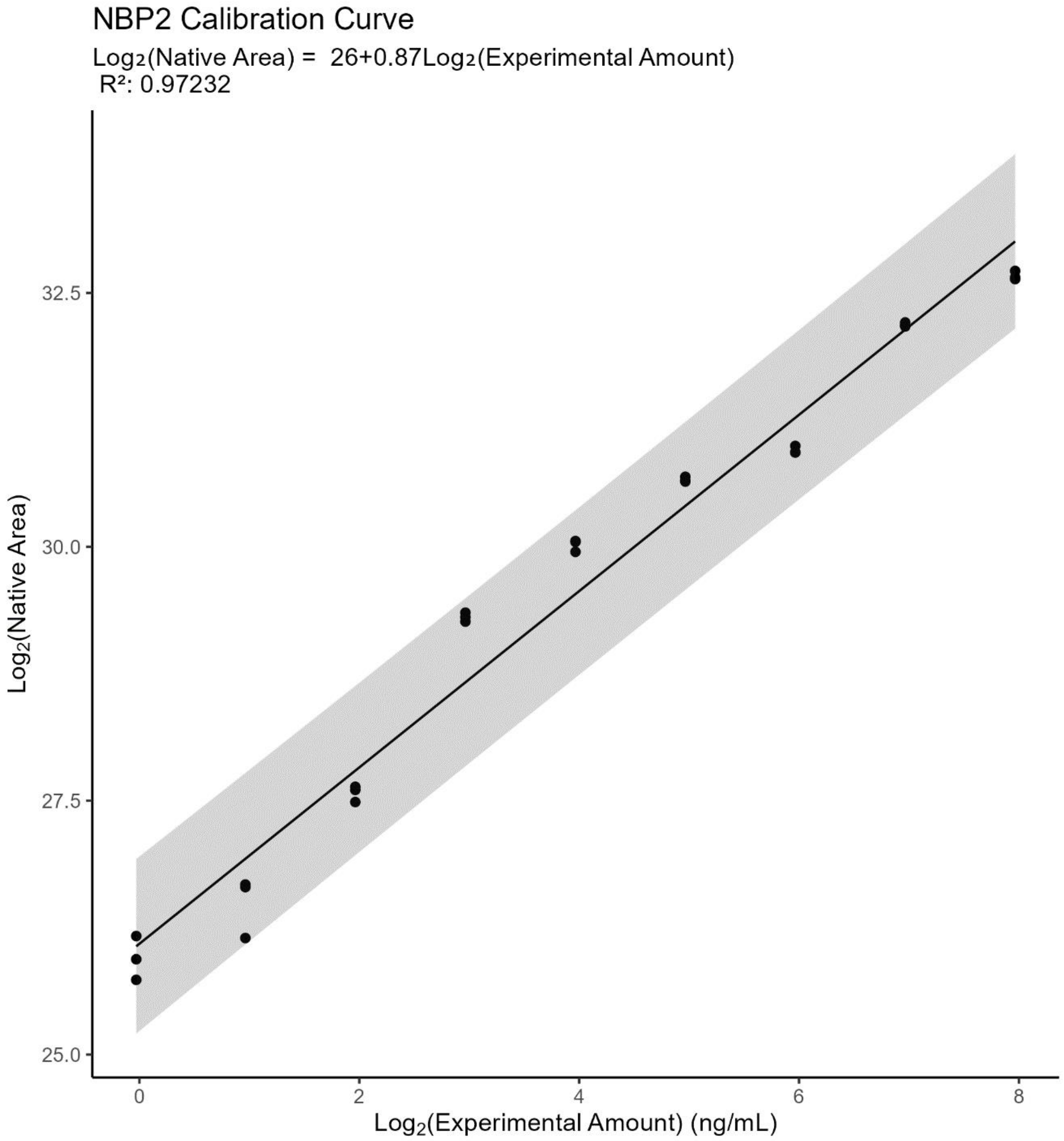

NBP2 Calibration Curve
Log₂(Native Area) = 26+0.87Log₂(Experimental Amount)
R²: 0.97232
Log2(Native Area)
32.5
30.0
27.5
25.0
0
2
4
6
8
Log2(Experimental Amount) (ng/mL)

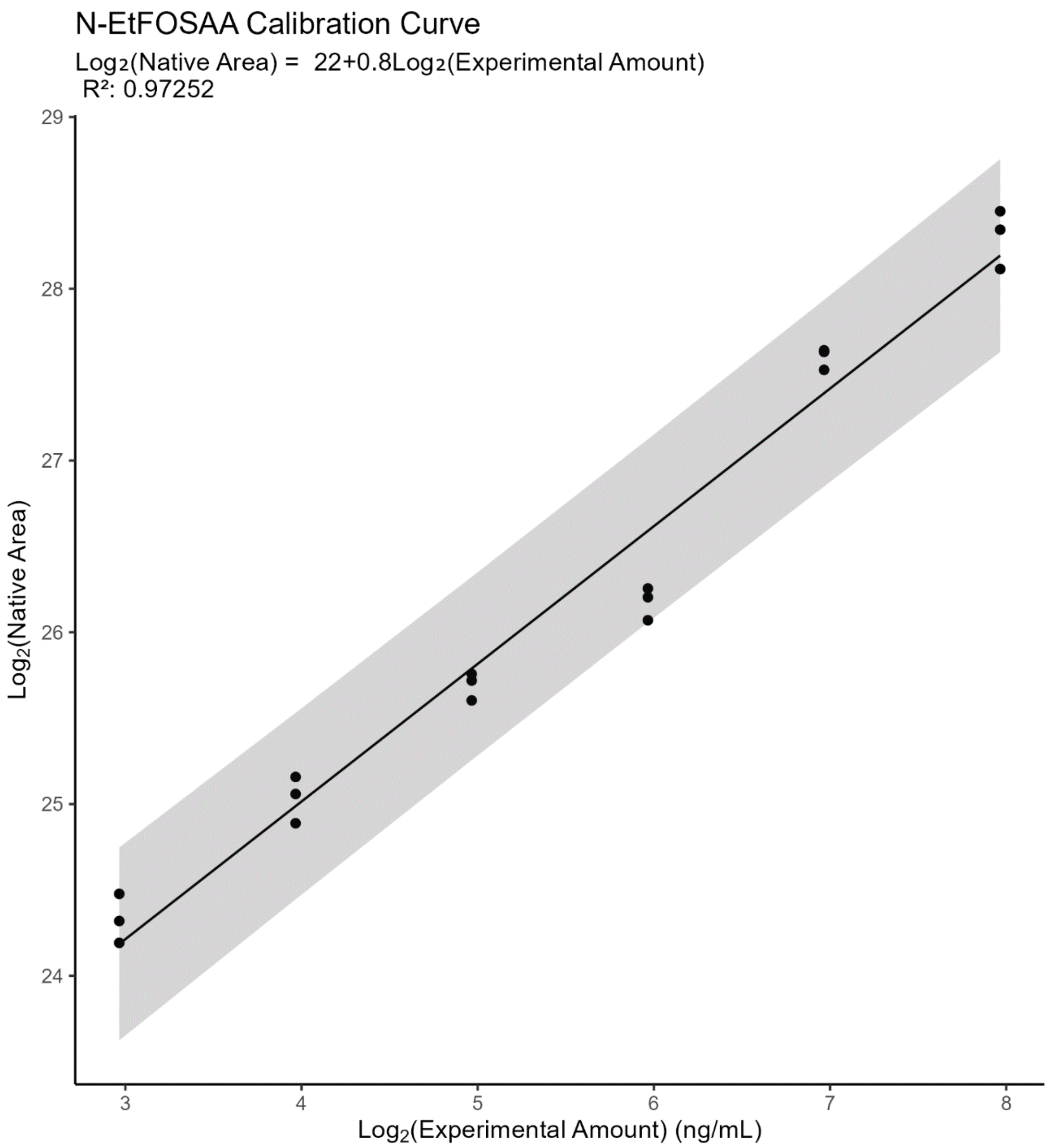
N-EtFOSAA Calibration Curve
Log₂(Native Area) = 22+0.8Log₂(Experimental Amount)
R²: 0.97252
29
28
27
26
25
24
Log2(Native Area)
3
4
5
6
7
8
Log2(Experimental Amount) (ng/mL)

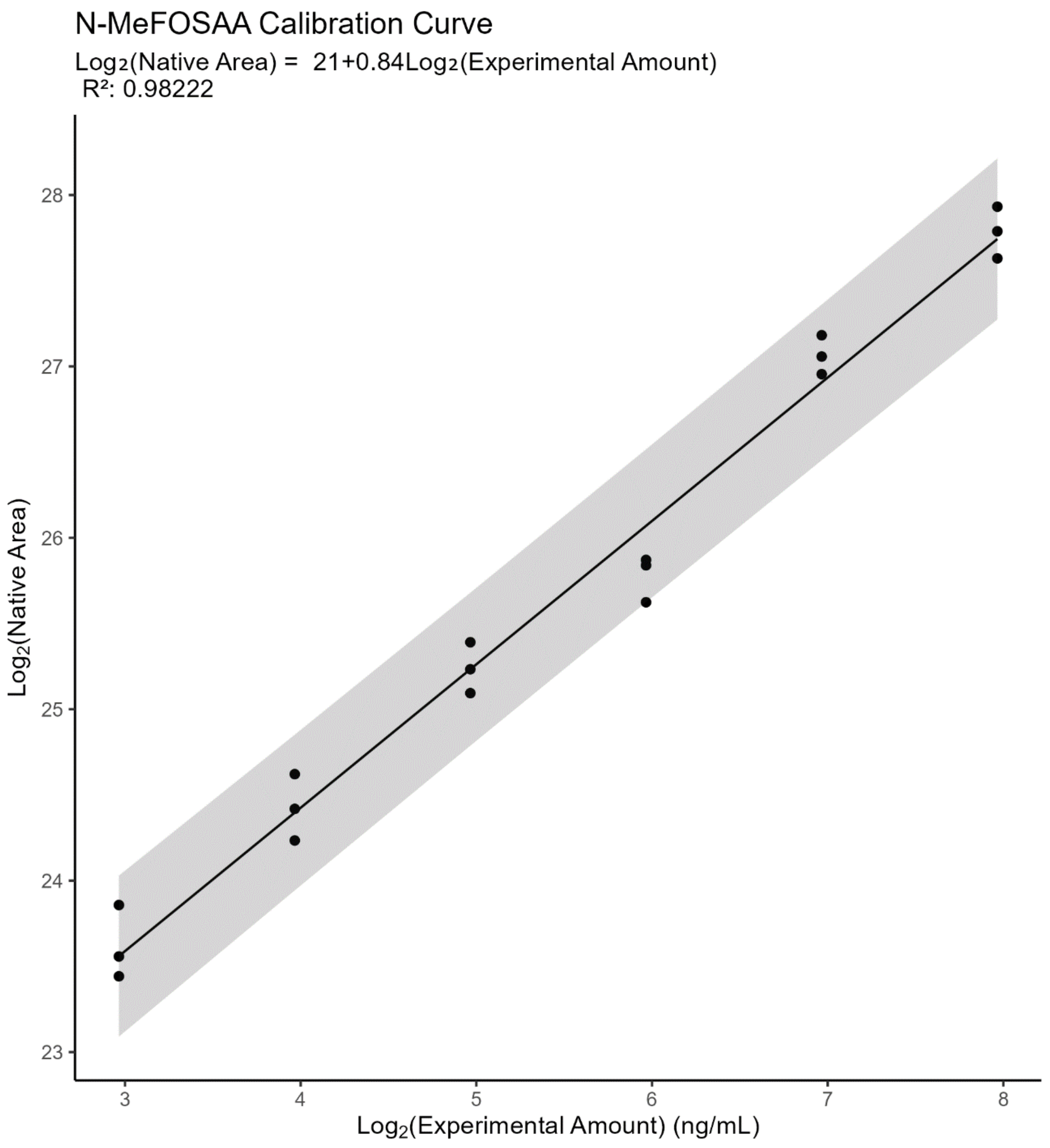
N-MeFOSAA Calibration Curve
Log₂(Native Area) = 21+0.84Log₂(Experimental Amount)
R²: 0.98222
Log2(Native Area)
28
27
26
25
24
23
3
4
5
6
7
8
Log2(Experimental Amount) (ng/mL)

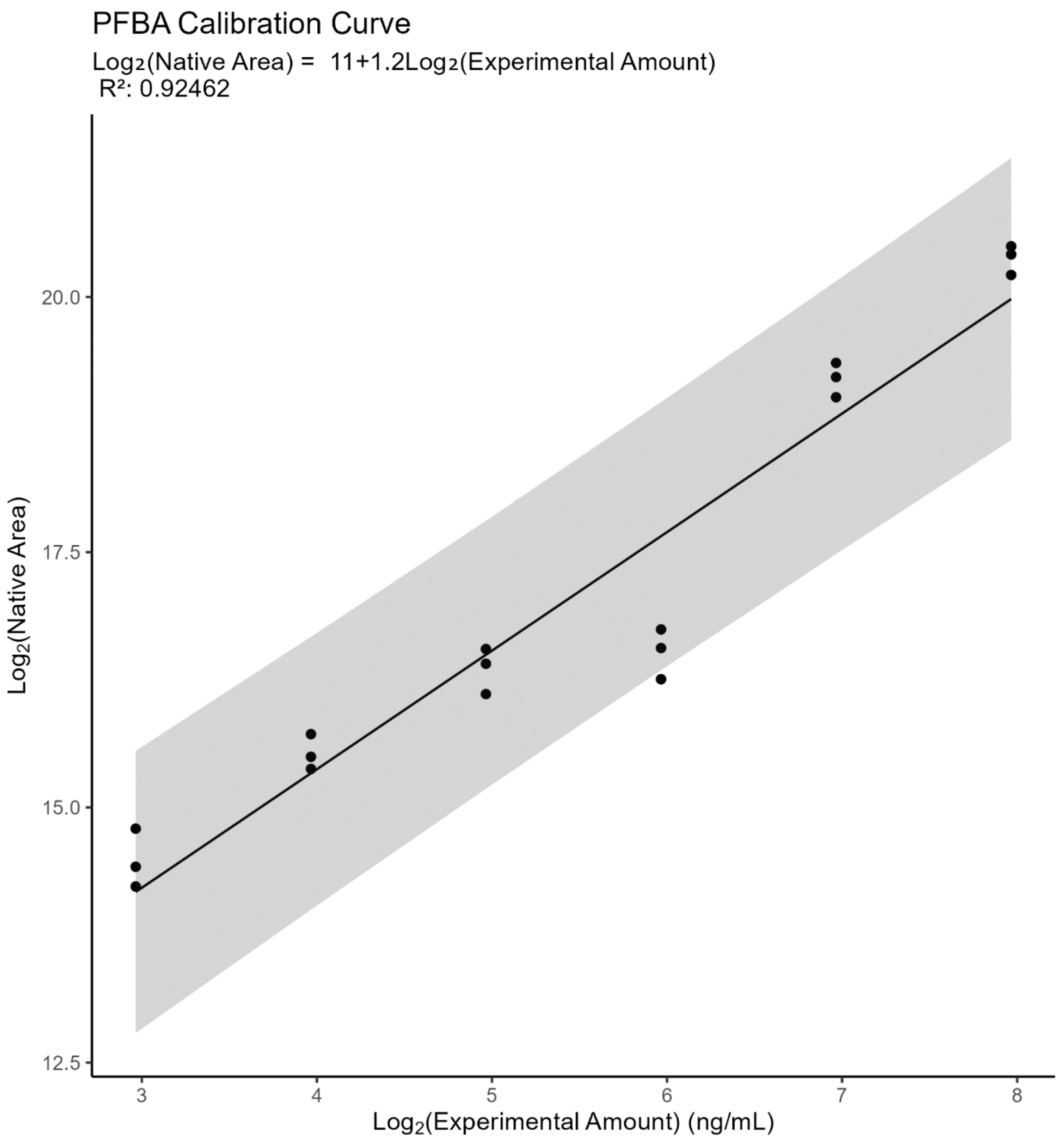

PFBA Calibration Curve
Log₂(Native Area) = 11+1.2Log₂(Experimental Amount)
R²: 0.92462
Log2(Native Area)
20.0
17.5
15.0
12.5
3
4
5
6
7
8
Log2(Experimental Amount) (ng/mL)

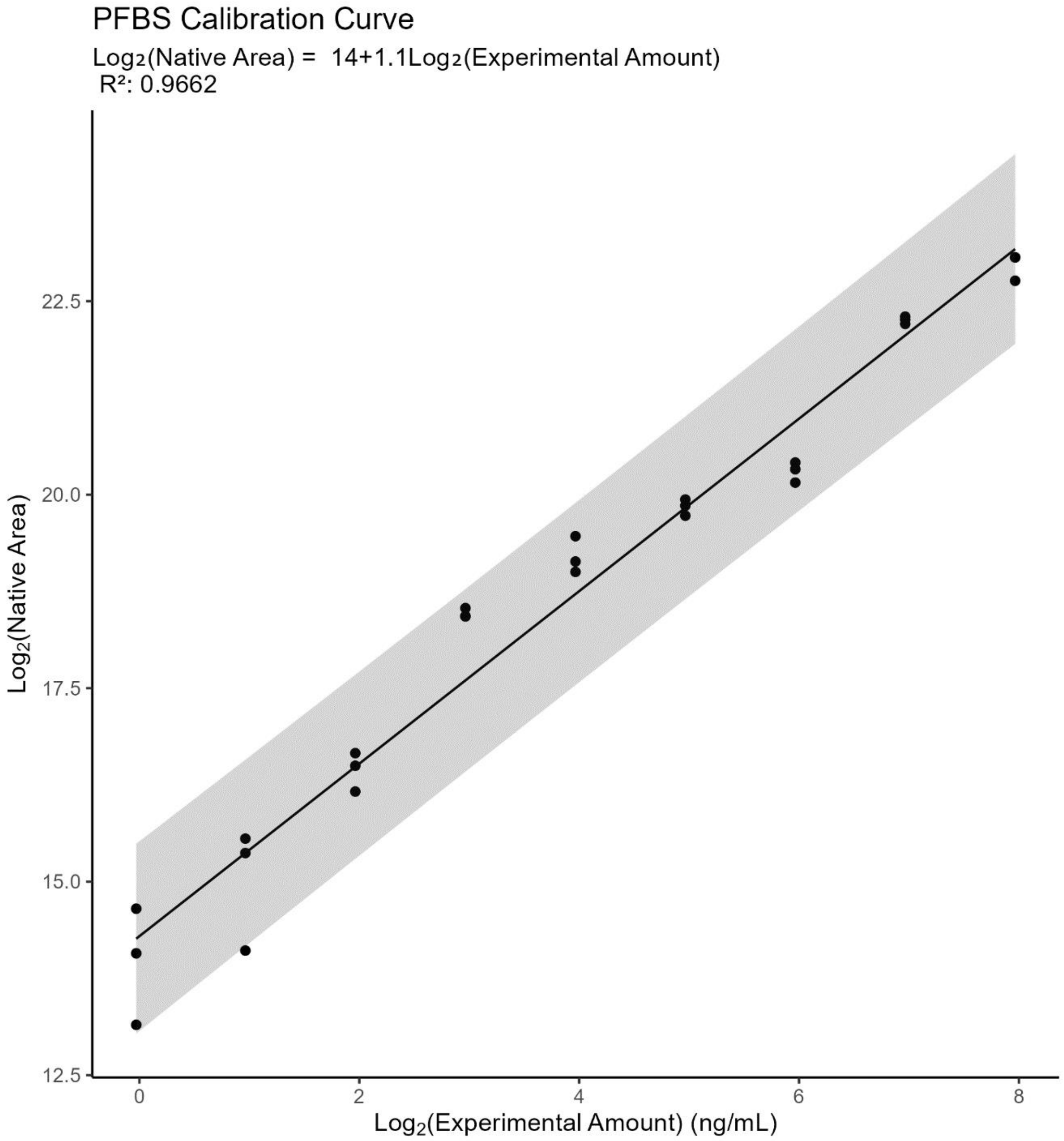
PFBS Calibration Curve
Log₂(Native Area) = 14+1.1Log₂(Experimental Amount)
R²: 0.9662
Log2(Native Area)
22.5
20.0
17.5
15.0
12.5
0
2
4
6
8
Log2(Experimental Amount) (ng/mL)

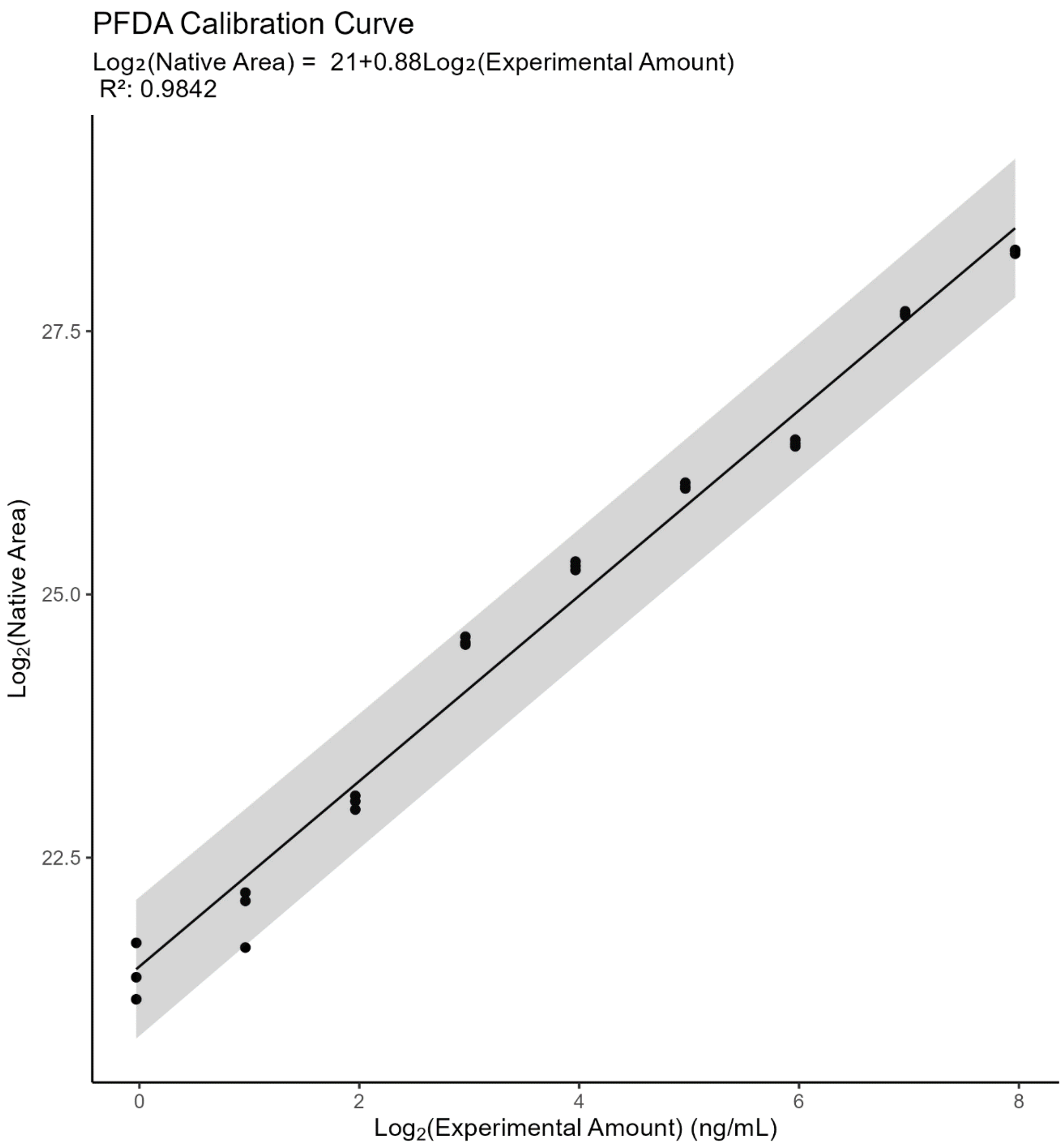

PFDA Calibration Curve
Log₂(Native Area) = 21+0.88Log₂(Experimental Amount)
R²: 0.9842
27.5
25.0
22.5
Log2(Native Area)
0
2
4
6
8
Log2(Experimental Amount) (ng/mL)

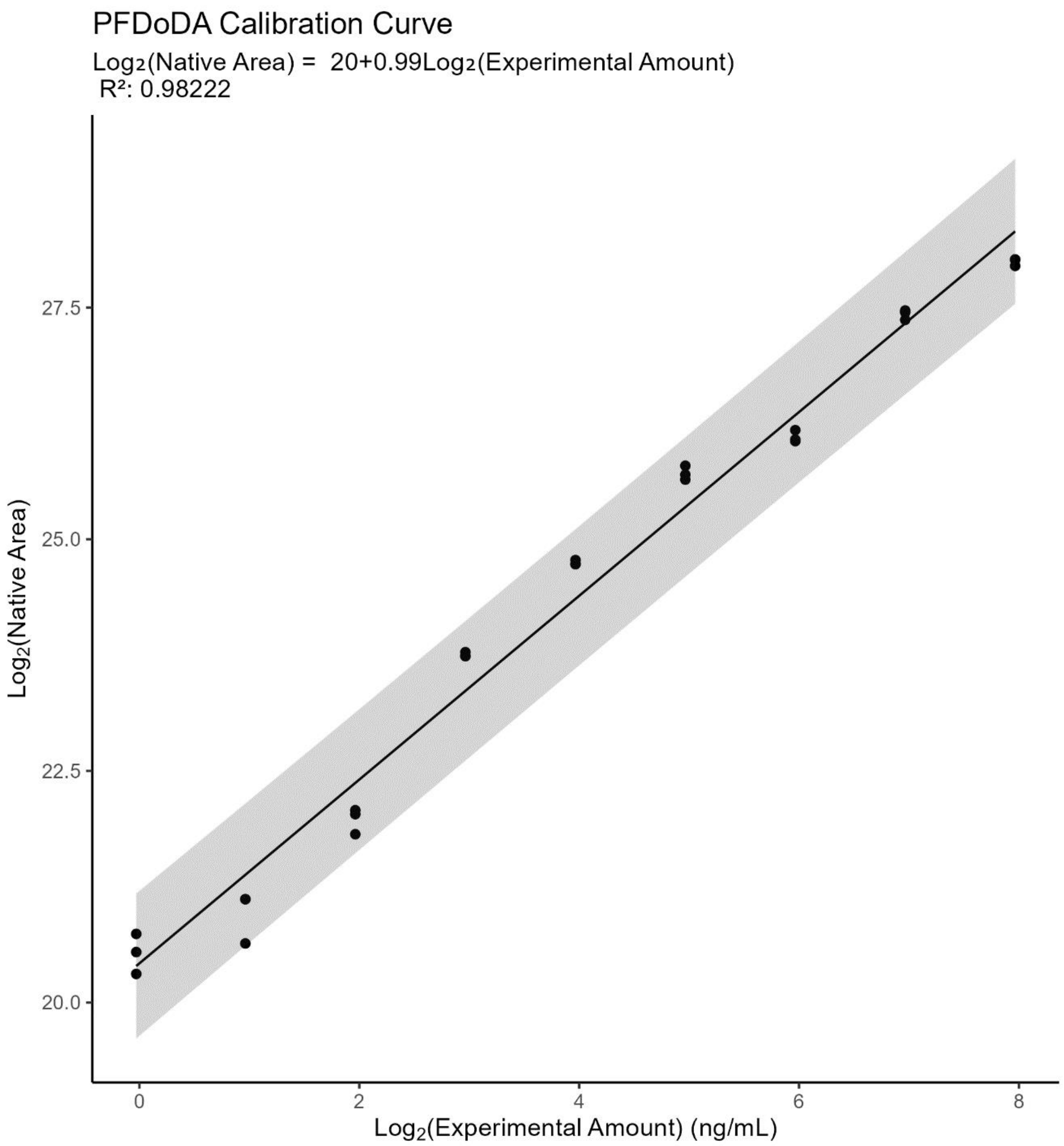
PFDoDA Calibration Curve
Log₂(Native Area) = 20+0.99Log₂(Experimental Amount)
R²: 0.98222
27.5
25.0
22.5
20.0
Log2(Native Area)
0
2
4
6
8
Log2(Experimental Amount) (ng/mL)

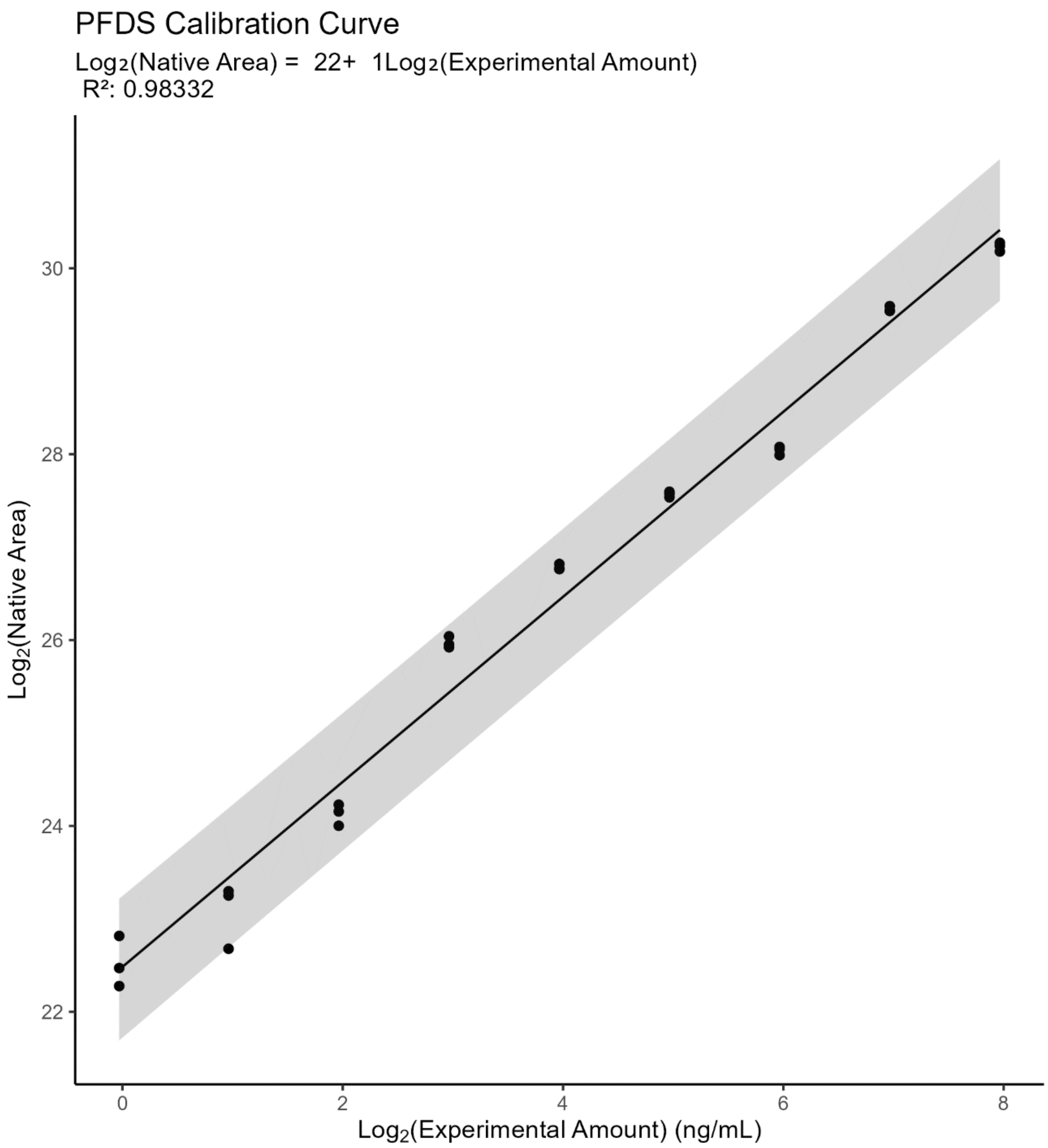
PFDS Calibration Curve
Log₂(Native Area) = 22+ 1Log₂(Experimental Amount)
R²: 0.98332
Log2(Native Area)
30
28
26
24
22
0
2
4
6
8
Log2(Experimental Amount) (ng/mL)

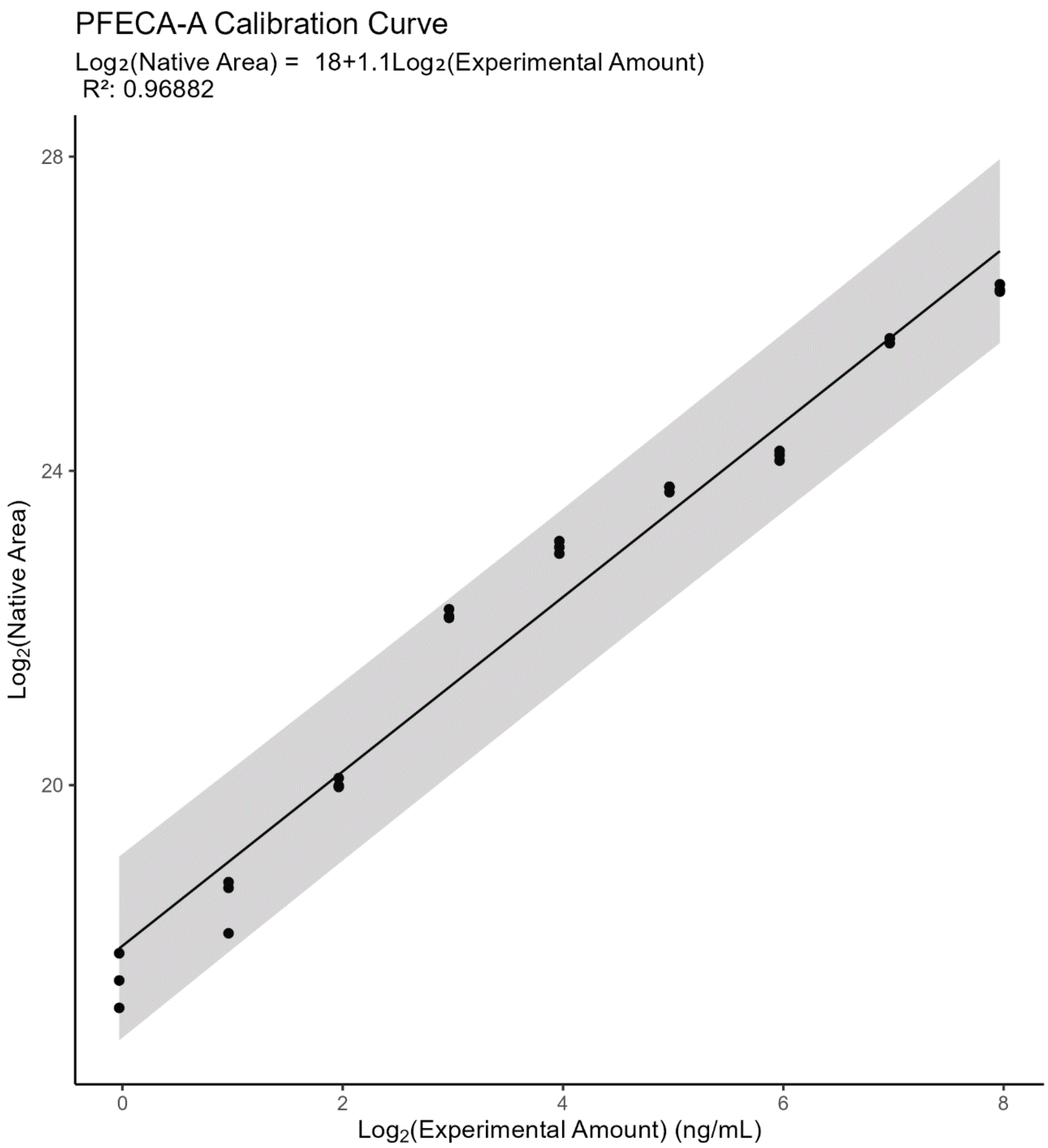
PFECA-A Calibration Curve
Log₂(Native Area) = 18+1.1Log₂(Experimental Amount)
R²: 0.96882
28
24
20
Log₂(Native Area)
0
2
4
6
8
Log₂(Experimental Amount) (ng/mL)

PFECA-F Calibration Curve

Log_2(Native Area) = 12+1.4Log_2(Experimental Amount)

R^2: 0.96882

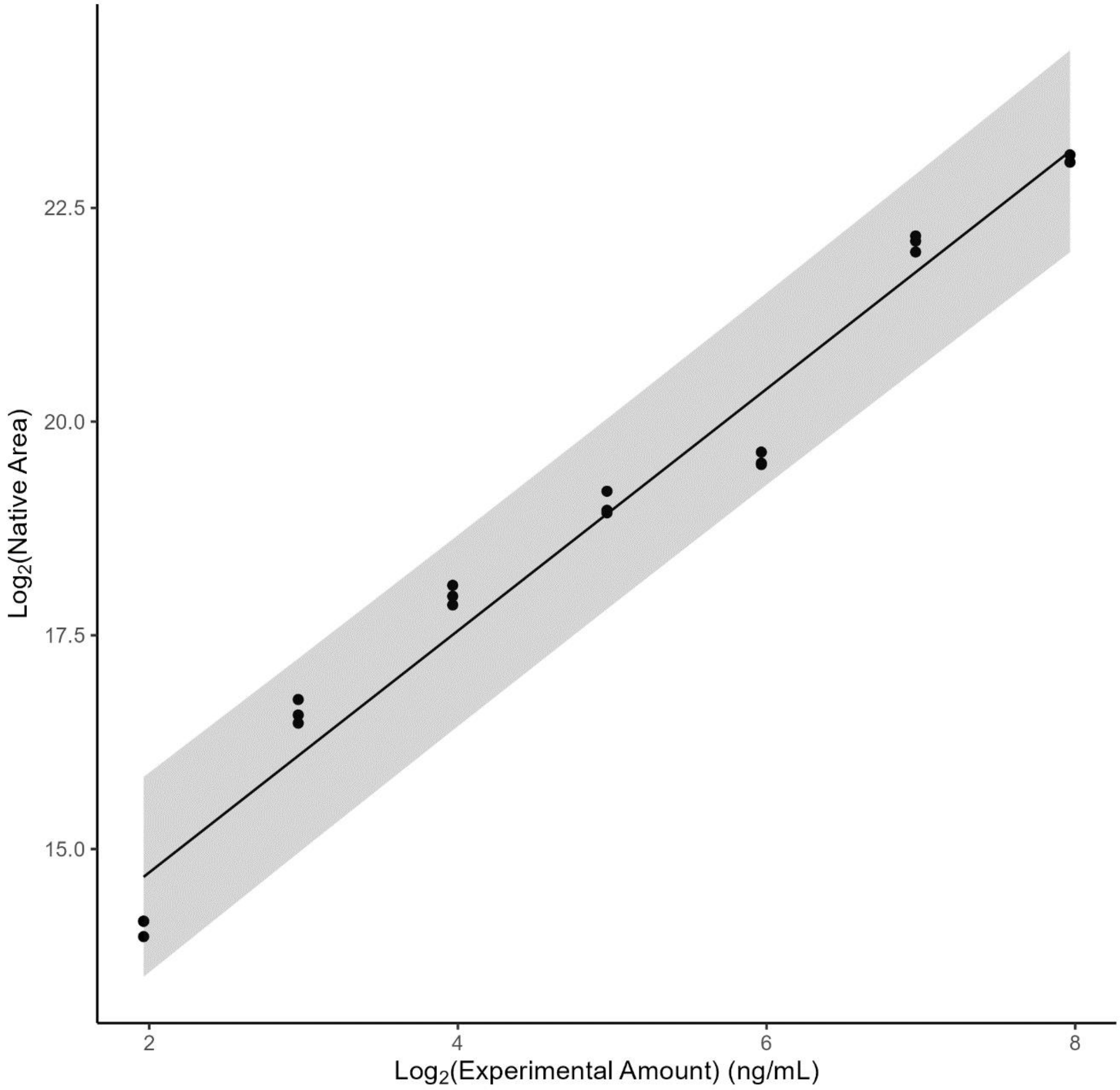

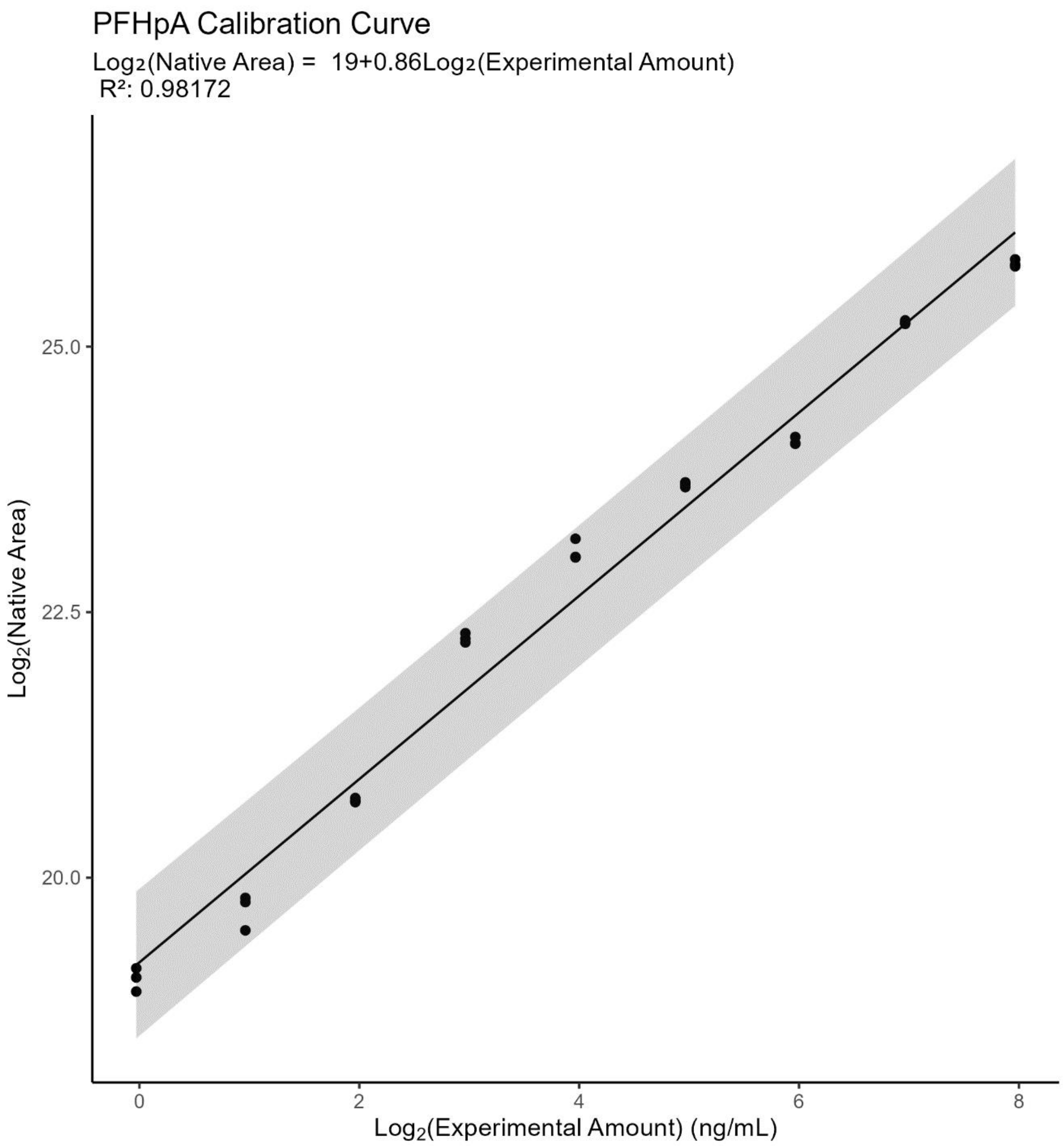
PFHpA Calibration Curve
Log₂(Native Area) = 19+0.86Log₂(Experimental Amount)
R²: 0.98172
25.0
22.5
20.0
Log2(Native Area)
0
2
4
6
8
Log2(Experimental Amount) (ng/mL)

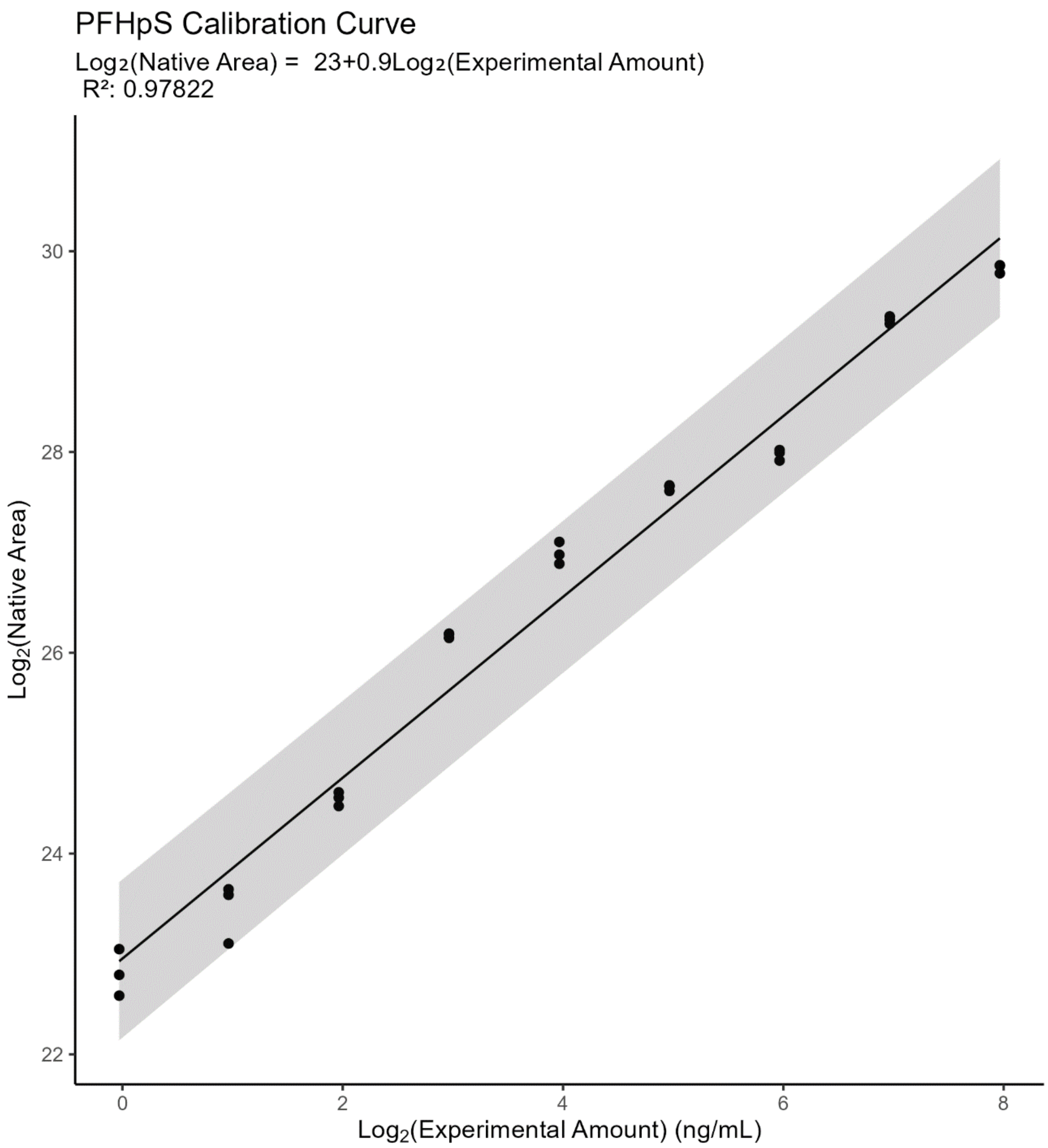
PFHpS Calibration Curve
Log2(Native Area) = 23+0.9Log2(Experimental Amount)
R²: 0.97822
30
28
26
24
22
Log2(Native Area)
0
2
4
6
8
Log2(Experimental Amount) (ng/mL)

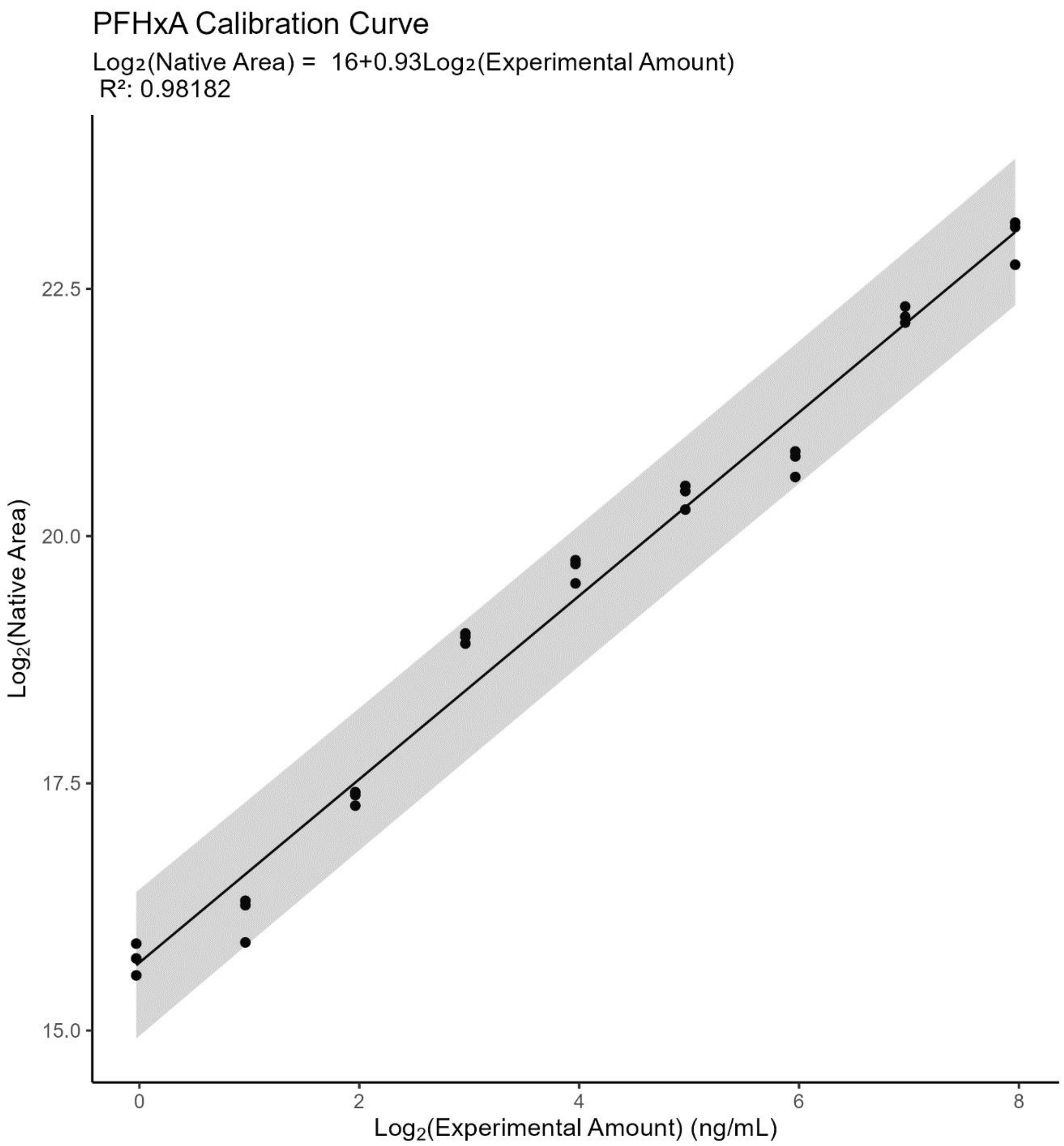

PFHxA Calibration Curve
Log2(Native Area) = 16+0.93Log2(Experimental Amount)
R²: 0.98182
Log2(Native Area)
22.5
20.0
17.5
15.0
0
2
4
6
8
Log2(Experimental Amount) (ng/mL)

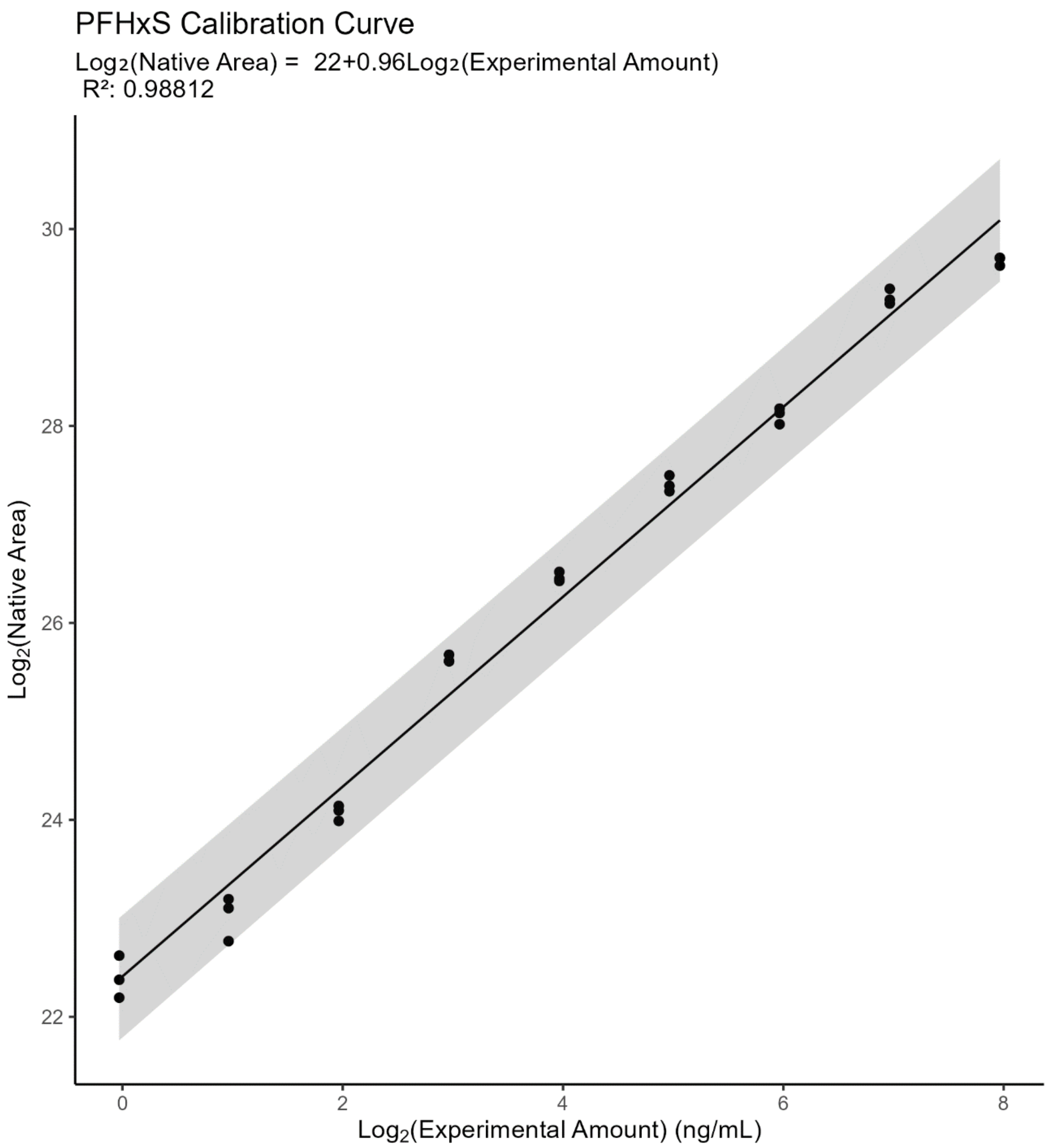

PFHxS Calibration Curve
Log₂(Native Area) = 22+0.96Log₂(Experimental Amount)
R²: 0.98812
Log2(Native Area)
30
28
26
24
22
0
2
4
6
8
Log2(Experimental Amount) (ng/mL)

PFMOAA Calibration Curve

$\text{Log}_2(\text{Native Area}) = 6.2+1.9\text{Log}_2(\text{Experimental Amount})$

R^2: 0.95982

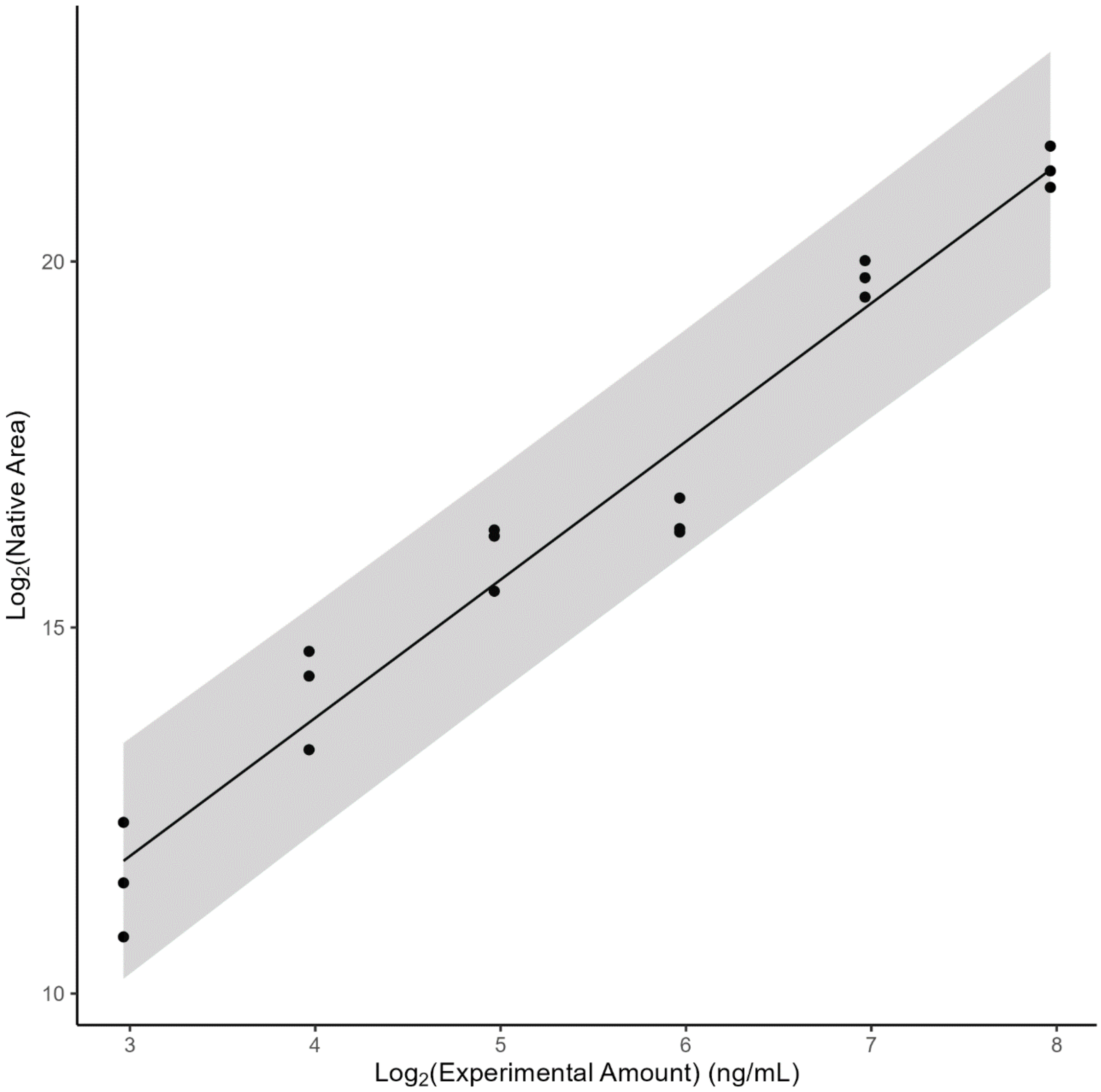

PFNA Calibration Curve

Log2(Native Area) = 21+0.77Log2(Experimental Amount)

R^2: 0.96182

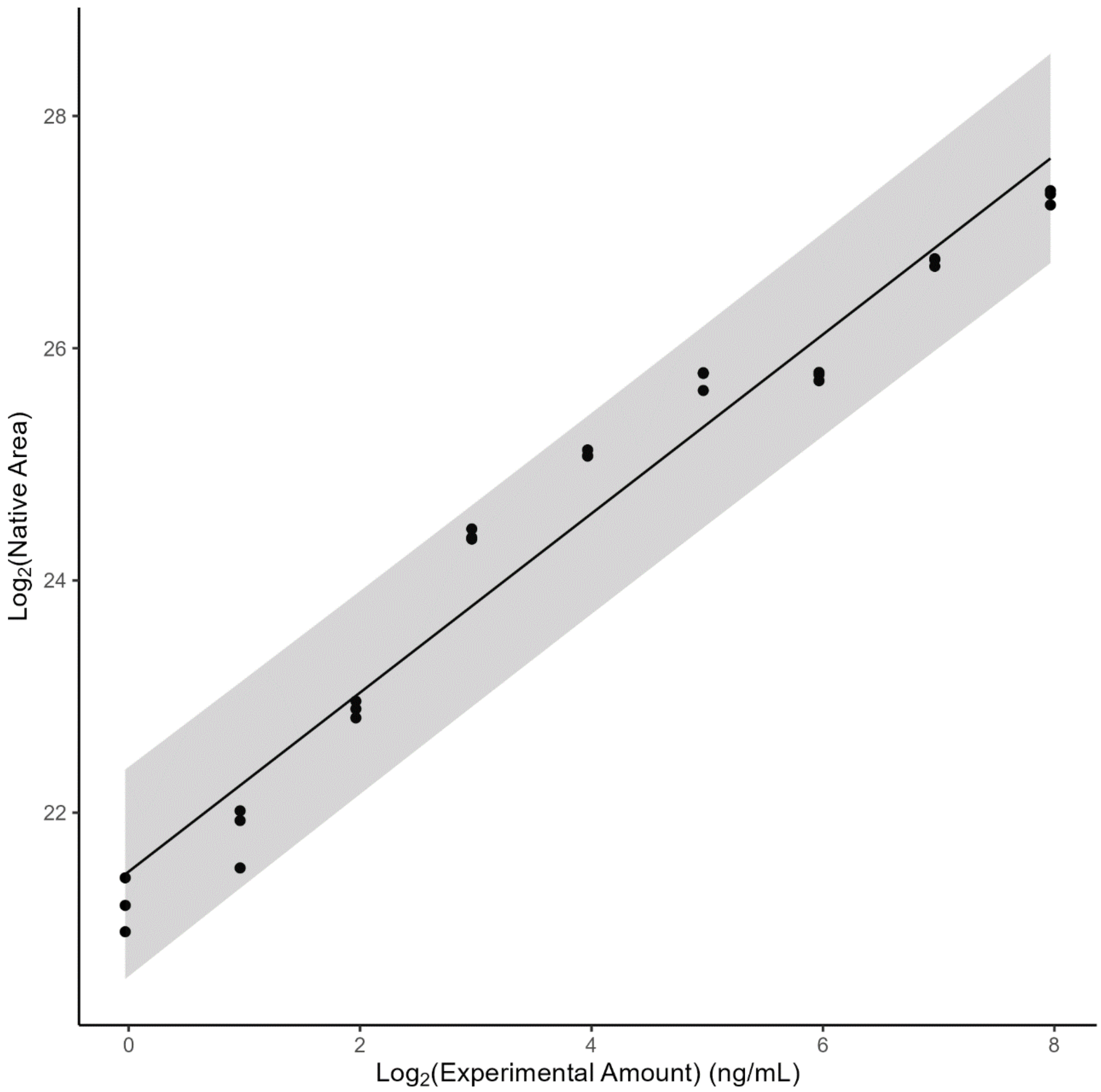

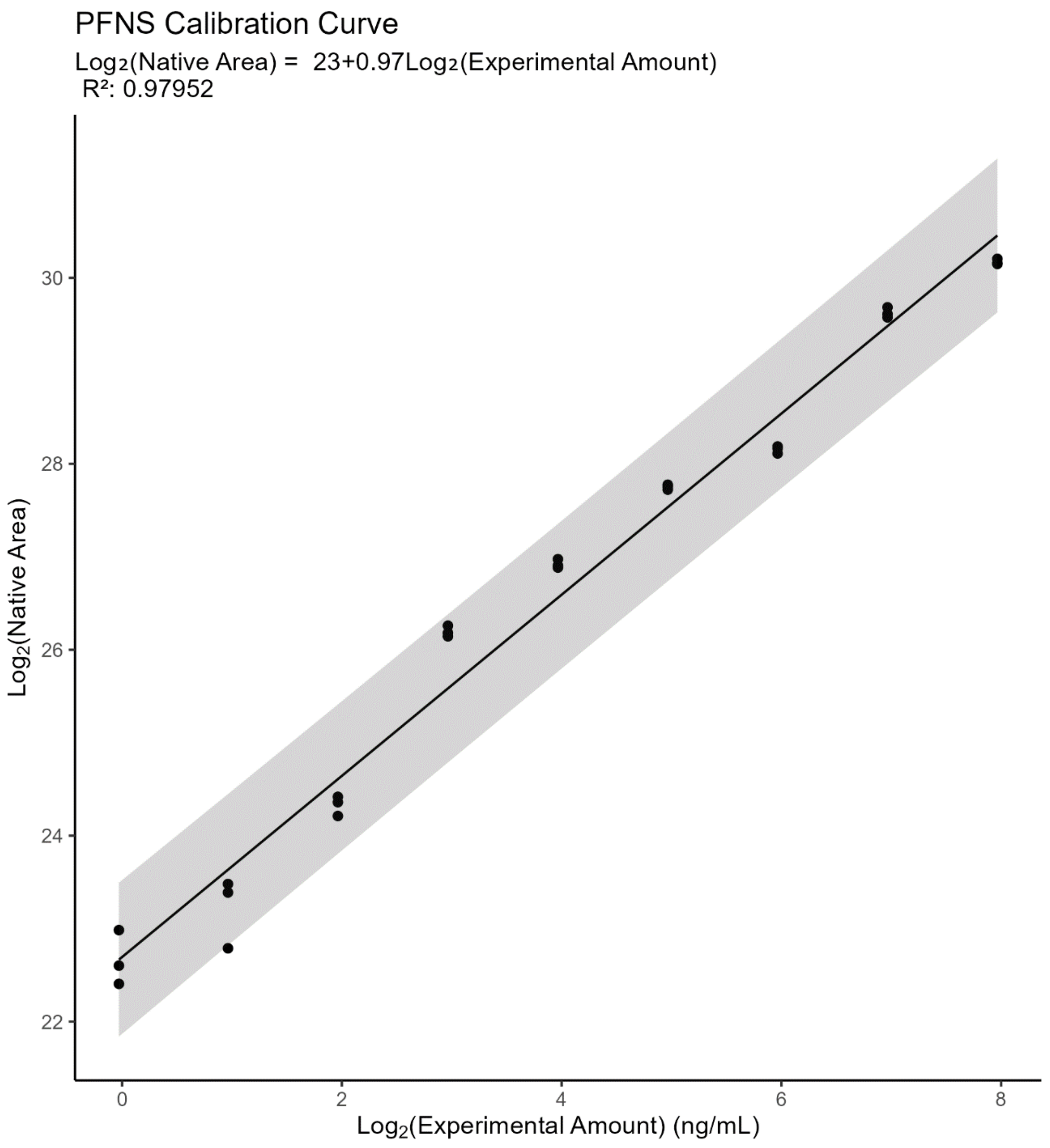
PFNS Calibration Curve
Log₂(Native Area) = 23+0.97Log₂(Experimental Amount)
R²: 0.97952
Log2(Native Area)
30
28
26
24
22
0
2
4
6
8
Log2(Experimental Amount) (ng/mL)

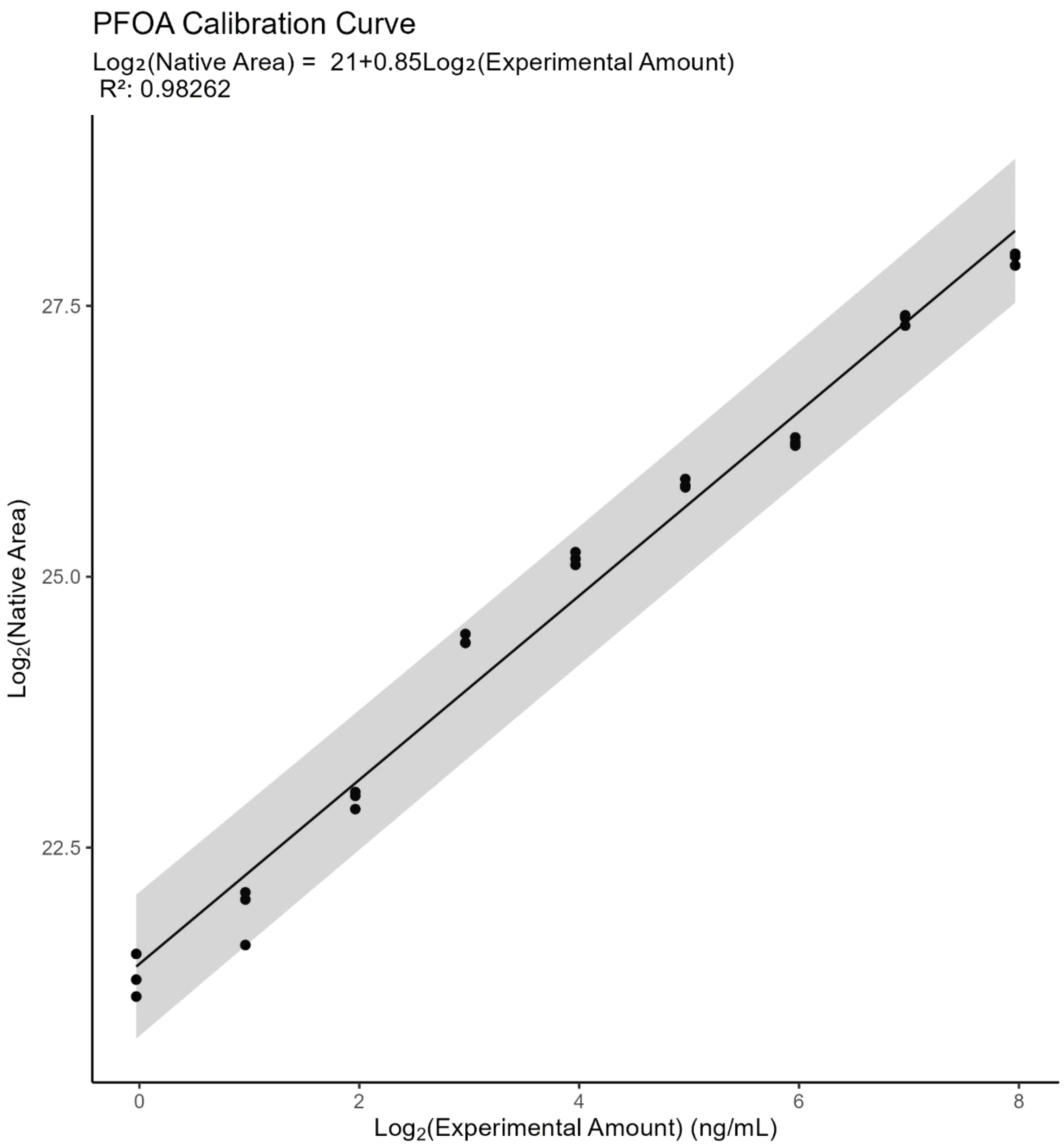
PFOA Calibration Curve
Log₂(Native Area) = 21+0.85Log₂(Experimental Amount)
R²: 0.98262
Log₂(Native Area)
27.5
25.0
22.5
0
2
4
6
8
Log₂(Experimental Amount) (ng/mL)

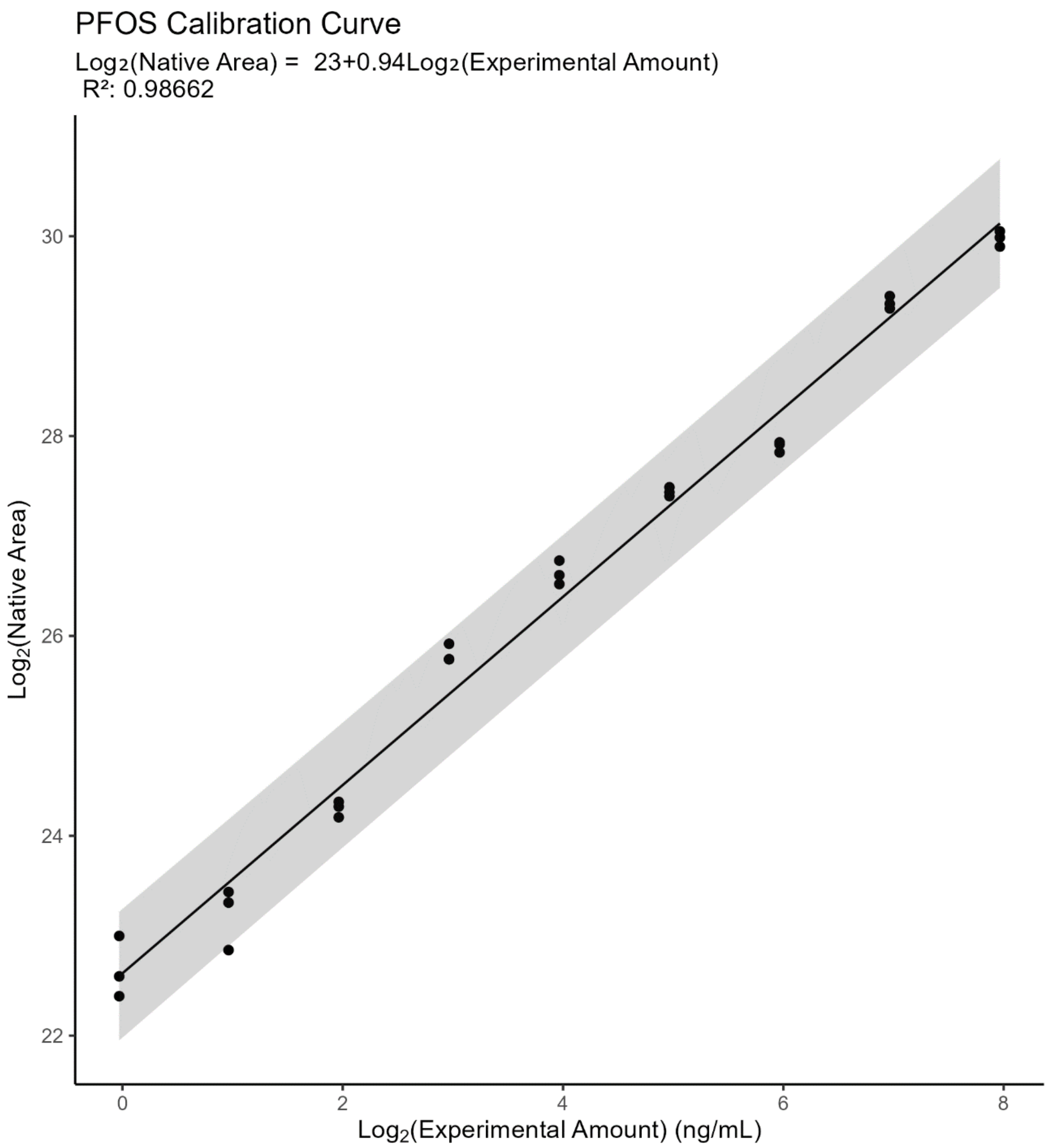
PFOS Calibration Curve
Log₂(Native Area) = 23+0.94Log₂(Experimental Amount)
R²: 0.98662
Log2(Native Area)
Log2(Experimental Amount) (ng/mL)
22
24
26
28
30
0
2
4
6
8

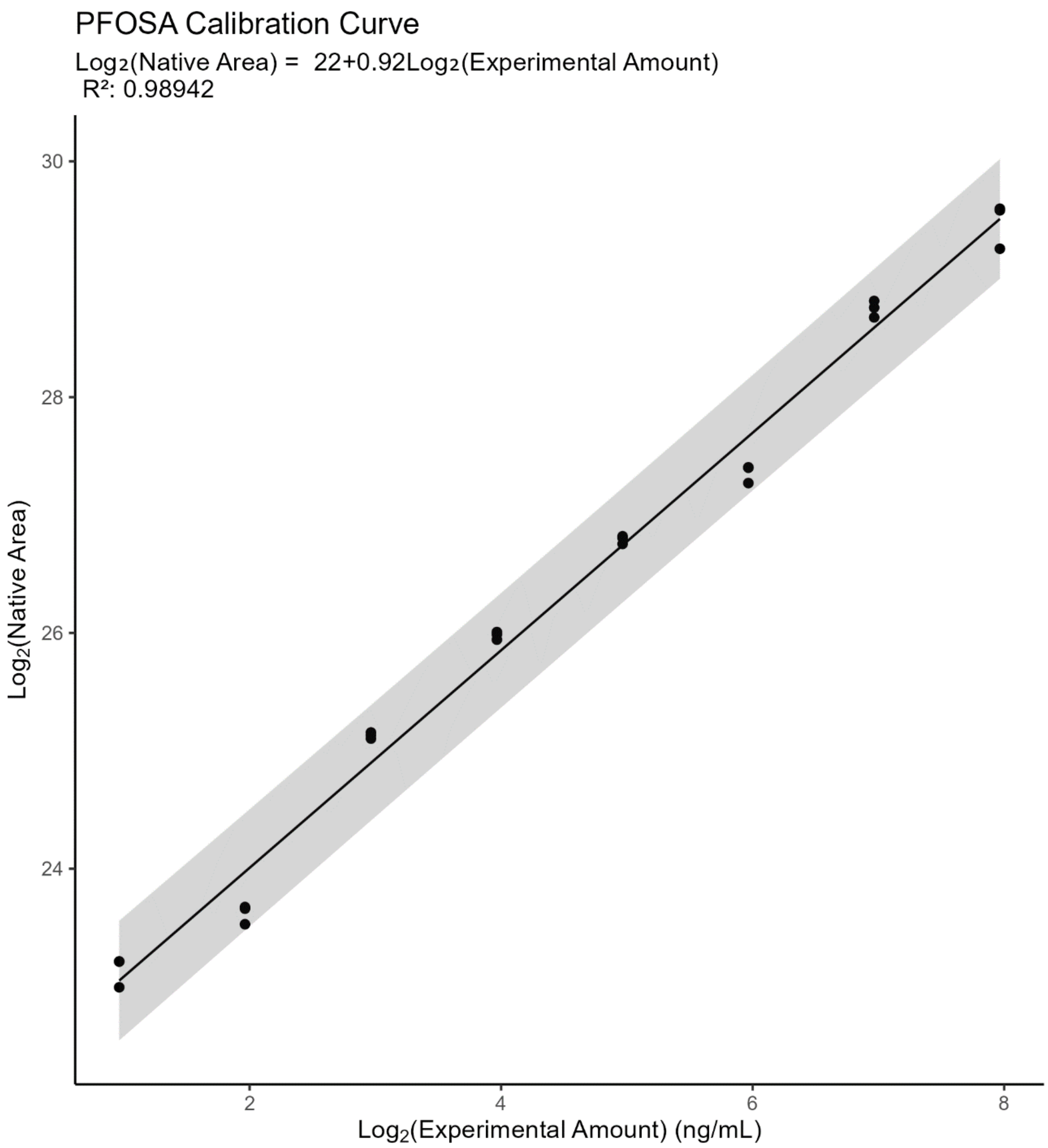
PFOSA Calibration Curve
Log₂(Native Area) = 22+0.92Log₂(Experimental Amount)
R²: 0.98942
Log2(Native Area)
30
28
26
24
2
4
6
8
Log2(Experimental Amount) (ng/mL)

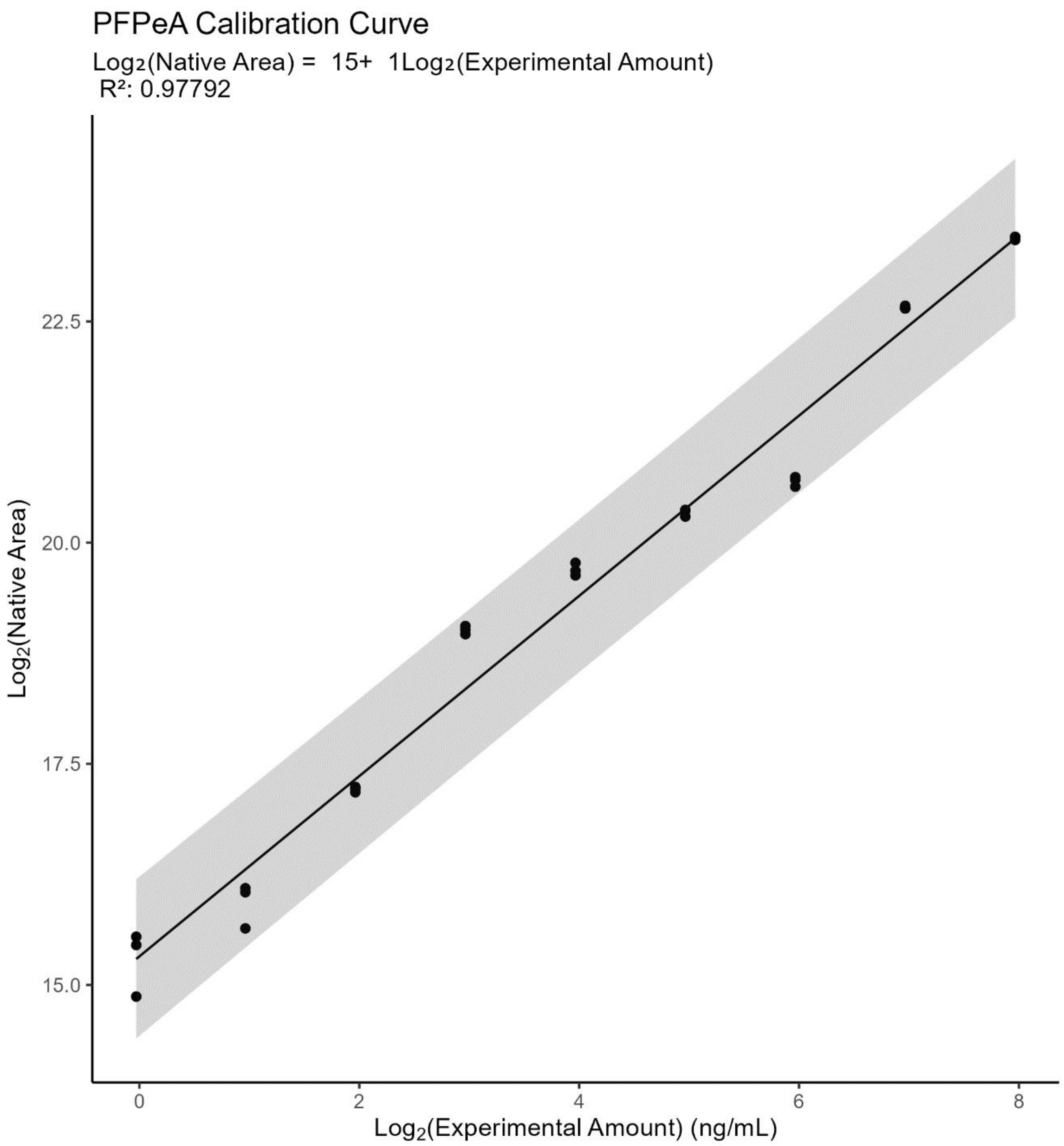

PFPeA Calibration Curve
Log₂(Native Area) = 15+ 1Log₂(Experimental Amount)
R²: 0.97792
22.5
20.0
17.5
15.0
Log2(Native Area)
0
2
4
6
8
Log2(Experimental Amount) (ng/mL)

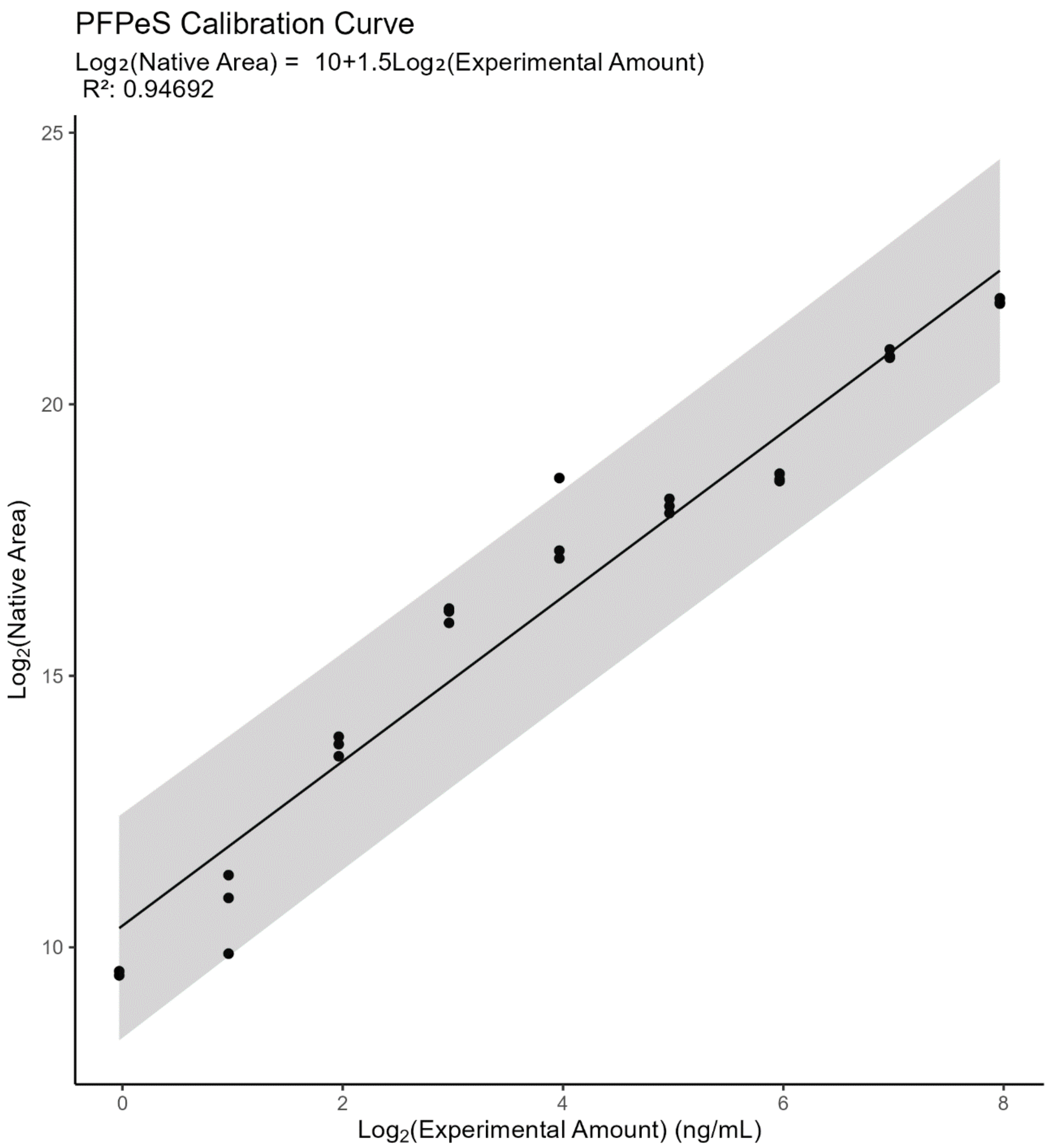

PFPeS Calibration Curve
Log₂(Native Area) = 10+1.5Log₂(Experimental Amount)
R²: 0.94692
25
20
15
10
Log₂(Native Area)
0
2
4
6
8
Log₂(Experimental Amount) (ng/mL)

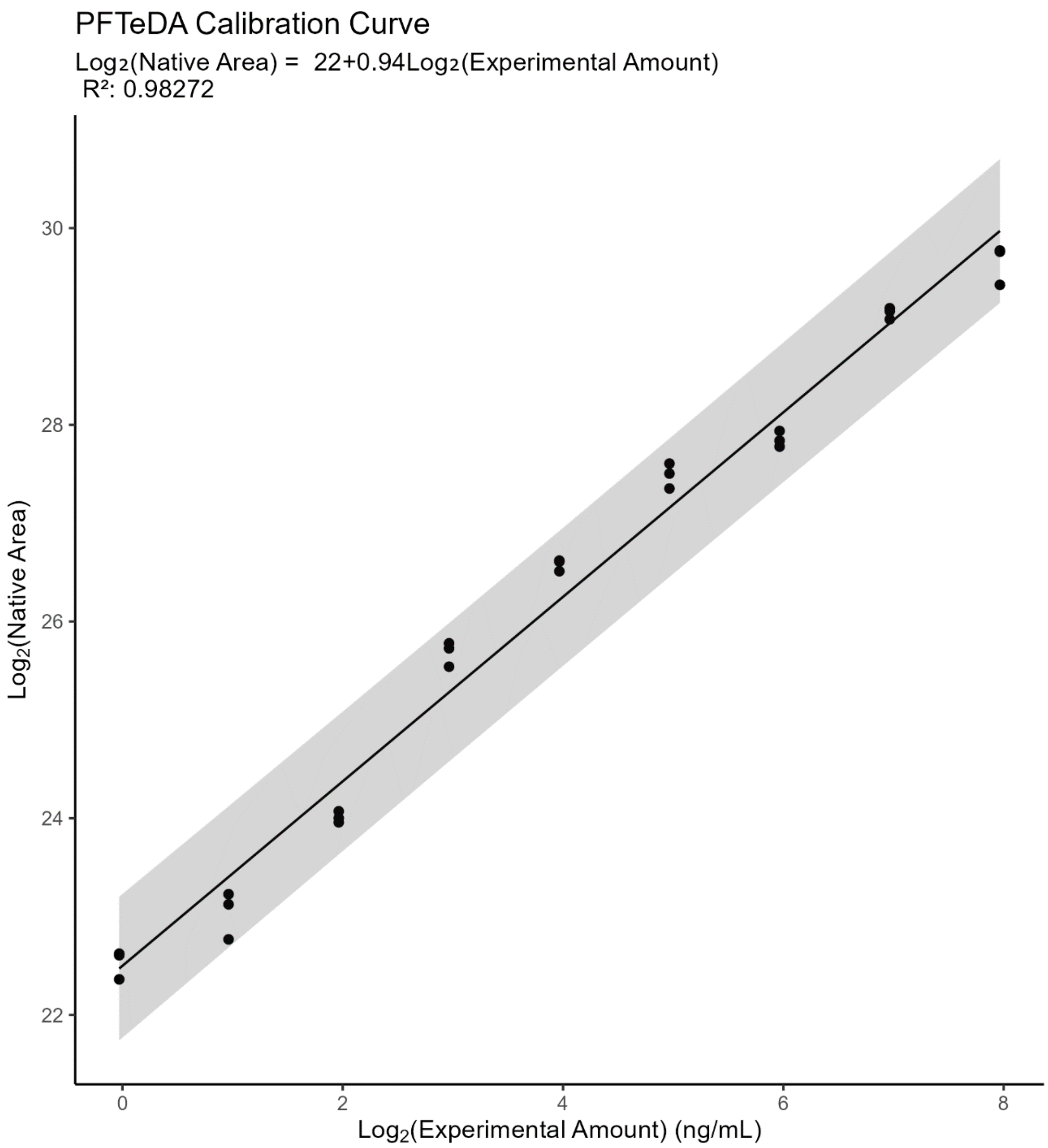
PFTeDA Calibration Curve
Log₂(Native Area) = 22+0.94Log₂(Experimental Amount)
R²: 0.98272
Log₂(Native Area)
30
28
26
24
22
0
2
4
6
8
Log₂(Experimental Amount) (ng/mL)

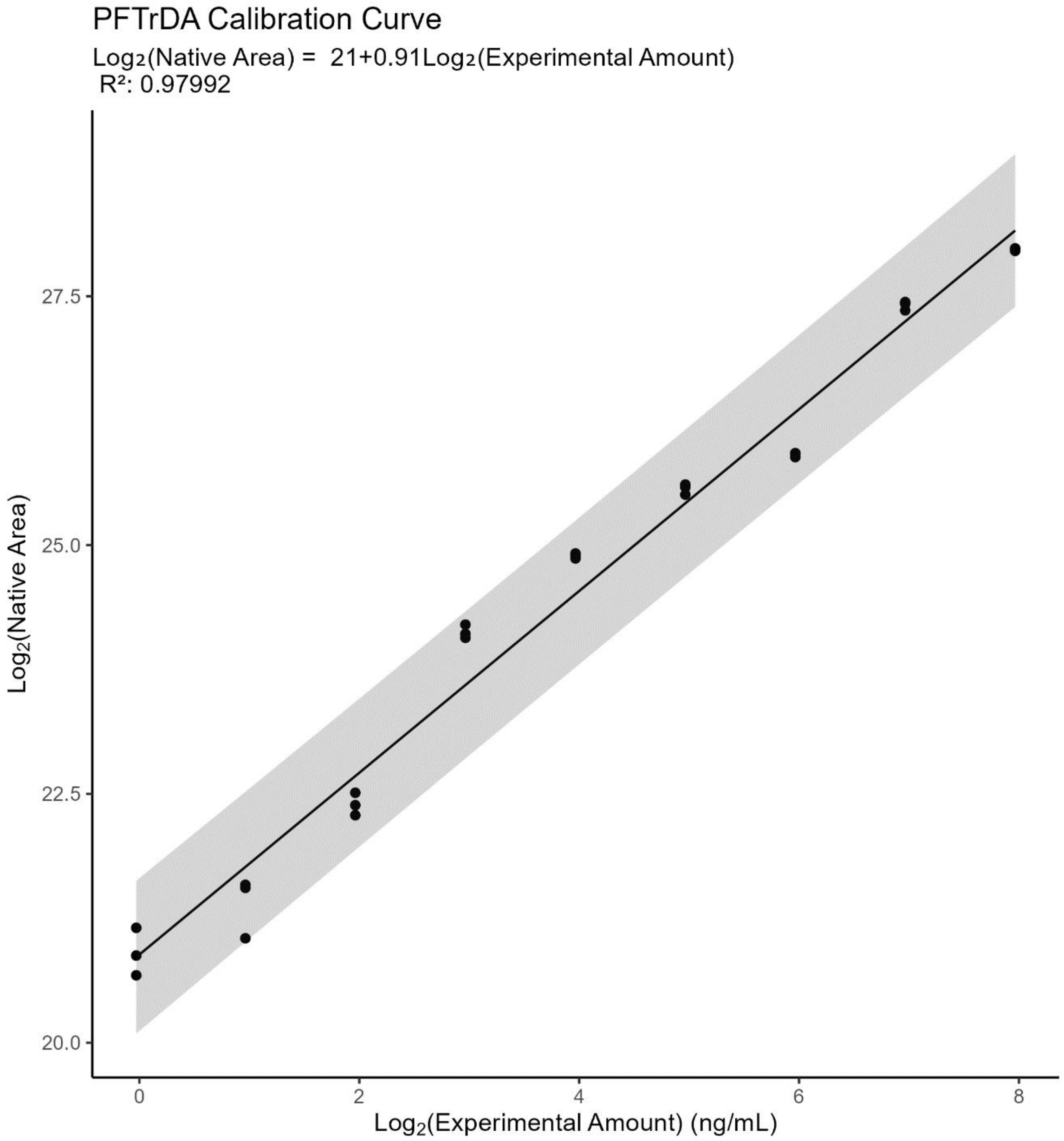

PFTrDA Calibration Curve
Log₂(Native Area) = 21+0.91Log₂(Experimental Amount)
R²: 0.97992
27.5
25.0
22.5
20.0
Log2(Native Area)
0
2
4
6
8
Log2(Experimental Amount) (ng/mL)

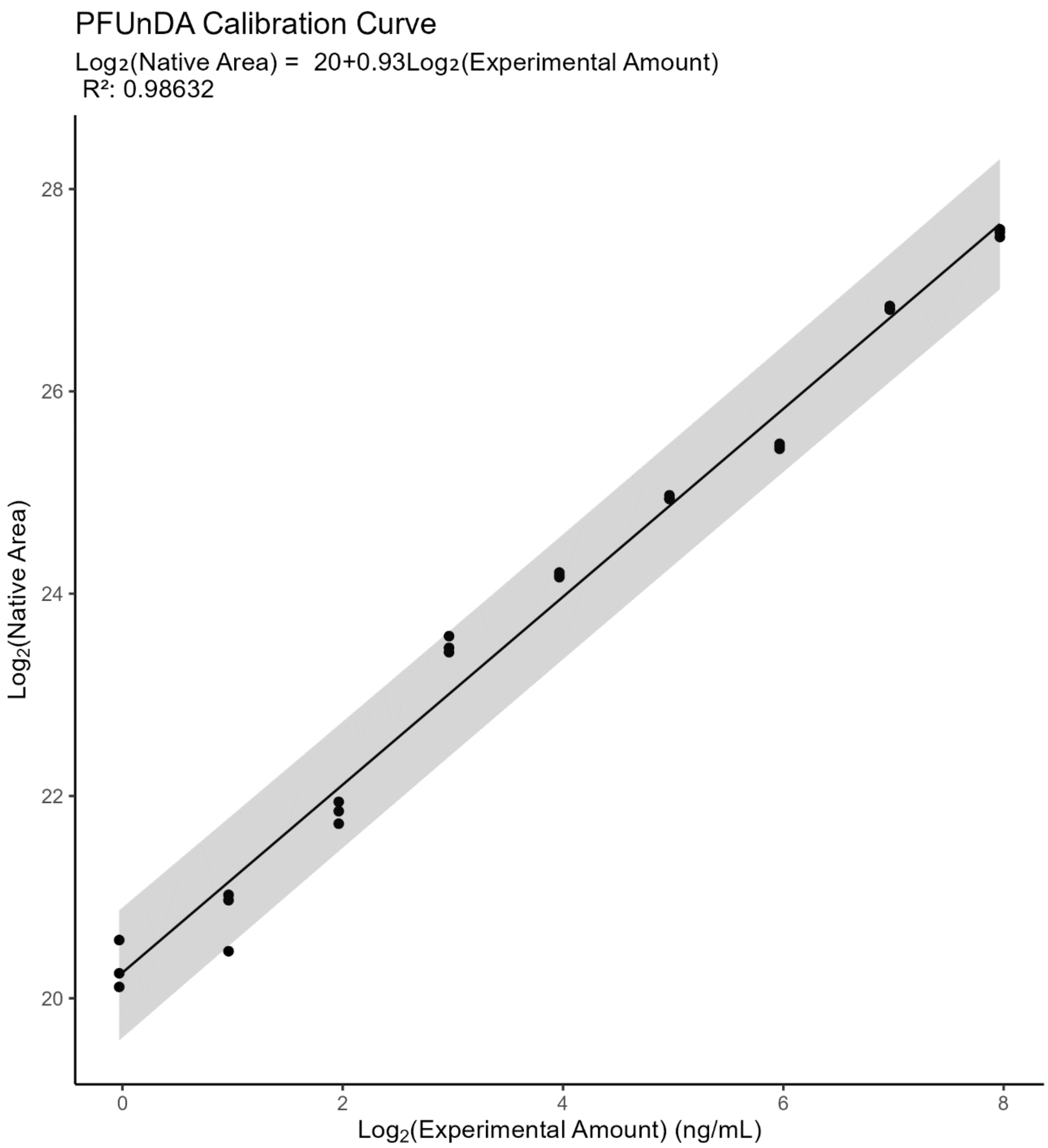
PFUnDA Calibration Curve
Log₂(Native Area) = 20+0.93Log₂(Experimental Amount)
R²: 0.98632
Log2(Native Area)
28
26
24
22
20
0
2
4
6
8
Log2(Experimental Amount) (ng/mL)

Approach 3 Calibration Curves
(n=26)

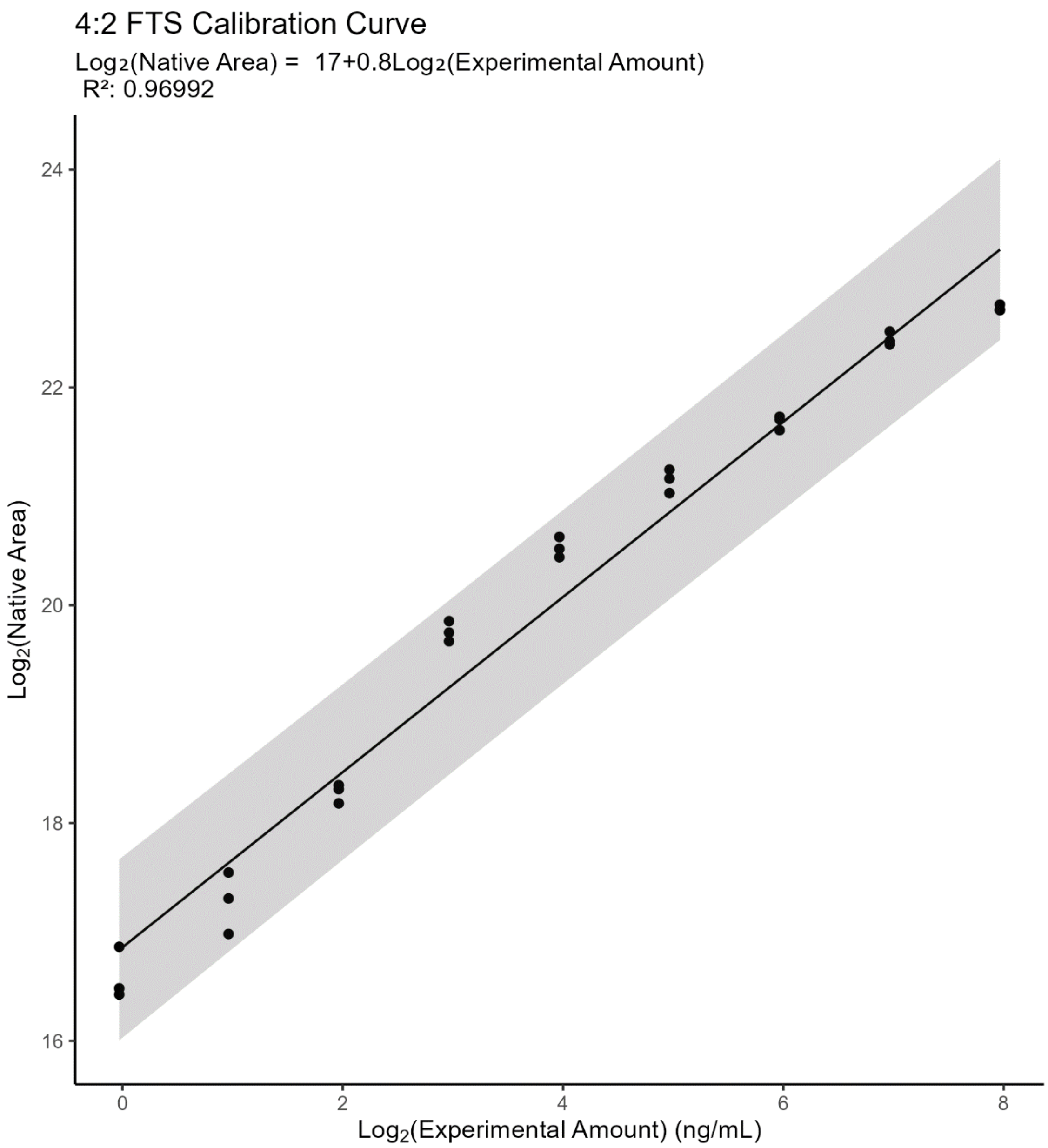
4:2 FTS Calibration Curve
Log₂(Native Area) = 17+0.8Log₂(Experimental Amount)
R²: 0.96992
Log₂(Native Area)
24
22
20
18
16
0
2
4
6
8
Log₂(Experimental Amount) (ng/mL)

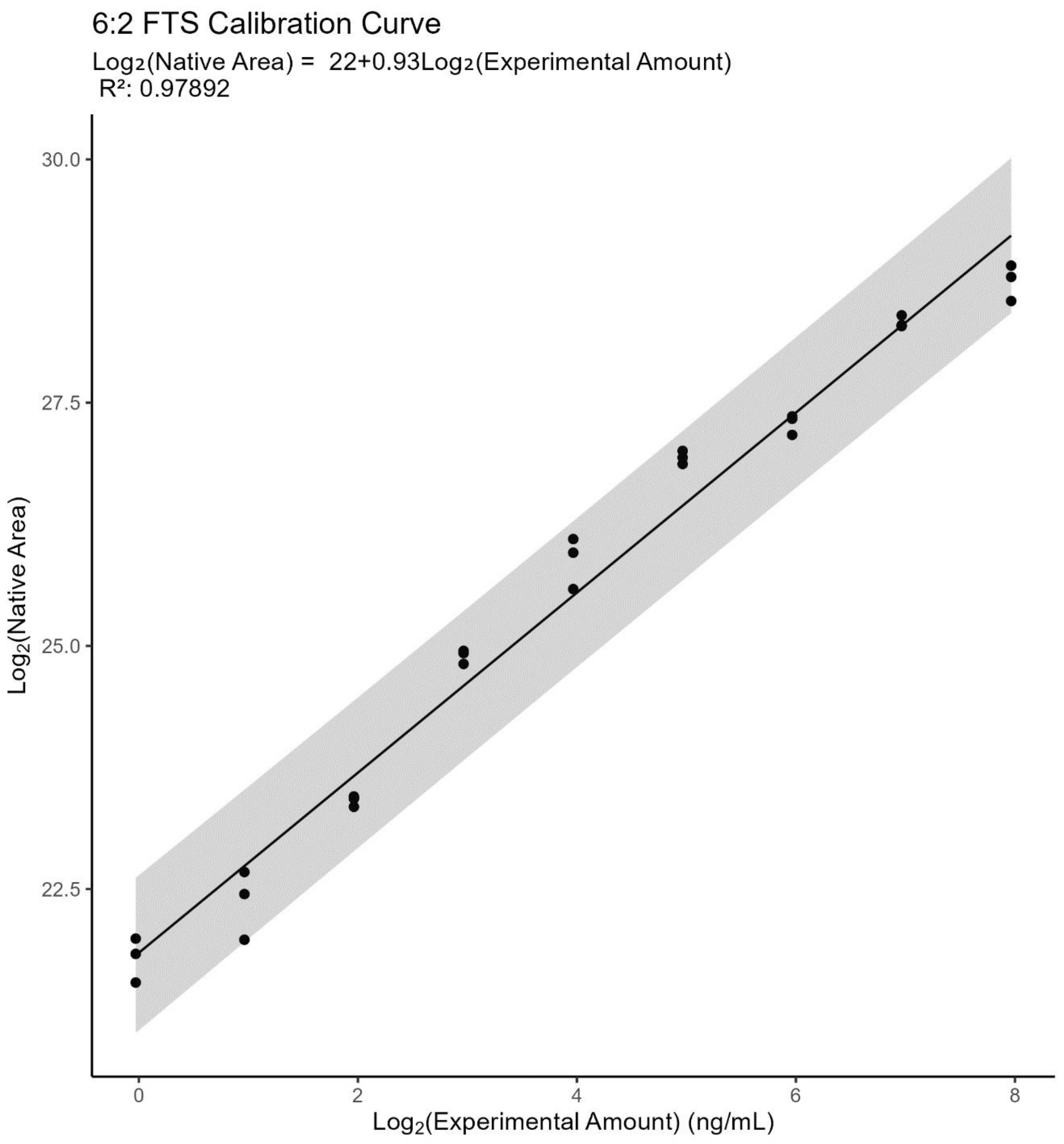
6:2 FTS Calibration Curve
Log₂(Native Area) = 22+0.93Log₂(Experimental Amount)
R²: 0.97892
30.0
27.5
25.0
22.5
Log2(Native Area)
0
2
4
6
8
Log2(Experimental Amount) (ng/mL)

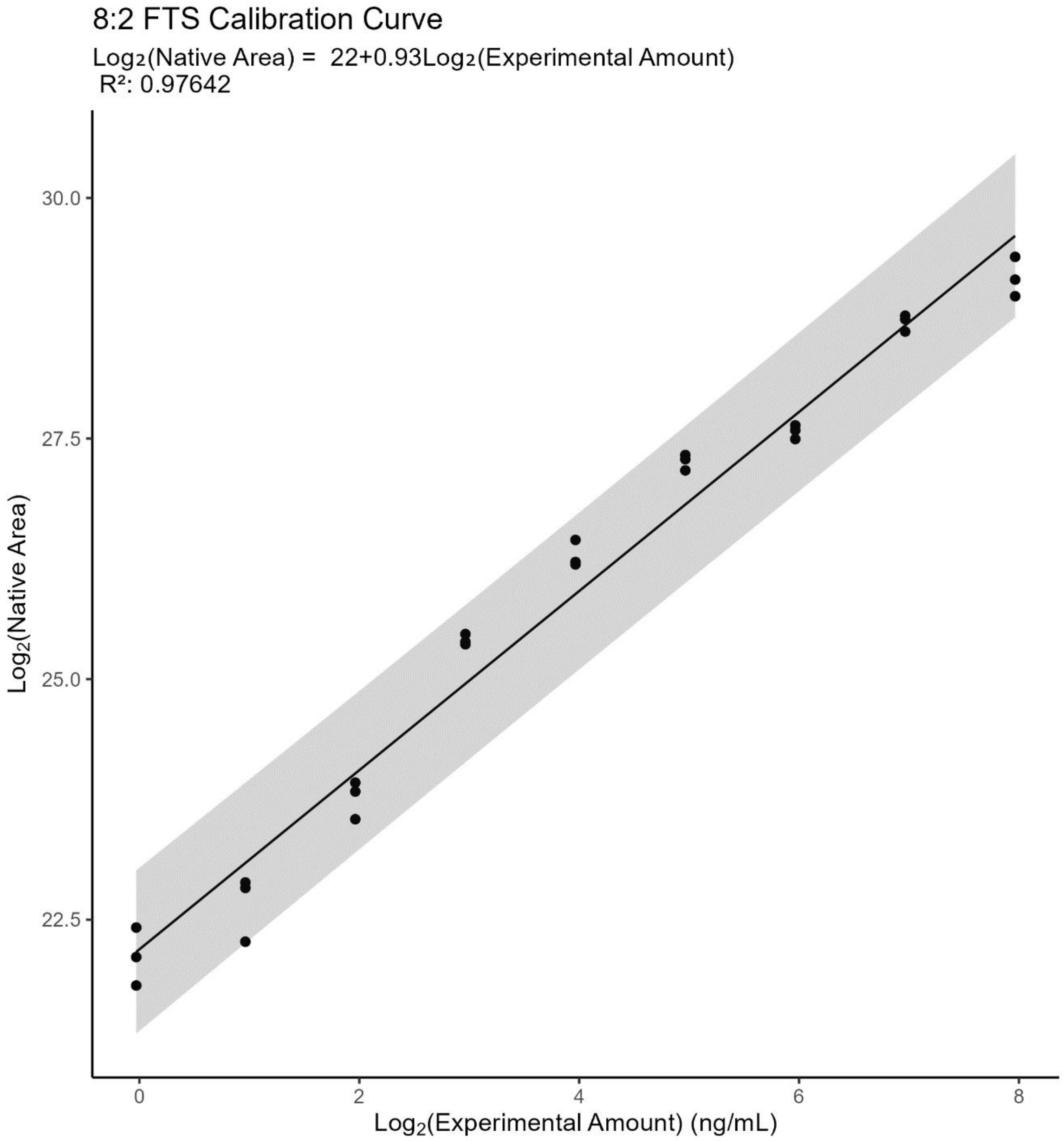
8:2 FTS Calibration Curve
Log₂(Native Area) = 22+0.93Log₂(Experimental Amount)
R²: 0.97642
30.0
27.5
25.0
22.5
Log2(Native Area)
0
2
4
6
8
Log2(Experimental Amount) (ng/mL)

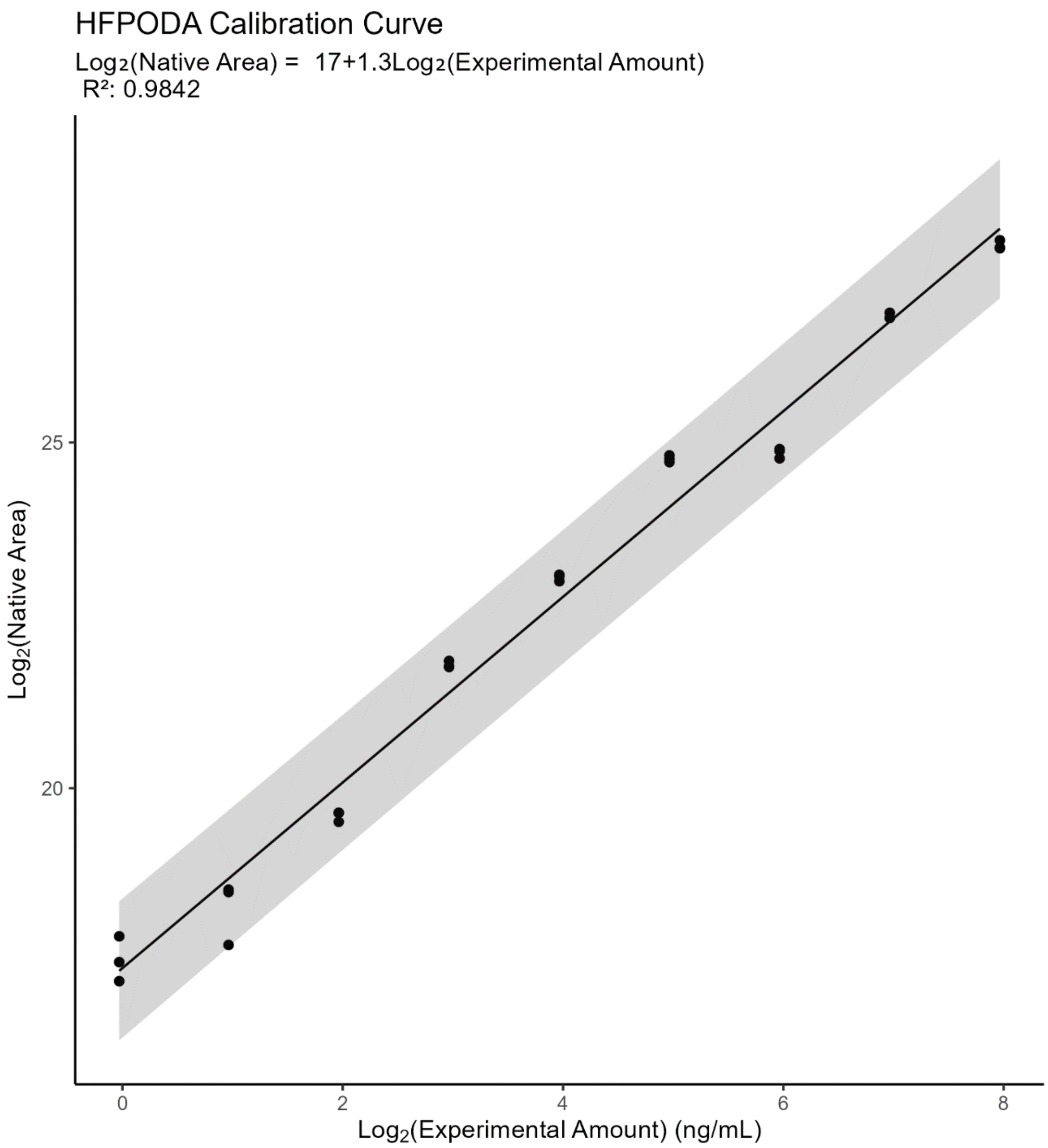
HFPODA Calibration Curve
Log₂(Native Area) = 17+1.3Log₂(Experimental Amount)
R²: 0.9842
Log2(Native Area)
25
20
0
2
4
6
8
Log2(Experimental Amount) (ng/mL)

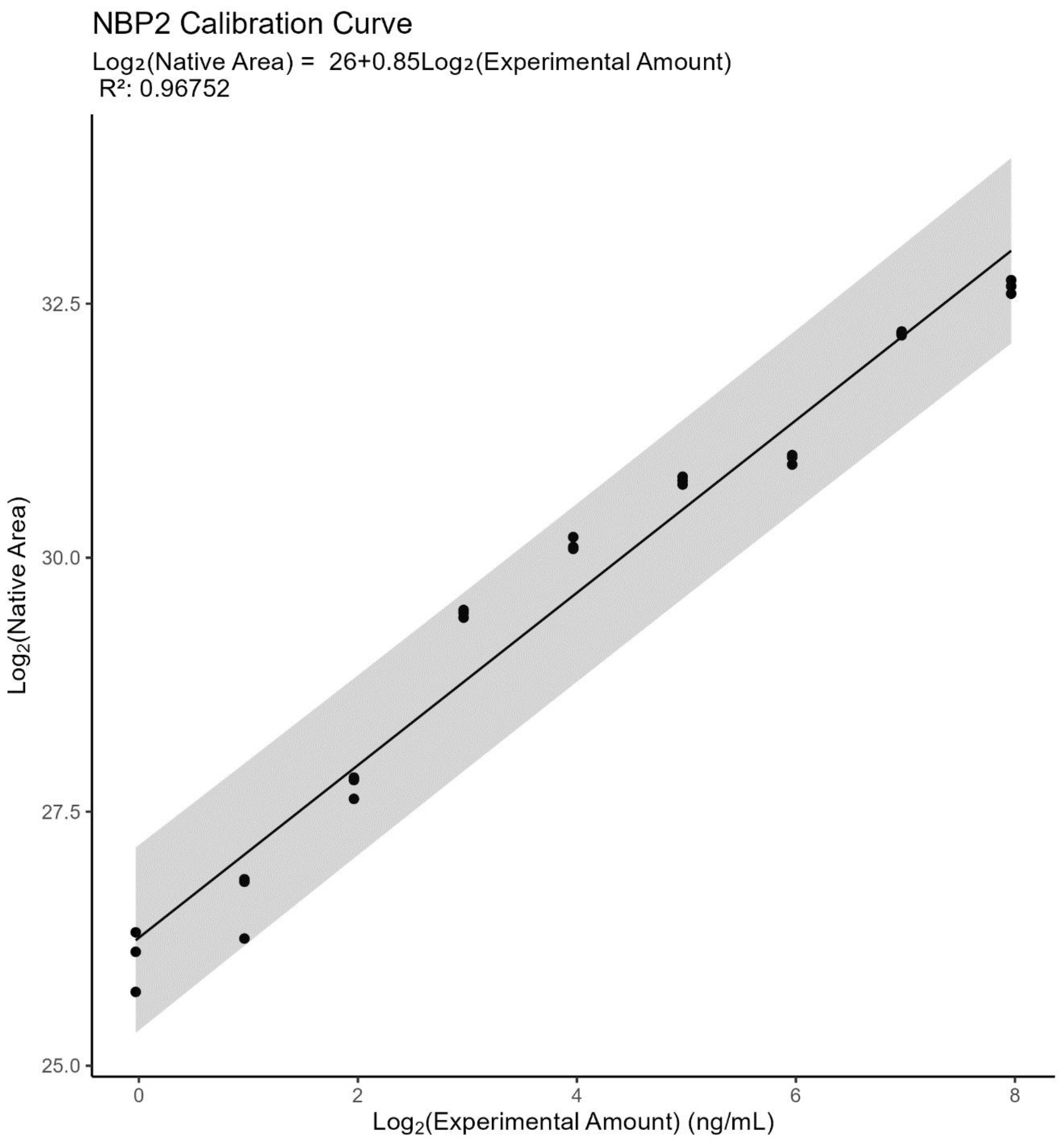

NBP2 Calibration Curve
Log₂(Native Area) = 26+0.85Log₂(Experimental Amount)
R²: 0.96752
Log2(Native Area)
32.5
30.0
27.5
25.0
0
2
4
6
8
Log2(Experimental Amount) (ng/mL)

N-EtFOSAA Calibration Curve

$Log_2(\text{Native Area}) = 22+0.8Log_2(\text{Experimental Amount})$

R^2: 0.96622

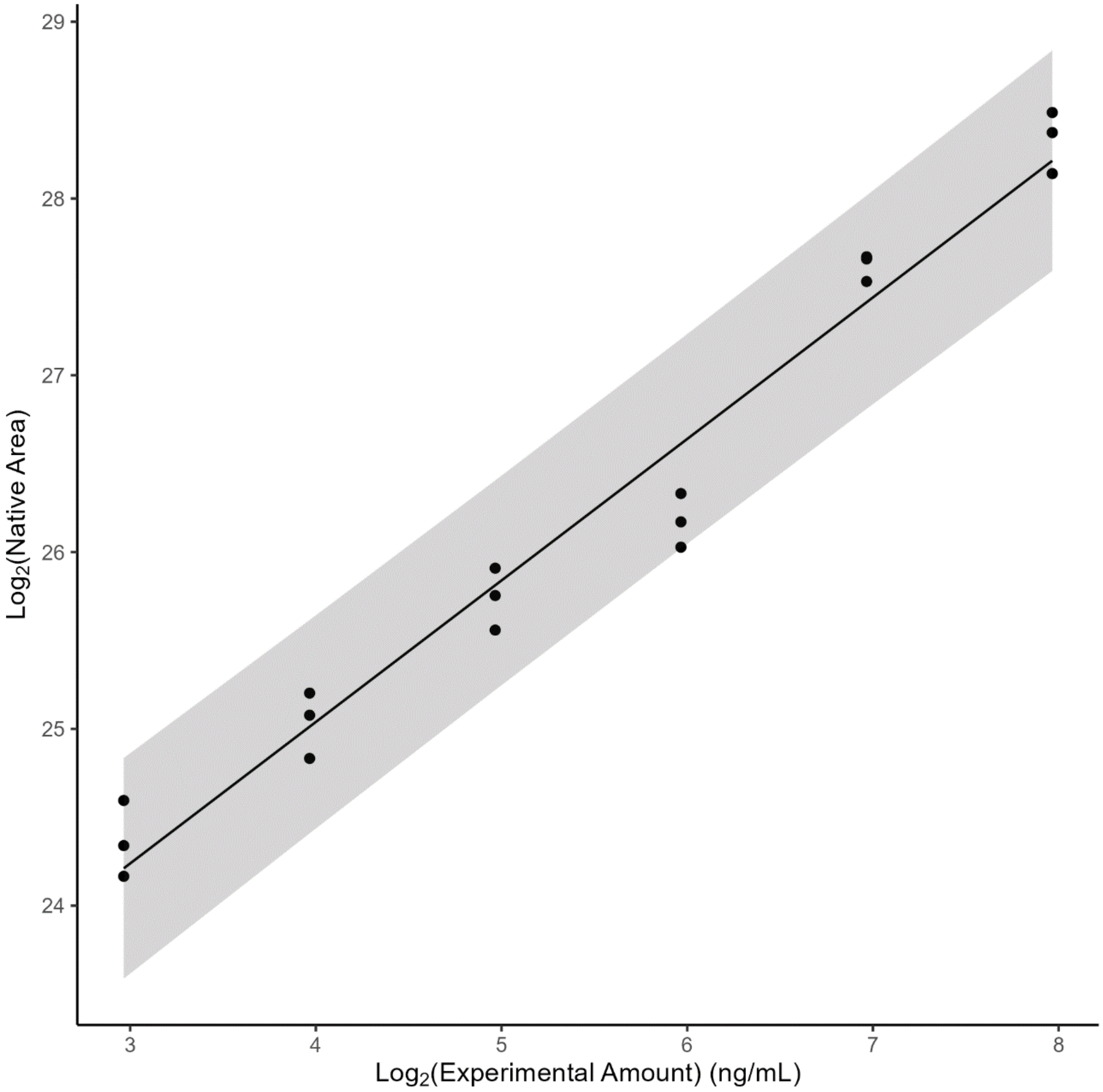

N-MeFOSAA Calibration Curve

$\log_2$(Native Area) = 21+0.85$\log_2$(Experimental Amount)

R^2: 0.9822

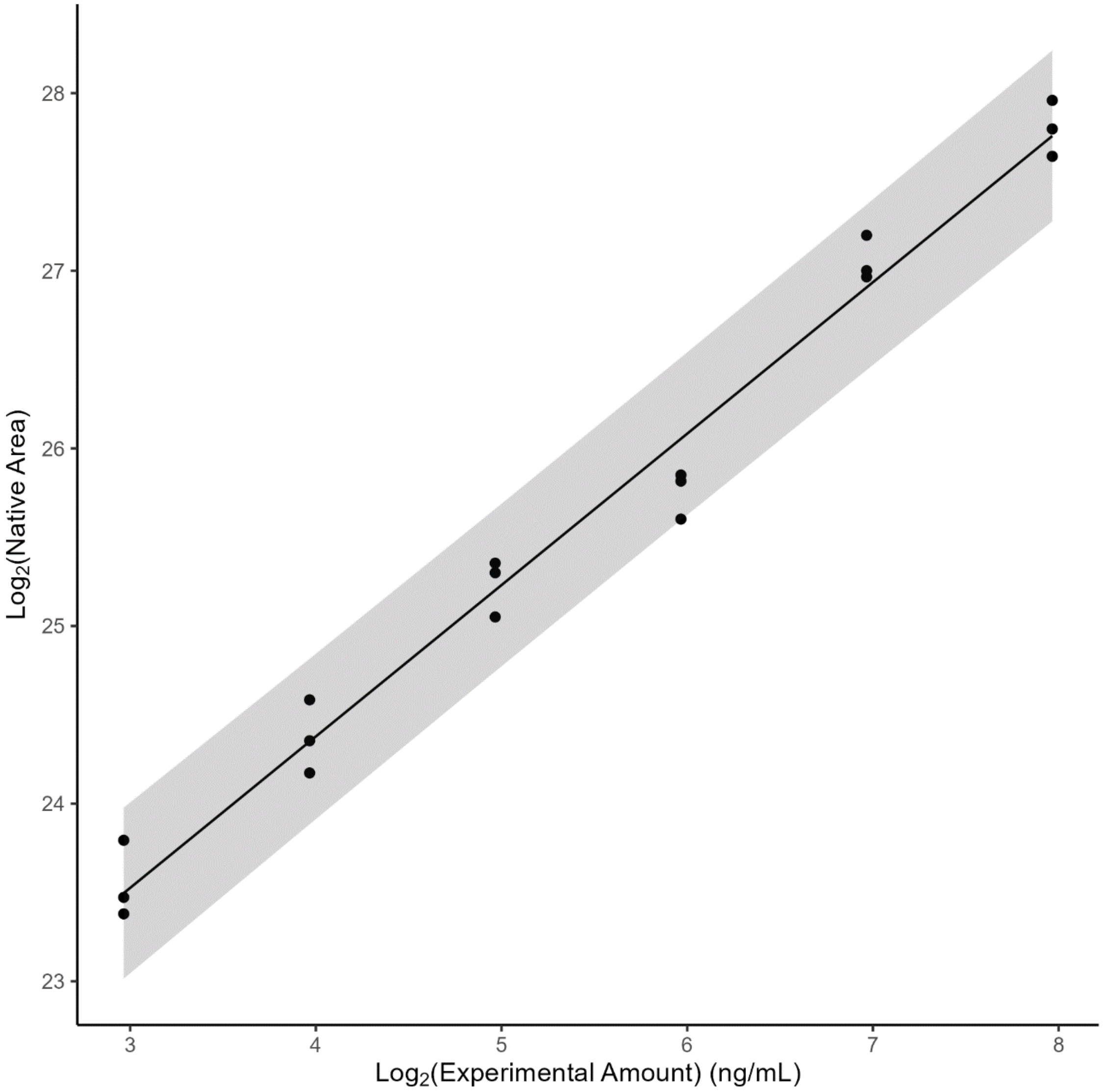

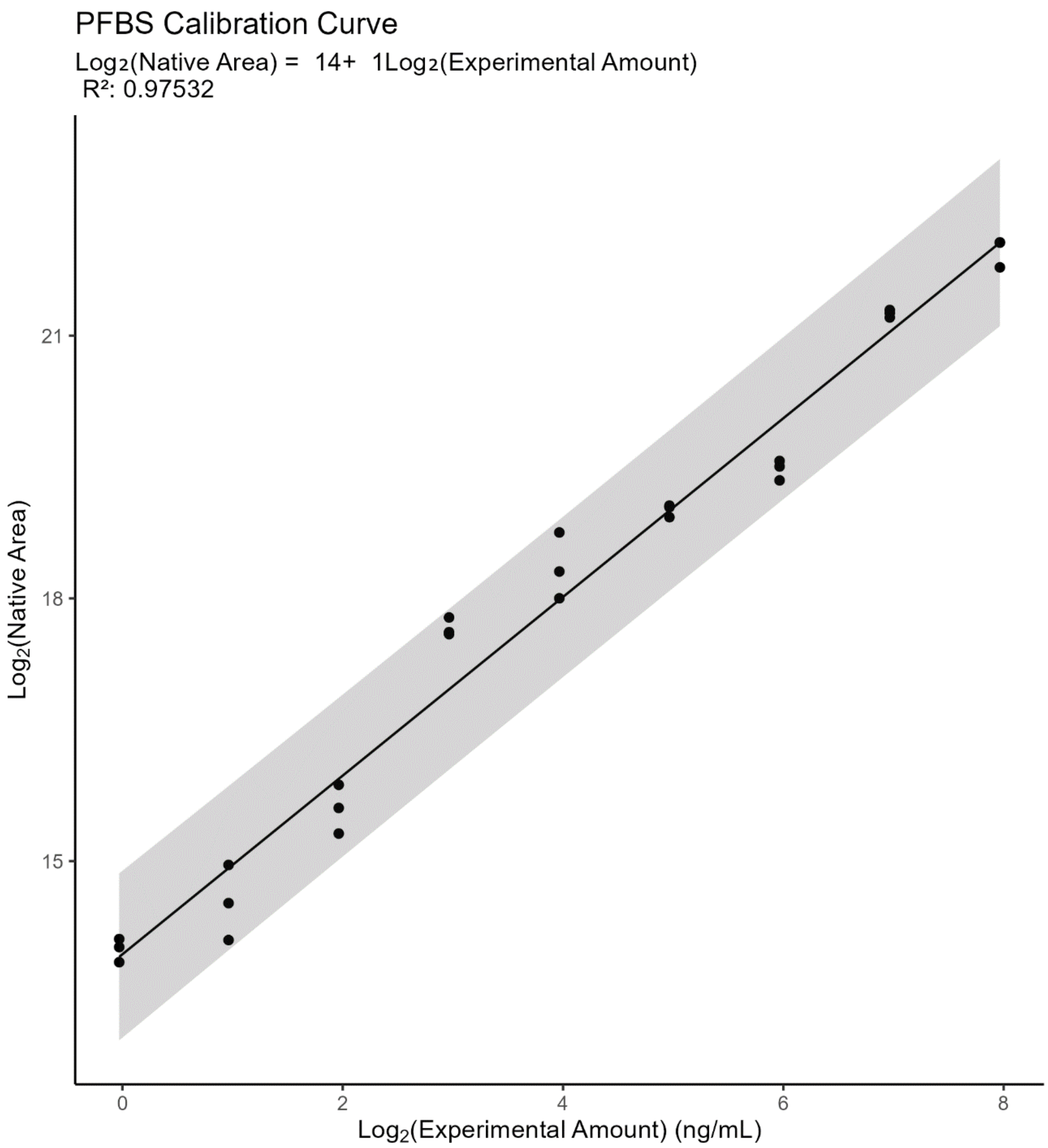

PFBS Calibration Curve
Log₂(Native Area) = 14+ 1Log₂(Experimental Amount)
R²: 0.97532
Log₂(Native Area)
21
18
15
0
2
4
6
8
Log₂(Experimental Amount) (ng/mL)

PFDA Calibration Curve

Log_2(Native Area) = 21+0.9Log_2(Experimental Amount)
R^2: 0.97912

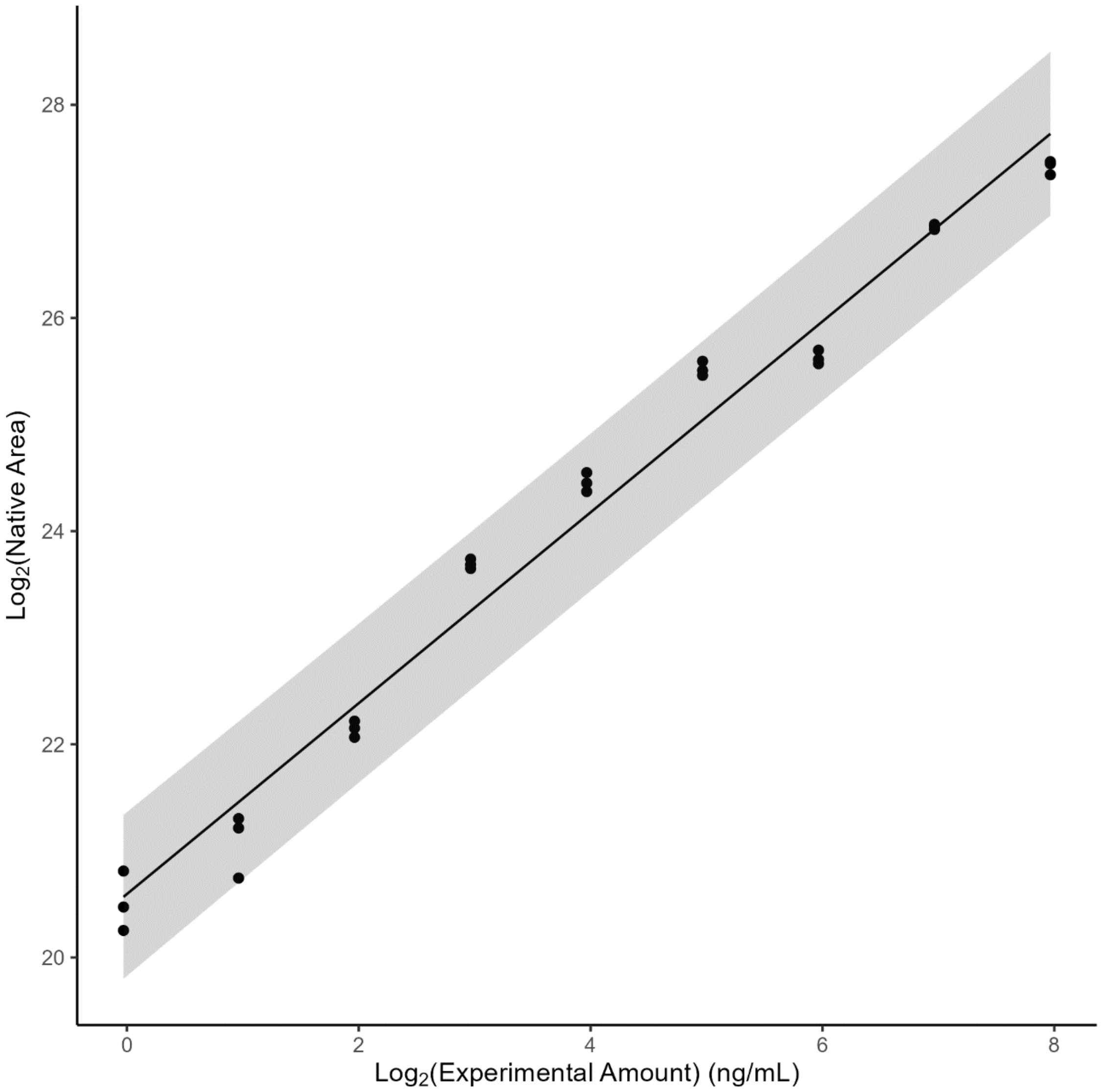

PFDoDA Calibration Curve

Log_2(Native Area) = 21+ 1Log_2(Experimental Amount)
R^2: 0.92392

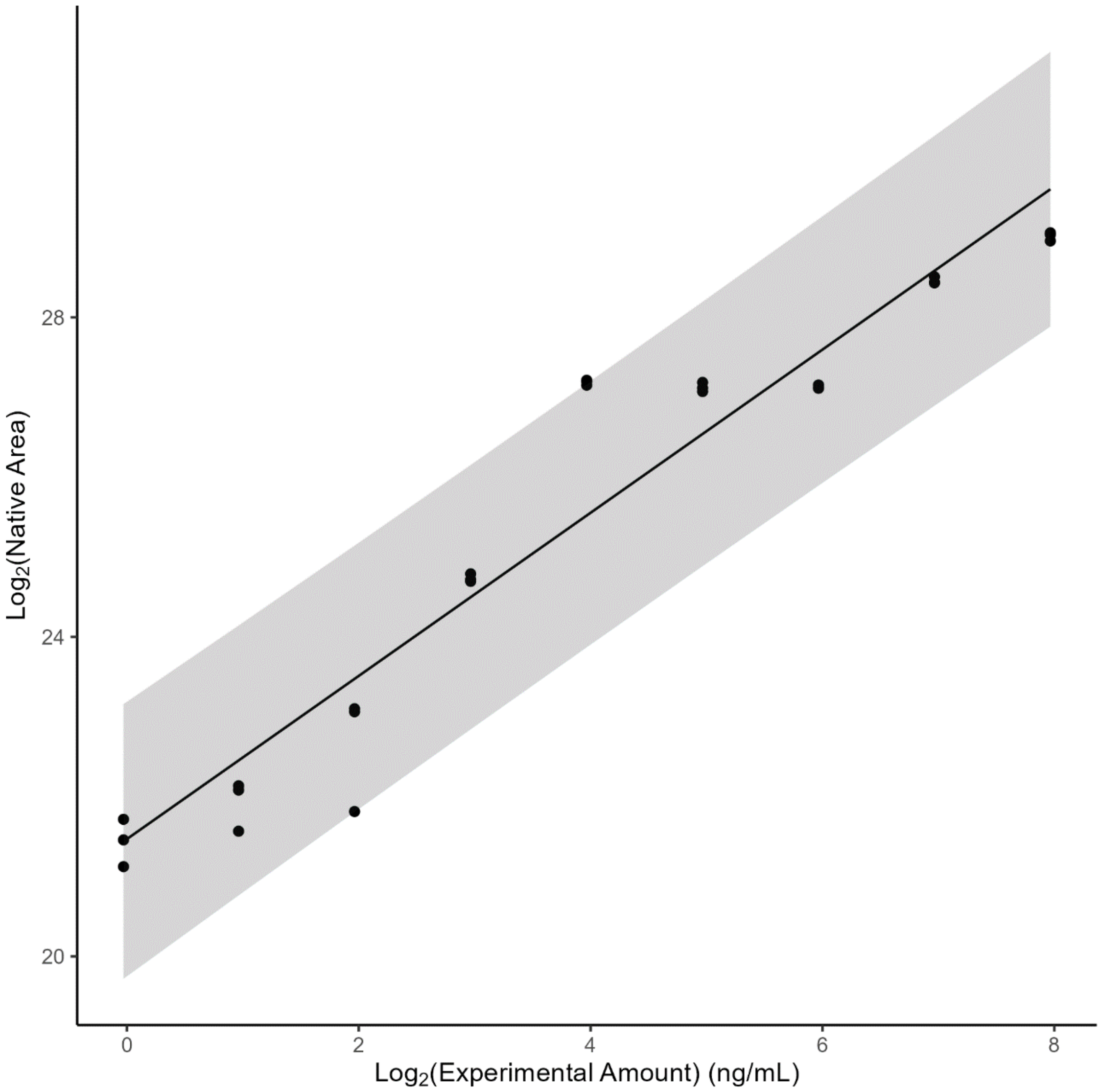

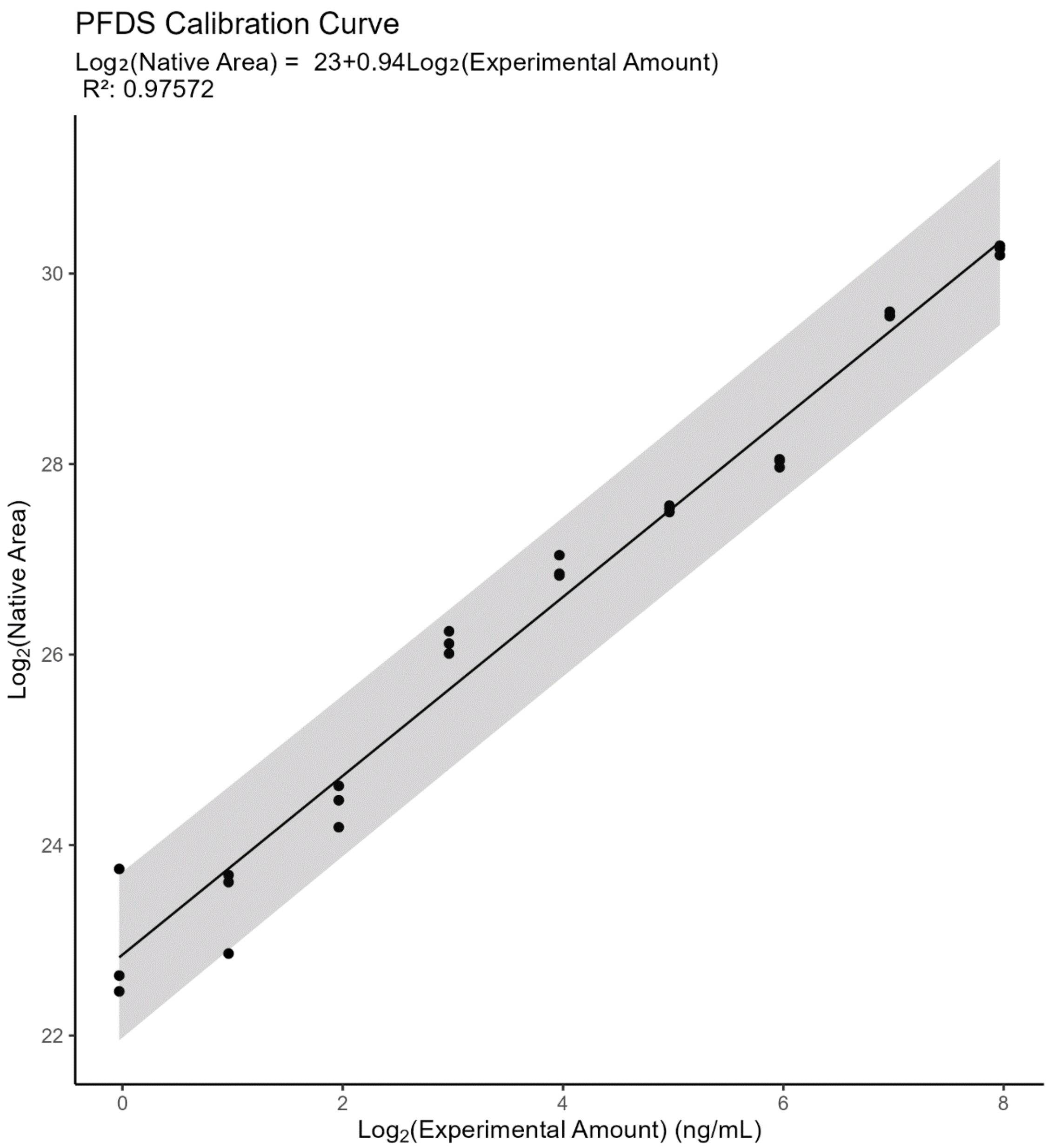

PFDS Calibration Curve
Log_2(Native Area) = 23+0.94Log_2(Experimental Amount)
R^2: 0.97572
Log_2(Native Area)
22
24
26
28
30
0
2
4
6
8
Log_2(Experimental Amount) (ng/mL)

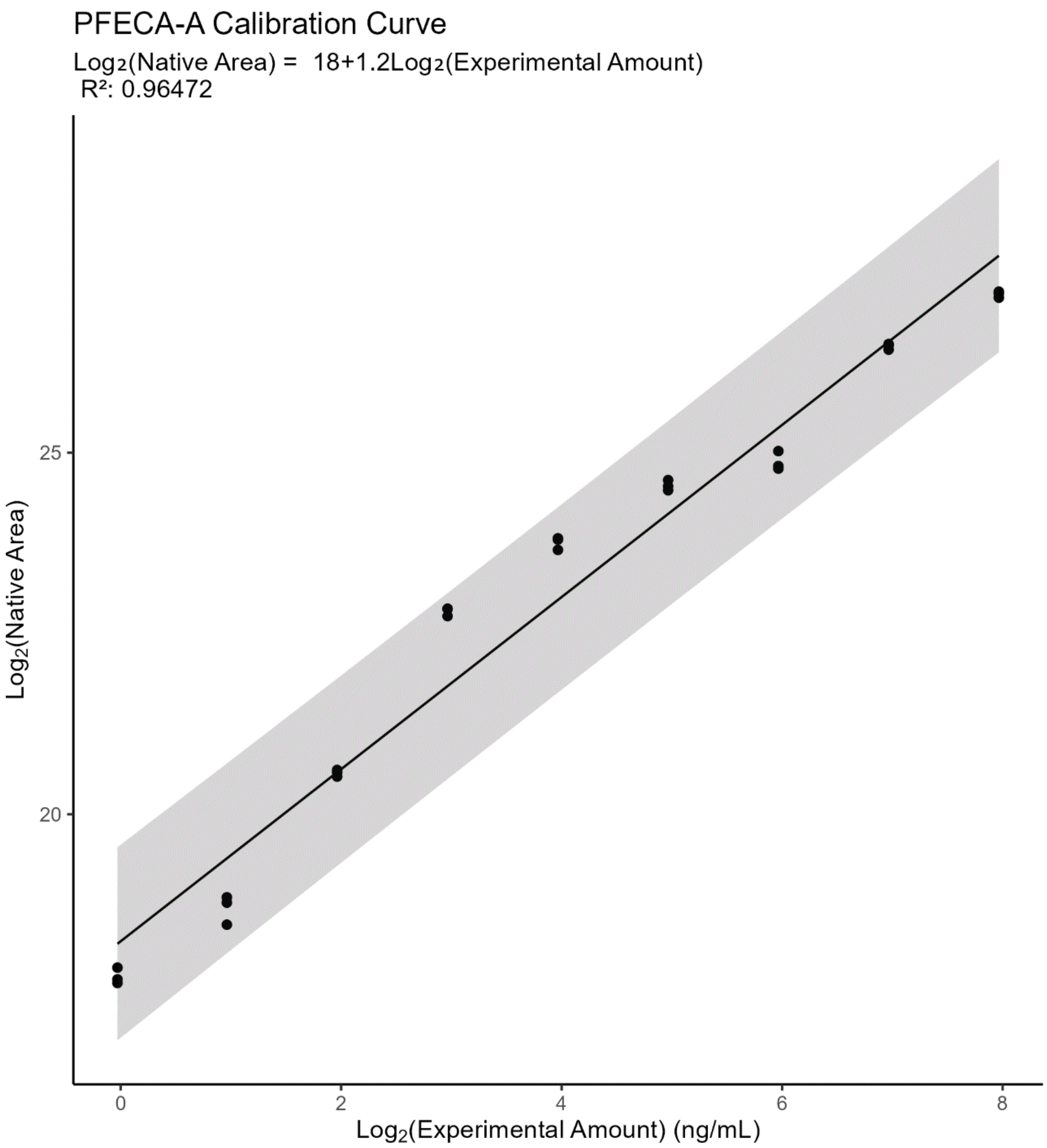
PFECA-A Calibration Curve
Log₂(Native Area) = 18+1.2Log₂(Experimental Amount)
R²: 0.96472
25
20
Log₂(Native Area)
0
2
4
6
8
Log₂(Experimental Amount) (ng/mL)

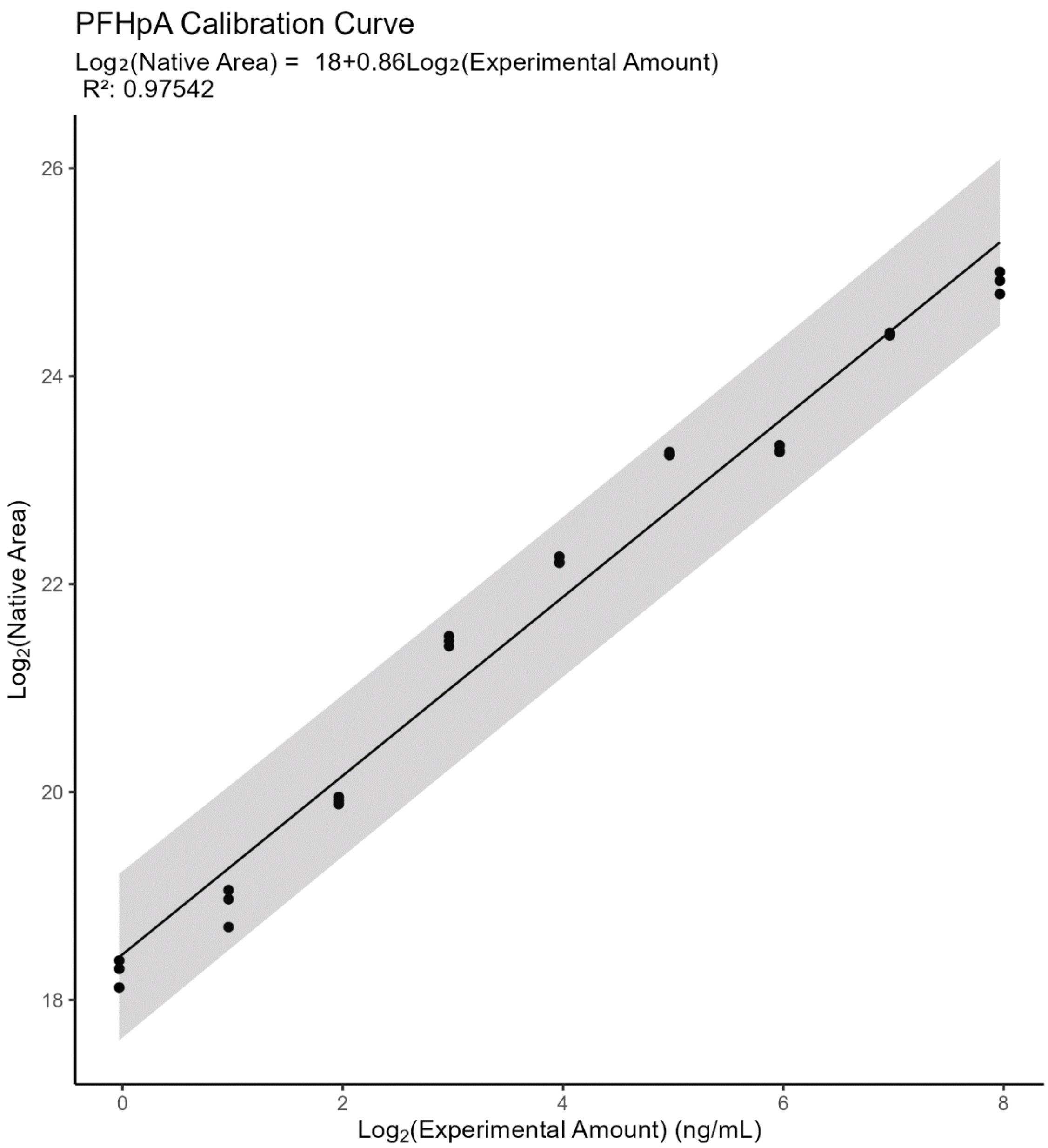

PFHpA Calibration Curve
Log₂(Native Area) = 18+0.86Log₂(Experimental Amount)
R²: 0.97542
26
24
22
20
18
Log2(Native Area)
0
2
4
6
8
Log2(Experimental Amount) (ng/mL)

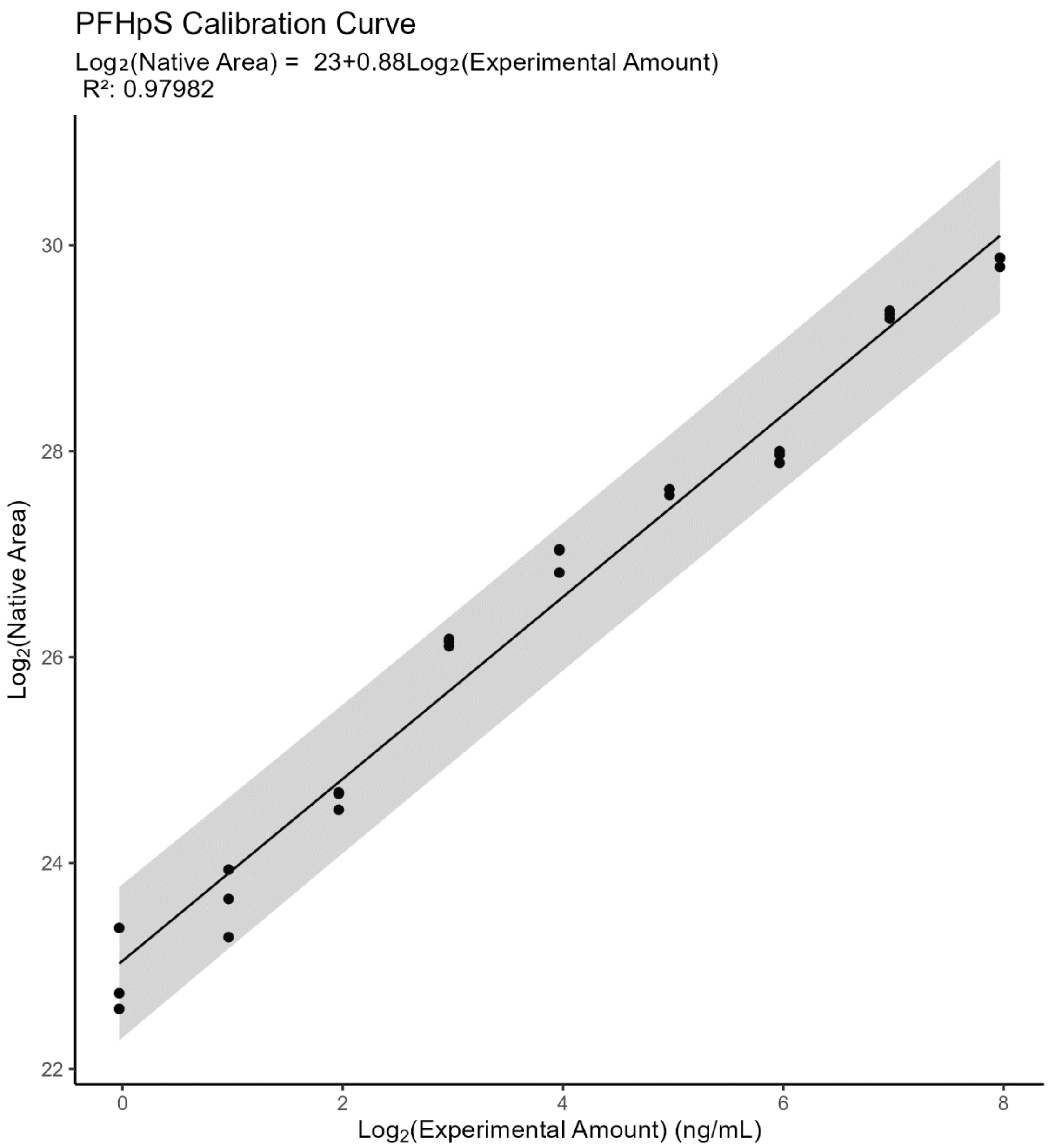

PFHpS Calibration Curve
Log₂(Native Area) = 23+0.88Log₂(Experimental Amount)
R²: 0.97982
Log2(Native Area)
30
28
26
24
22
0
2
4
6
8
Log2(Experimental Amount) (ng/mL)

PFHxA Calibration Curve

$Log_2(\text{Native Area}) = 13+1.4Log_2(\text{Experimental Amount})$

R^2: 0.98952

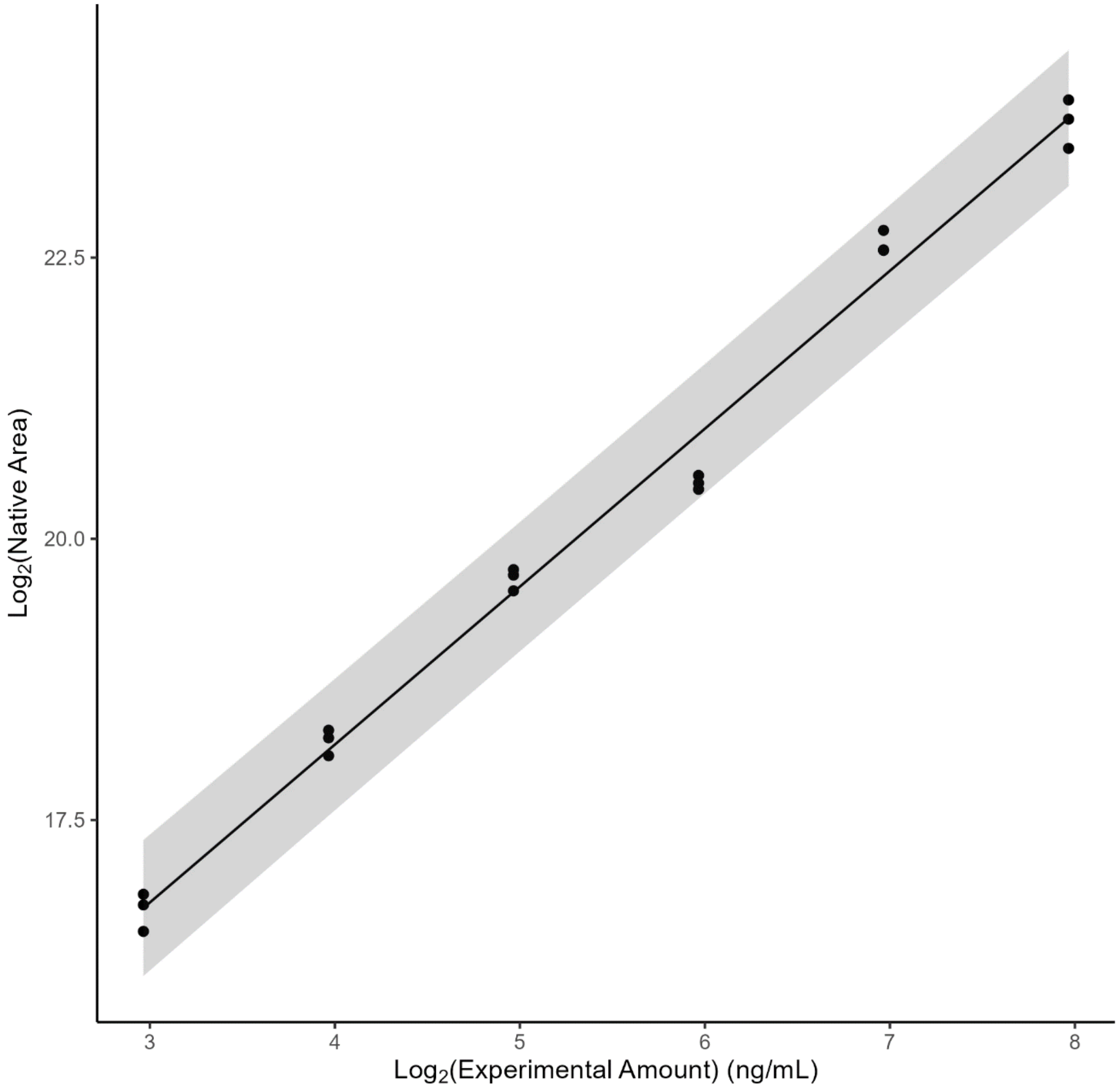

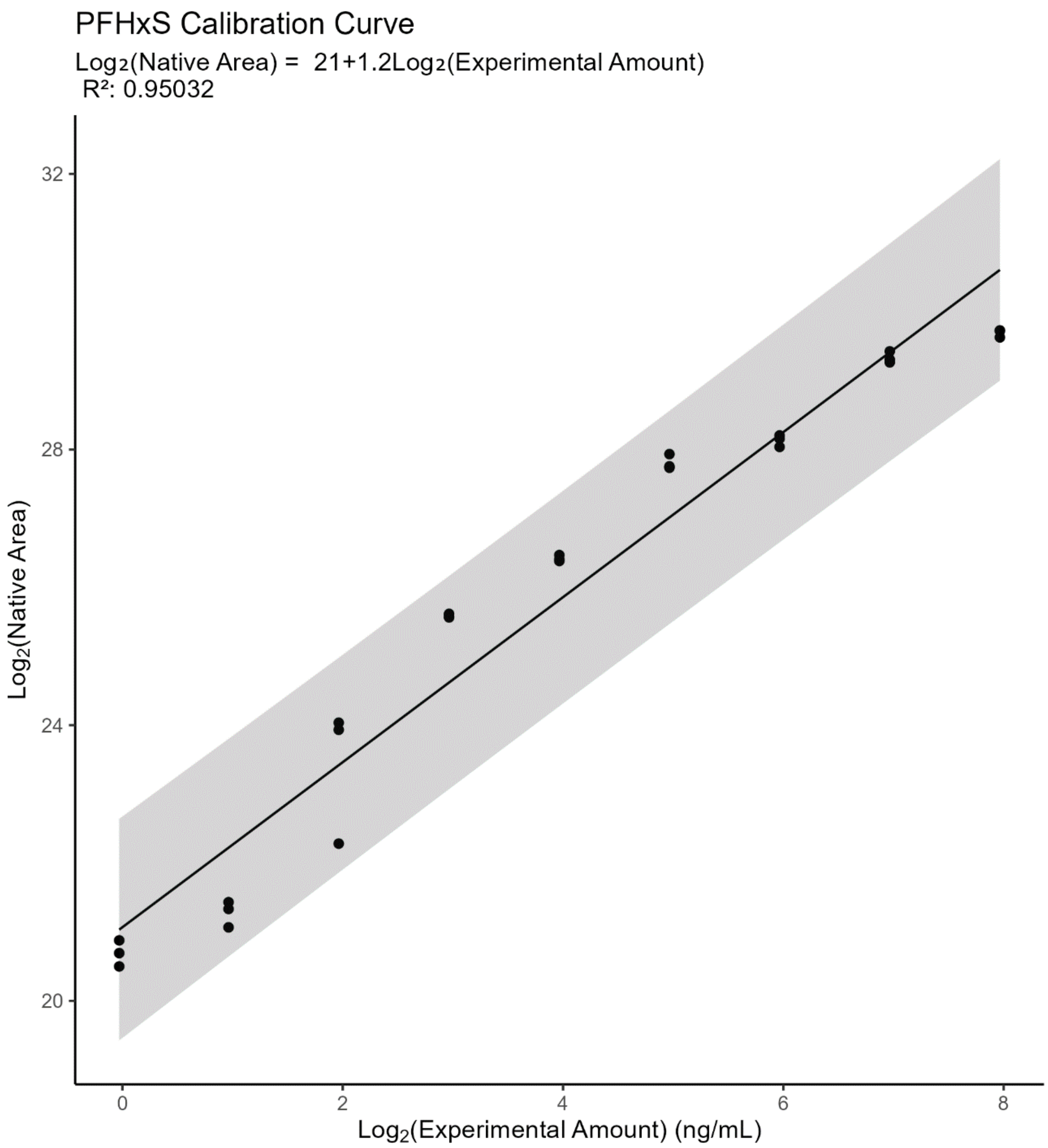

PFHxS Calibration Curve
Log_2(Native Area) = 21+1.2Log_2(Experimental Amount)
R^2: 0.95032
32
28
24
20
Log_2(Native Area)
0
2
4
6
8
Log_2(Experimental Amount) (ng/mL)

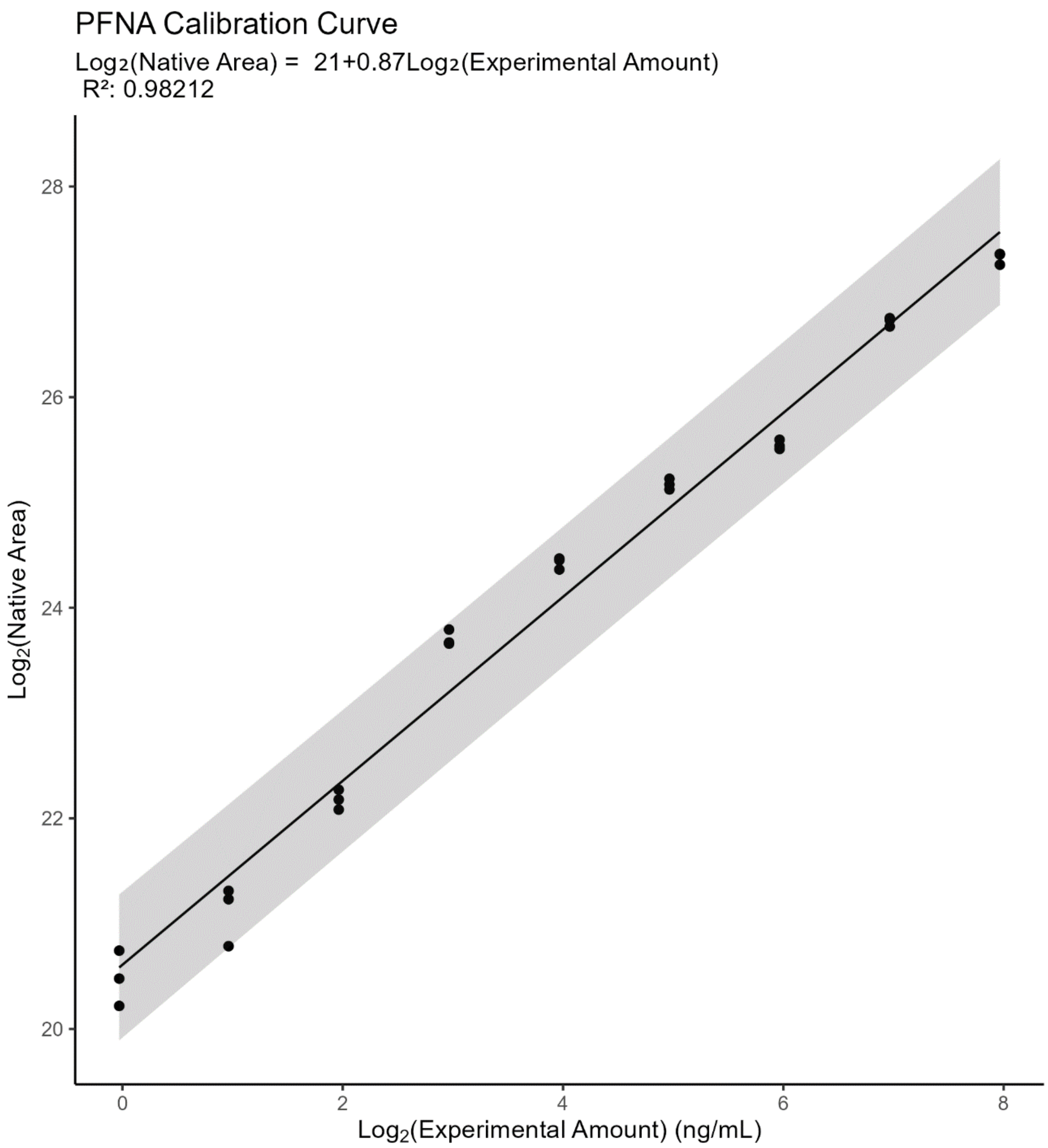
PFNA Calibration Curve
Log₂(Native Area) = 21+0.87Log₂(Experimental Amount)
R²: 0.98212
Log2(Native Area)
Log2(Experimental Amount) (ng/mL)
28
26
24
22
20
0
2
4
6
8

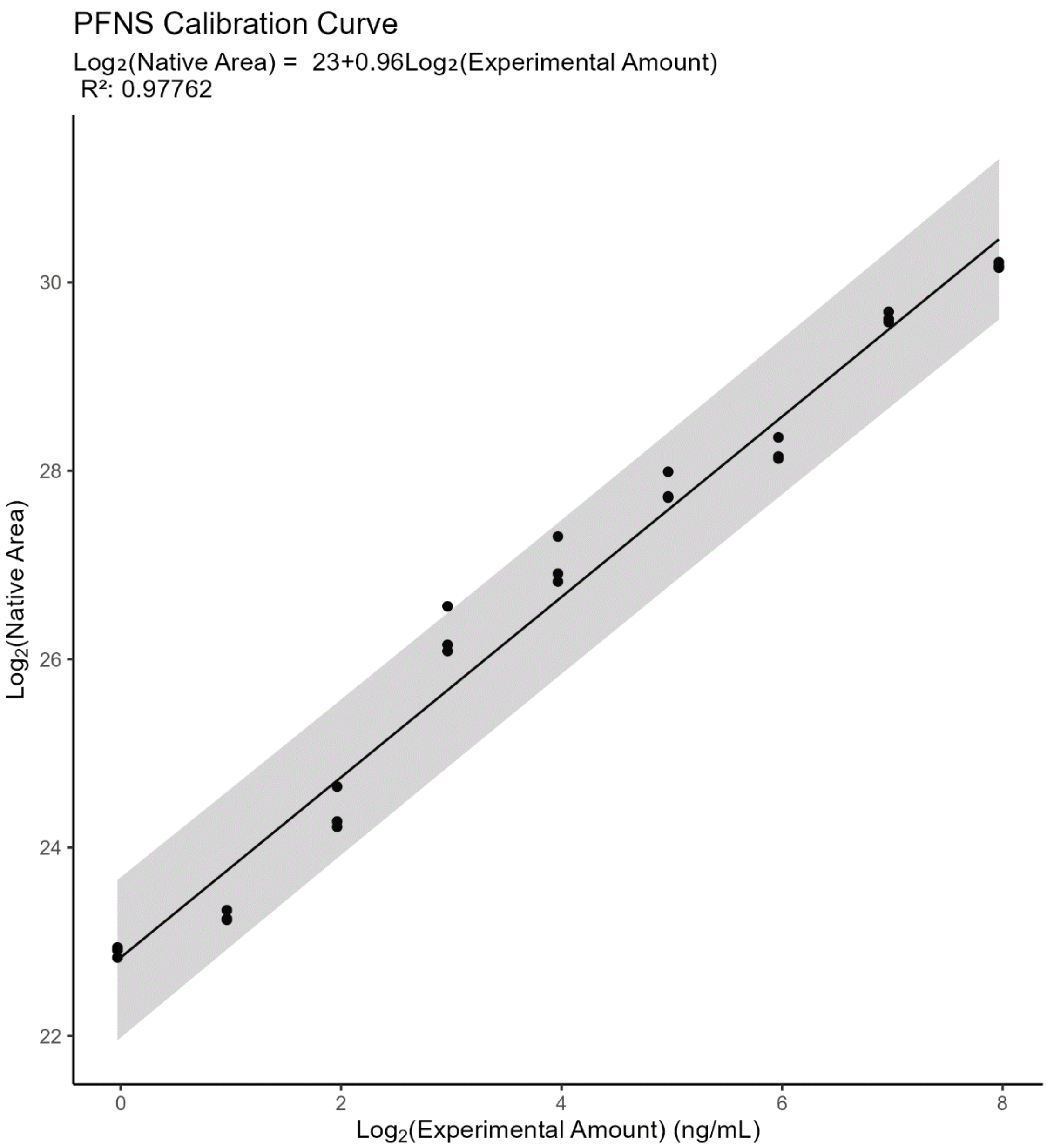
PFNS Calibration Curve
Log₂(Native Area) = 23+0.96Log₂(Experimental Amount)
R²: 0.97762
Log2(Native Area)
30
28
26
24
22
0
2
4
6
8
Log2(Experimental Amount) (ng/mL)

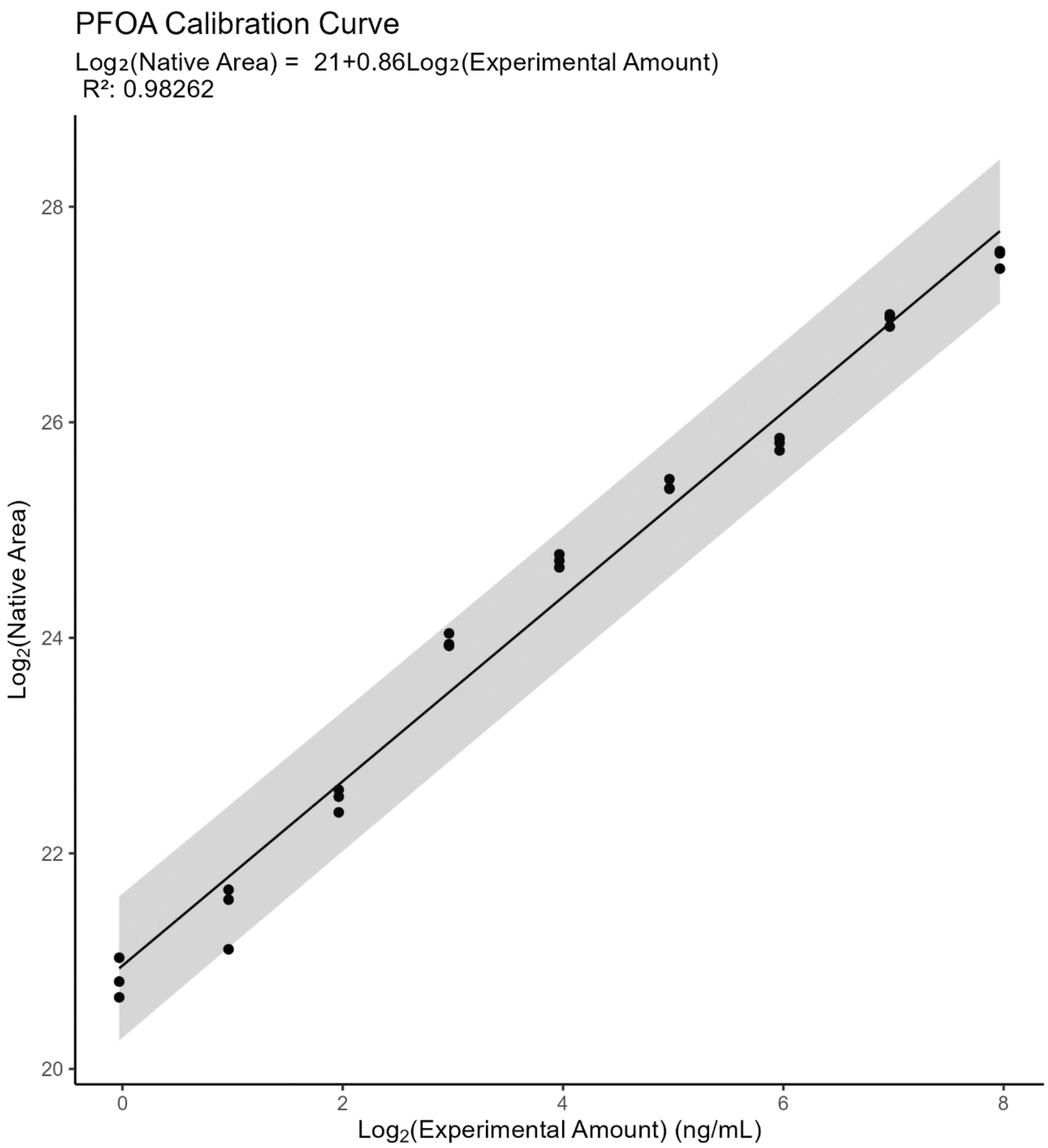

PFOA Calibration Curve
Log₂(Native Area) = 21+0.86Log₂(Experimental Amount)
R²: 0.98262
28
26
24
22
20
Log2(Native Area)
0
2
4
6
8
Log2(Experimental Amount) (ng/mL)

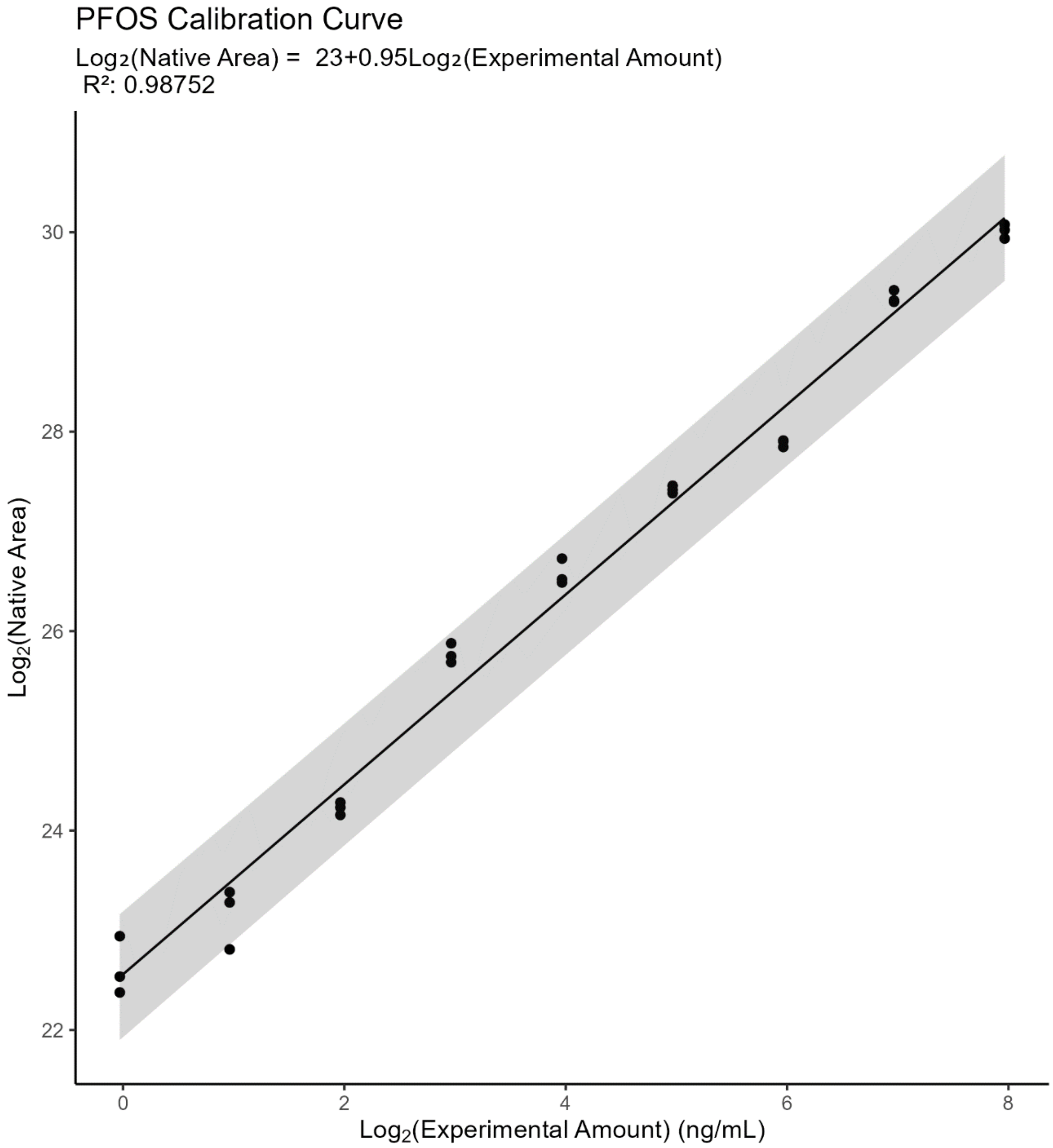
PFOS Calibration Curve
Log₂(Native Area) = 23+0.95Log₂(Experimental Amount)
R²: 0.98752
Log2(Native Area)
Log2(Experimental Amount) (ng/mL)
22
24
26
28
30
0
2
4
6
8

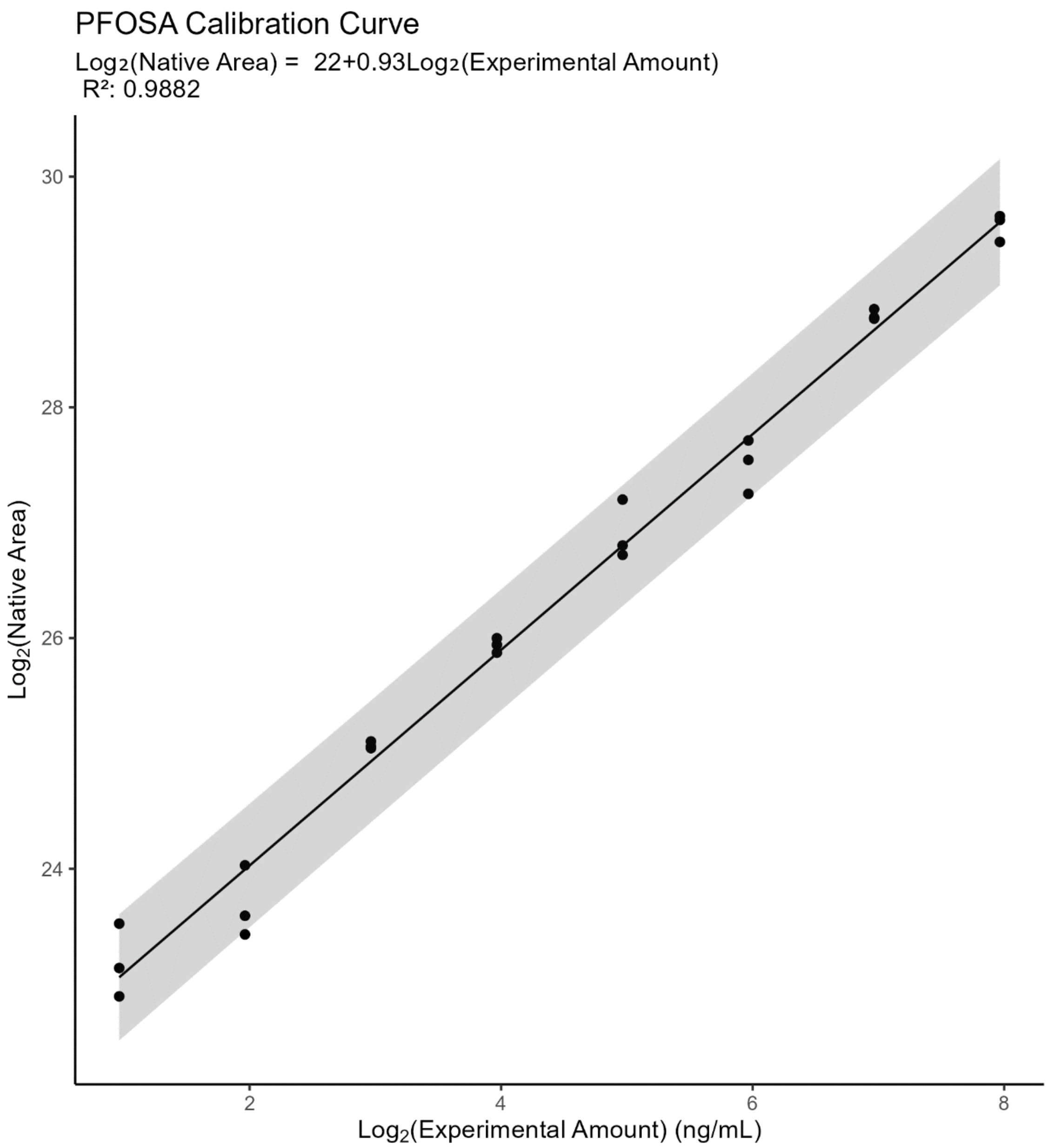
PFOSA Calibration Curve
Log₂(Native Area) = 22+0.93Log₂(Experimental Amount)
R²: 0.9882
30
28
26
24
Log2(Native Area)
2
4
6
8
Log2(Experimental Amount) (ng/mL)

PFPeA Calibration Curve

$\log_2(\text{Native Area}) = 15+0.99\log_2(\text{Experimental Amount})$

R^2: 0.97552

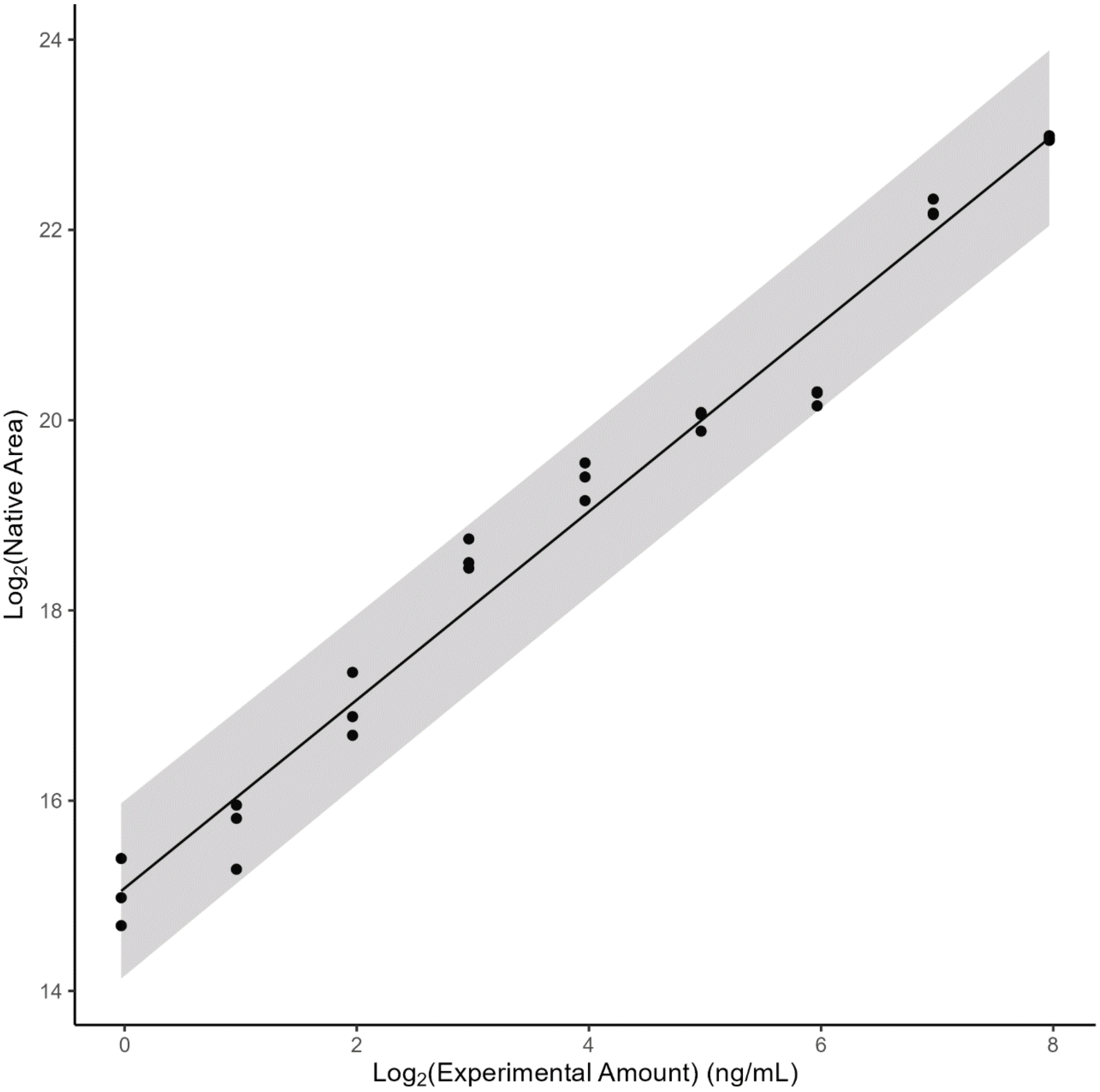

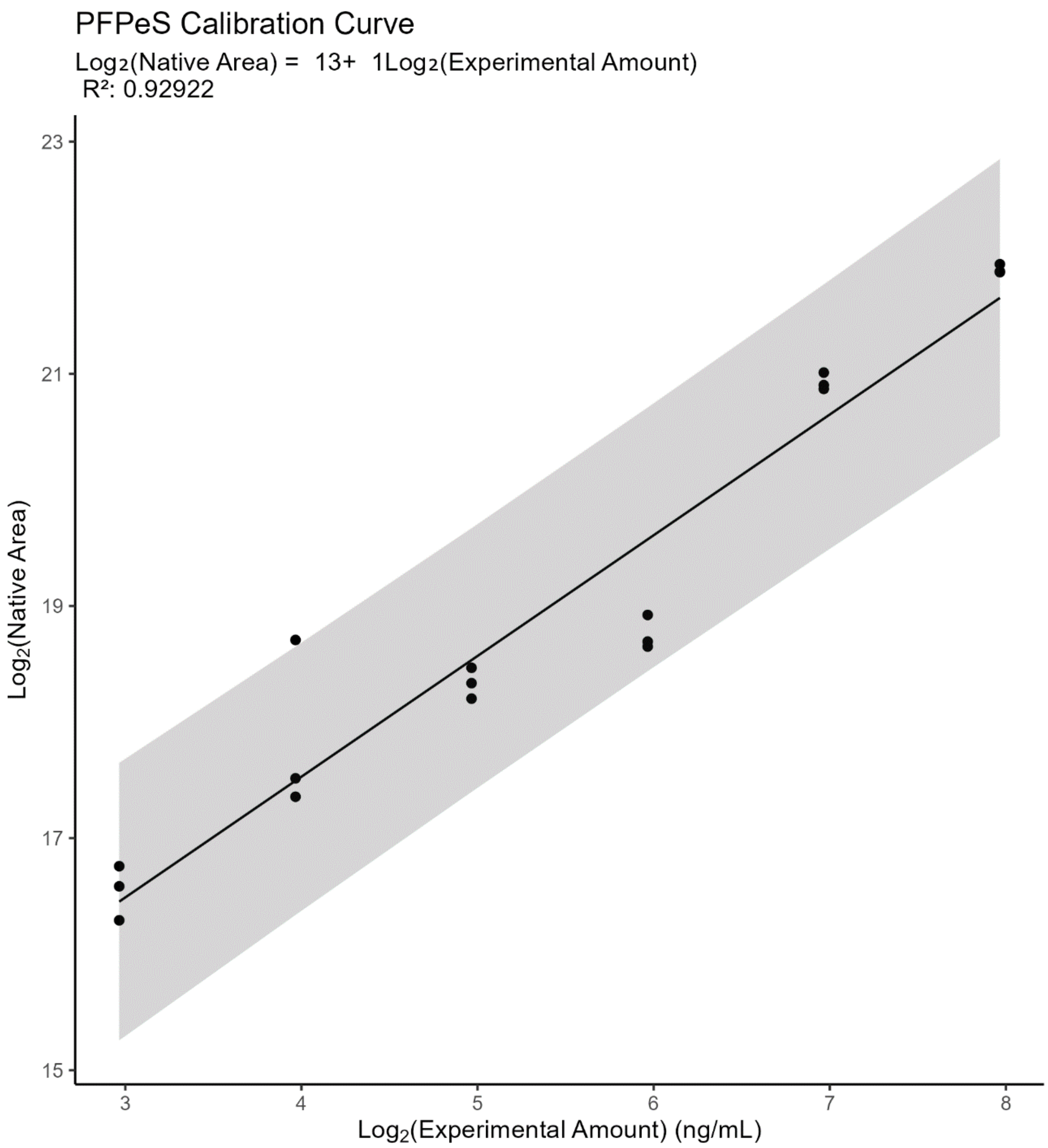
PFPeS Calibration Curve
Log₂(Native Area) = 13+ 1Log₂(Experimental Amount)
R²: 0.92922
23
21
19
17
15
Log2(Native Area)
3
4
5
6
7
8
Log2(Experimental Amount) (ng/mL)

PFTeDA Calibration Curve

Log_2(Native Area) = 22+0.94Log_2(Experimental Amount)

R^2: 0.92662

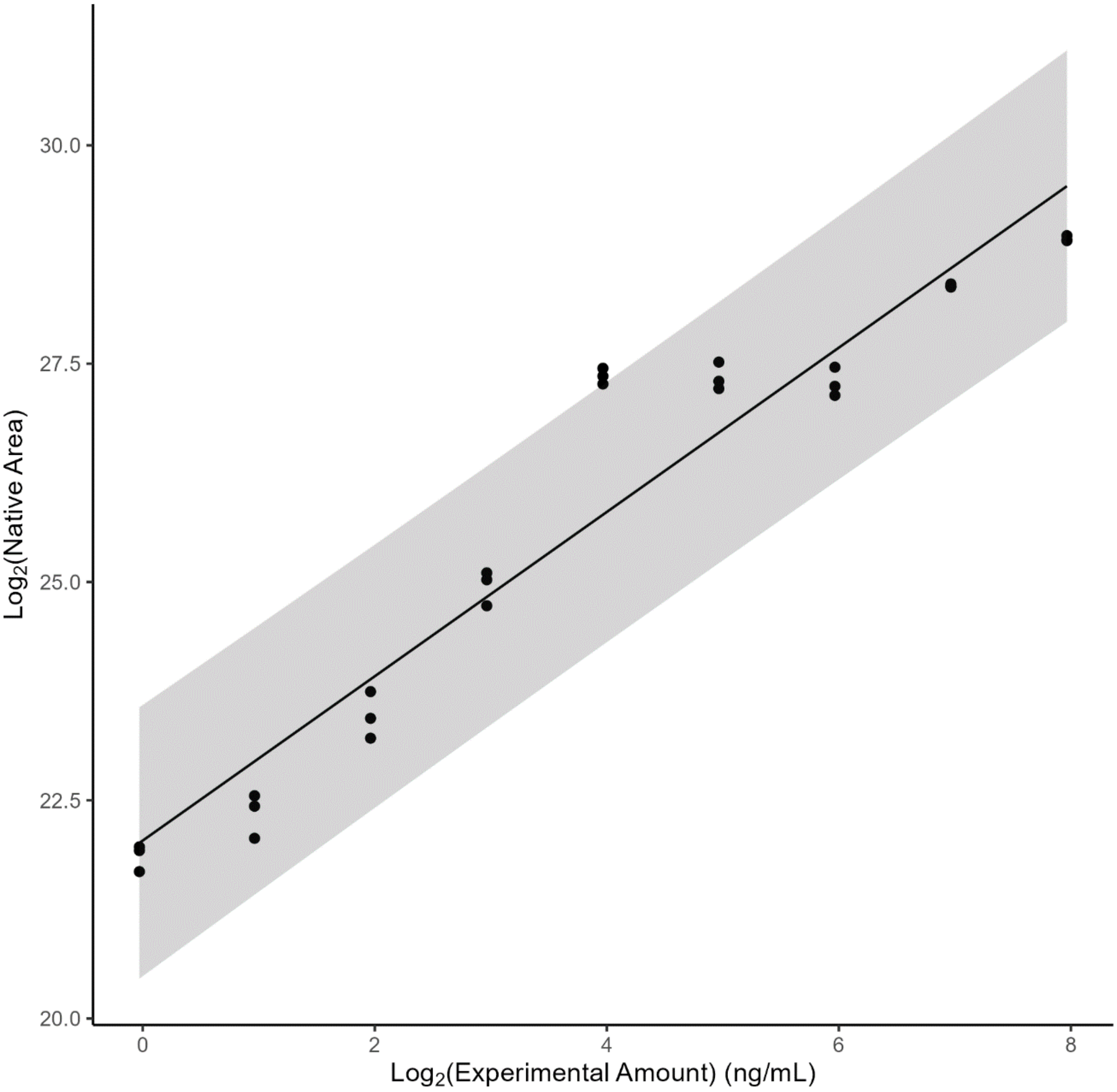

PFTrDA Calibration Curve

$Log_2(\text{Native Area}) = 22+0.89Log_2(\text{Experimental Amount})$

R^2: 0.97672

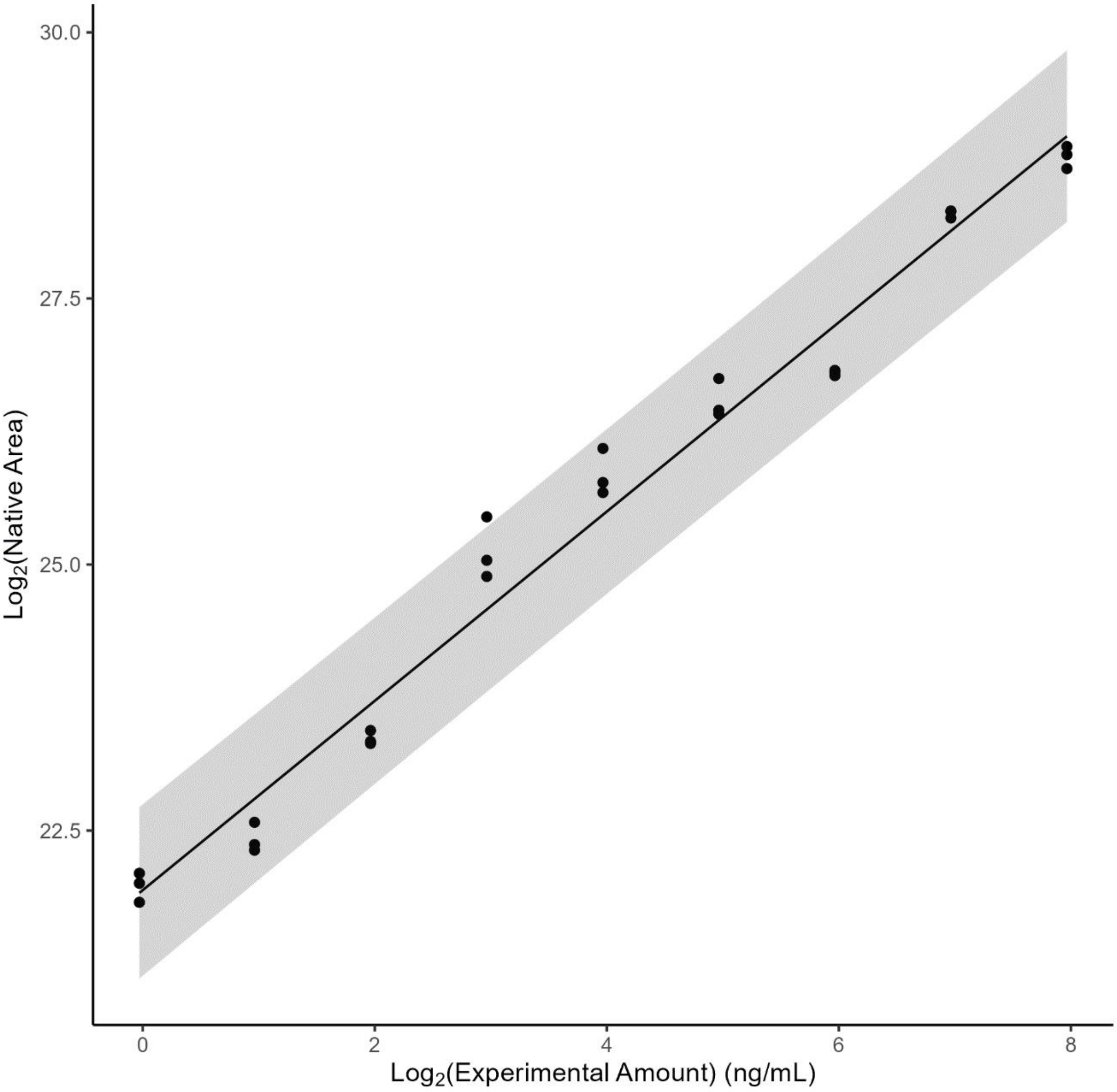

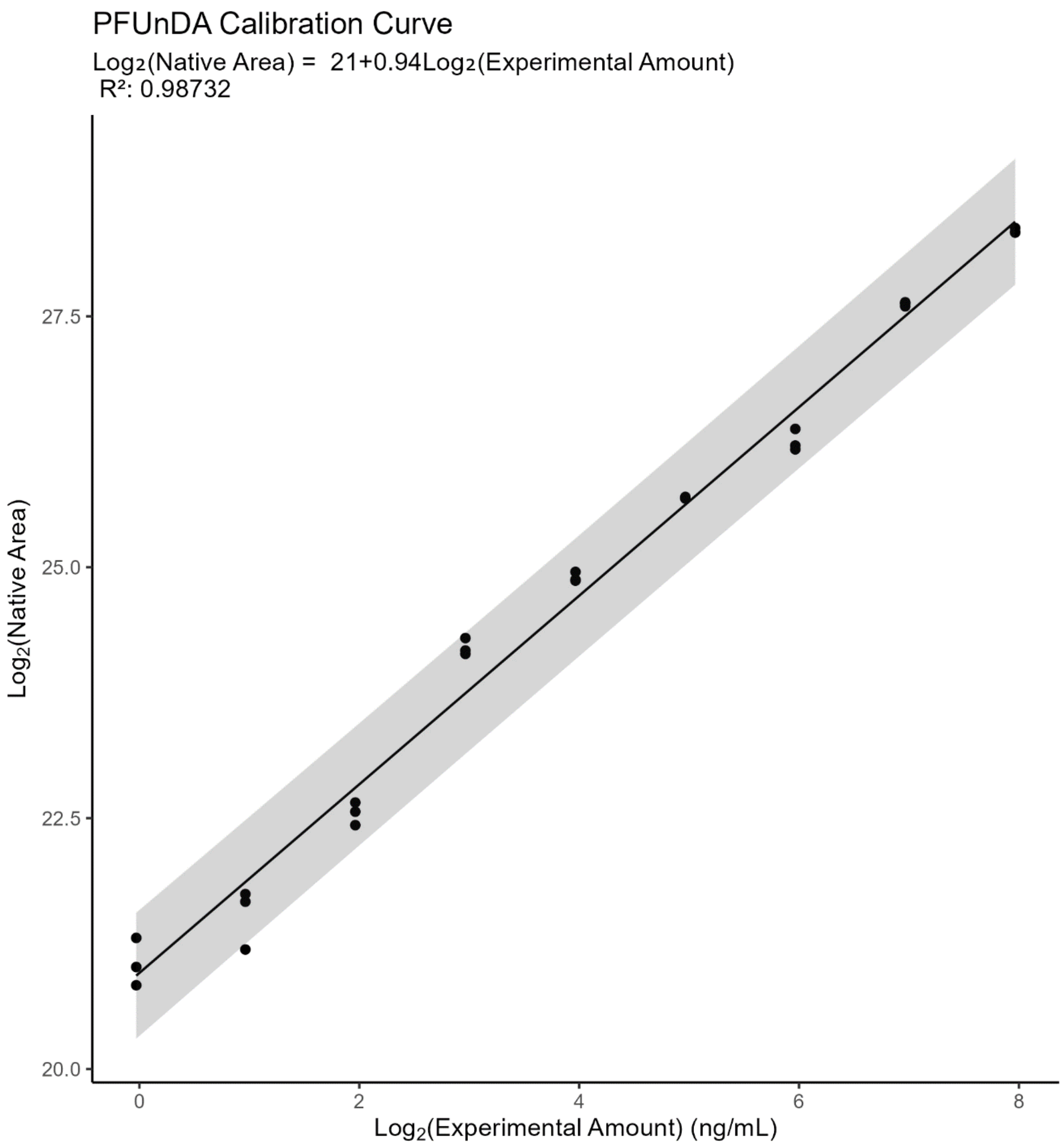
PFUnDA Calibration Curve
Log₂(Native Area) = 21+0.94Log₂(Experimental Amount)
R²: 0.98732
27.5
25.0
22.5
20.0
Log2(Native Area)
0
2
4
6
8
Log2(Experimental Amount) (ng/mL)
