# Supplementary material for: Establishing performance metrics for quantitative non-targeted analysis: a demonstration using per- and polyfluoroalkyl substances
Source: Anal Bioanal Chem. 2024 Jan 30;416(5):1249–67. doi: 10.1007/s00216-023-05117-4 (PMC10850229; doi:10.1007/s00216-023-05117-4)
Supplement: Supplementary file 1 — Supplementary file1 (ZIP 7749 KB) [file 216_2023_5117_MOESM1_ESM.zip › Pu_et_al_2023_qNTA_metrics_Supplementary_File_4.pdf]

Supplementary File 4

Establishing performance metrics for quantitative non-targeted analysis: a demonstration using per- and poly-fluoroalkyl substances

Shirley Pu[1,2*], James P. McCord[3*], Jacqueline Bangma[3], Jon R. Sobus[1*]

[1]U.S Environmental Protection Agency, Office of Research and Development, Center for Computational Toxicology and Exposure, 109 TW Alexander Dr., Research Triangle Park, NC 27711, USA

[2]Oak Ridge Institute for Science and Education (ORISE) Participant, 109 T.W Alexander Drive, Research Triangle Park, NC 27711, USA

[3]U.S Environmental Protection Agency, Office of Research and Development, Center for Environmental Measurement and Modeling, 109 TW Alexander Dr., Research Triangle Park, NC 27711, USA

*Authors to whom correspondence should be addressed:

Shirley Pu (pu.shirley@epa.gov); ORCID: 0000-0002-0122-3797

James McCord (mccord.james@epa.gov); ORCID: 0000-0002-1780-4916

Jon Sobus (sobus.jon@epa.gov); ORCID: 0000-0003-0740-6604

Search name: 10ppm extract

Search description: Untargeted environmental research ID workflow without statistics: Detect and identify unknown compounds.

- Performs retention time alignment, unknown compound detection, and compound grouping across all samples. Predicts elemental compositions for all compounds, and hides chemical background (using Blank samples). Identifies compounds using mzCloud (ddMS2 and/or DIA), ChemSpider (exact mass or formula) and local database searches against Mass Lists (exact mass with or without RT). Performs spectral similarity search against mzCloud for compounds with ddMS2. Applies mzLogic to rank order structure candidates from ChemSpider and mass list matches. Applies spectral distance scoring to mass list and ChemSpider matches. Generates mass defect values in the Compounds table based on selected mass defect type (Kendrick for identifying polymers).

Search date: 6/7/2022 2:29:15 PM

Created with Discoverer version: 3.3.0.550

[Input Files (0)]

-->Select Spectra (38)

[Select Spectra (38)]

-->Align Retention Times (ChromAlign) (50)

[Align Retention Times (ChromAlign) (50)]

-->Detect Compounds (51)

[Detect Compounds (51)]

-->Group Compounds (25)

-->Merge Features (14)

[Group Compounds (25)]

-->Assign Compound Annotations (40)

-->Search mzCloud (49)

-->Predict Compositions (37)

-->Search Mass Lists (39)

-->Fill Gaps (52)

-->Calculate Mass Defect (46)

[Search Mass Lists (39)]

-->Apply mzLogic (41)

-->Apply Spectral Distance (44)

[Fill Gaps (52)]

-->Mark Background Compounds (43)

[Assign Compound Annotations (40)]

[Search mzCloud (49)]

[Predict Compositions (37)]

[Apply mzLogic (41)]

[Apply Spectral Distance (44)]

[Mark Background Compounds (43)]

[Calculate Mass Defect (46)]

[Merge Features (14)]

Processing node 0: Input Files

Input Data:

- File Name(s) (Hidden):

E:\Compound Discover\Shirley\Cal0-98-1.raw

E:\Compound Discover\Shirley\Cal0-98-2.raw

E:\Compound Discover\Shirley\Cal0-98-3.raw

E:\Compound Discover\Shirley\Cal1-95-1.raw

E:\Compound Discover\Shirley\Cal1-95-2.raw

E:\Compound Discover\Shirley\Cal1-95-3.raw

E:\Compound Discover\Shirley\Cal3-90-1.raw

E:\Compound Discover\Shirley\Cal3-90-2.raw

E:\Compound Discover\Shirley\Cal3-90-3.raw

E:\Compound Discover\Shirley\Cal7-81-1.raw

E:\Compound Discover\Shirley\Cal7-81-2.raw

E:\Compound Discover\Shirley\Cal7-81-3.raw

E:\Compound Discover\Shirley\Cal15-63-1.raw

E:\Compound Discover\Shirley\Cal15-63-2.raw

E:\Compound Discover\Shirley\Cal15-63-3.raw

E:\Compound Discover\Shirley\Cal31-25-1.raw

E:\Compound Discover\Shirley\Cal31-25-2.raw

E:\Compound Discover\Shirley\Cal31-25-3.raw

E:\Compound Discover\Shirley\Cal62-5-1.raw

E:\Compound Discover\Shirley\Cal62-5-2.raw

E:\Compound Discover\Shirley\Cal62-5-3.raw

E:\Compound Discover\Shirley\Cal125-1.raw

E:\Compound Discover\Shirley\Cal125-2.raw

E:\Compound Discover\Shirley\Cal125-3.raw

E:\Compound Discover\Shirley\Cal250-1.raw

E:\Compound Discover\Shirley\Cal250-2.raw

E:\Compound Discover\Shirley\Cal250-3.raw

E:\Compound Discover\Shirley\DB-1.raw

E:\Compound Discover\Shirley\DB-2.raw

E:\Compound Discover\Shirley\DB-3.raw

E:\Compound Discover\Shirley\DB-Spike.raw

E:\Compound Discover\Shirley\Spike100.raw

Processing node 38: Select Spectra

----

1. Spectrum Properties Filter:

- Lower RT Limit: 0

- Upper RT Limit: 0

- First Scan: 0

- Last Scan: 0

- Ignore Specified Scans: (not specified)

- Lowest Charge State: 0

- Highest Charge State: 0

- Min. Precursor Mass: 50 Da

- Max. Precursor Mass: 5000 Da

- Total Intensity Threshold: 0

- Minimum Peak Count: 1

2. Scan Event Filters:

- Mass Analyzer: (not specified)

- MS Order: Any

- Activation Type: (not specified)

- Min. Collision Energy: 0

- Max. Collision Energy: 1000

- Scan Type: Any

- Polarity Mode: (not specified)

- MS1 Mass Range: (not specified)

- FAIMS CV: (not specified)

3. Peak Filters:

- S/N Threshold (FT-only): 3

4. Replacements for Unrecognized Properties:

- Unrecognized Charge Replacements: 1

- Unrecognized Mass Analyzer Replacements: ITMS

- Unrecognized MS Order Replacements: MS2

- Unrecognized Activation Type Replacements: CID

- Unrecognized Polarity Replacements: +

- Unrecognized MS Resolution@200 Replacements: 60000

- Unrecognized MSn Resolution@200 Replacements: 30000

5. General Settings:

- Precursor Selection: Use MS(n - 1) Precursor

- Use Isotope Pattern in Precursor Reevaluation: True

- Provide Profile Spectra: Automatic

- Store Chromatograms: False

Processing node 50: Align Retention Times (ChromAlign)

----

1. General Settings:

- Reference File: Cal125-1

Processing node 51: Detect Compounds

----

1. General Settings:

- Mass Tolerance [ppm]: 10 ppm

- Min. Peak Intensity: 1000000

- Min. # Scans per Peak: 5

- Use Most Intense Isotope Only: True

2. Trace Detection:

- Max. Number of Gaps to Correct: 2

- Min. Number of Adjacent Non-Zeros: 2

3. Peak Detection:

- Chromatographic S/N Threshold: 1.5

- Remove Baseline: False

- Gap Ratio Threshold: 0.35

- Max. Peak Width [min]: 2

- Min. Relative Valley Depth: 0.25

4. Isotope Pattern Detection:

- Group Isotopes for: Br; Cl

- Use Peak Quality for Isotope Grouping: True

- Filter out Features with Bad Peaks Only: True

- Zig-Zag Index Threshold: 0.2

- Jaggedness Threshold: 0.4

- Modality Threshold: 0.9

- Remove Potentially False Positive Isotopes: False

5. Compound Detection:

- Ions:

    [2M-H]-1

    [M+Cl]-1

    [M-CO2-H]-1

    [M-H]-1

    [M-H-H2O]-1

- Base Ions: [M-H]-1

- Remove Singlets: True

6. AcquireX Settings:

- Detect Persistent Background Ions: False

Processing node 25: Group Compounds

----

1. General Settings:

- Mass Tolerance: 10 ppm

- RT Tolerance [min]: 1

- Align Peaks: True

- Preferred Ions: [M+H]+1; [M+NH4]+1; [M-H]-1

- Area Integration: Most Common Ion

2. Peak Rating Contributions:

- Area Contribution: 3

- CV Contribution: 10

- FWHM to Base Contribution: 5

- Jaggedness Contribution: 5

- Modality Contribution: 5

- Zig-Zag Index Contribution: 5

3. Peak Rating Filter:

- Peak Rating Threshold: 6

- Number of Files: 1

Processing node 40: Assign Compound Annotations

----

1. General Settings:

- Mass Tolerance: 5 ppm

2. Data Sources:

- Data Source #1: mzCloud Search

- Data Source #2: mzVault Search

- Data Source #3: MassList Search

- Data Source #4: Predicted Compositions

- Data Source #5: ChemSpider Search

- Data Source #6: (not specified)

- Data Source #7: (not specified)

3. Scoring Rules:

- Use mzLogic: True

- Use Spectral Distance: True

- SFit Threshold: 20

- SFit Range: 20

4. Reprocessing:

- Clear Names: False

Processing node 49: Search mzCloud

----

1. General Settings:

- Compound Classes: All

- Precursor Mass Tolerance: 10 ppm

- FT Fragment Mass Tolerance: 10 ppm

- IT Fragment Mass Tolerance: 0.4 Da

- Library: Autoprocessed; Reference

- Post Processing: Recalibrated

- Max. # Results: 10

- Annotate Matching Fragments: True

- Search MSn Tree: False

2. DDA Search:

- Identity Search: Cosine

- Match Activation Type: True

- Match Activation Energy: Match with Tolerance

- Activation Energy Tolerance: 20

- Apply Intensity Threshold: True

- Similarity Search: None

- Match Factor Threshold: 30

3. DIA Search:

- Use DIA Scans for Search: True

- Max. Isolation Width [Da]: 500

- Match Activation Type: False

- Match Activation Energy: Any

- Activation Energy Tolerance: 100

- Apply Intensity Threshold: True

- Match Factor Threshold: 20

Processing node 37: Predict Compositions

----

1. Prediction Settings:

- Mass Tolerance: 5 ppm

- Min. Element Counts: C H

- Max. Element Counts: C90 H190 Br3 Cl8 F30 N10 O18 P3 S5

- Min. RDBE: 0

- Max. RDBE: 20

- Min. H/C: 0

- Max. H/C: 3.5

- Max. # Candidates: 10

- Max. # Internal Candidates: 25

2. Pattern Matching:

- Intensity Tolerance [%]: 30

- Intensity Threshold [%]: 0.1

- S/N Threshold: 3

- Min. Spectral Fit [%]: 30

- Min. Pattern Cov. [%]: 80

- Use Dynamic Recalibration: True

3. Fragments Matching:

- Use Fragments Matching: True

- Mass Tolerance: 5 ppm

- S/N Threshold: 3

Processing node 39: Search Mass Lists

----

1. Search Settings:

- Mass Lists: EFS HRAM Compound Database.masslist|Waterboards IS List.massList|DSSTox.massList

- Mass Tolerance: 5 ppm

- Use Retention Time: False

- RT Tolerance [min]: 0.5

Processing node 41: Apply mzLogic

----

1. Search Settings:

- FT Fragment Mass Tolerance: 10 ppm

- IT Fragment Mass Tolerance: 0.4 Da

- Max. # Compounds: 0

- Max. # mzCloud Similarity Results to consider per Compound: 10

- Match Factor Threshold: 30

Processing node 44: Apply Spectral Distance

----

1. Pattern Matching:

- Mass Tolerance: 5 ppm

- Intensity Tolerance [%]: 30

- Intensity Threshold [%]: 0.1

- S/N Threshold: 3

- Use Dynamic Recalibration: True

Processing node 52: Fill Gaps

----

1. General Settings:

- Mass Tolerance: 5 ppm

- S/N Threshold: 3

- Use Real Peak Detection: True

Processing node 43: Mark Background Compounds

----

1. General Settings:

- Max. Sample/Blank: 5

- Max. Blank/Sample: 0

- Hide Background: True

Processing node 46: Calculate Mass Defect

----

1. Mass Defect:

- Fractional Mass: False

- Standard Mass Defect: False

- Relative Mass Defect: False

- Kendrick Mass Defect: True

- Nominal Mass Rounding: Floor

2. Kendrick Formula:

- Formula 1: $C_2 F_4$

- Formula 2: $C_2 F_3 O$

- Formula 3: $C_2 H_4$

- Formula 4: $C_3 H_6$

- Formula 5: $C_8 H_8$

Processing node 14: Merge Features

----

1. Peak Consolidation:

- Mass Tolerance: 5 ppm

- RT Tolerance [min]: 0.25
